# Transient and stabilized complexes of Nsp7, Nsp8, and Nsp12 in SARS-CoV-2 replication

**DOI:** 10.1016/j.bpj.2021.06.006

**Published:** 2021-06-29

**Authors:** Mateusz Wilamowski, Michal Hammel, Wellington Leite, Qiu Zhang, Youngchang Kim, Kevin L. Weiss, Robert Jedrzejczak, Daniel J. Rosenberg, Yichong Fan, Jacek Wower, Jan C. Bierma, Altaf H. Sarker, Susan E. Tsutakawa, Sai Venkatesh Pingali, Hugh M. O’Neill, Andrzej Joachimiak, Greg L. Hura

**Affiliations:** 1 Center for Structural Genomics of Infectious Diseases, Consortium for Advanced Science and Engineering, University of Chicago, Chicago, Illinois; 2 Department of Biochemistry and Molecular Biology, University of Chicago, Chicago, Illinois; 3 Molecular Biophysics and Integrated Bioimaging Division, Lawrence Berkeley National Lab, Berkeley, California; 4 Center for Structural Molecular Biology, Neutron Scattering Division, Oak Ridge National Laboratory, Oak Ridge, Tennessee; 5 Structural Biology Center, X-ray Science Division, Argonne National Laboratory, Argonne, Illinois; 6 Graduate Group in Biophysics, University of California, Berkeley, Berkeley, California; 7 Department of Animal Sciences, Auburn University, Auburn, Alabama; 8 Chemistry and Biochemistry Department, University of California Santa Cruz, Santa Cruz, California

**Keywords:** SAXS, SANS, Crystallography, SARS-CoV-2, Transcription

## Abstract

The replication transcription complex (RTC) from the virus SARS-CoV-2 is responsible for recognizing and processing RNA for two principal purposes. The RTC copies viral RNA for propagation into new virus and for ribosomal transcription of viral proteins. To accomplish these activities, the RTC mechanism must also conform to a large number of imperatives, including RNA over DNA base recognition, basepairing, distinguishing viral and host RNA, production of mRNA that conforms to host ribosome conventions, interfacing with error checking machinery, and evading host immune responses. In addition, the RTC will discontinuously transcribe specific sections of viral RNA to amplify certain proteins over others. Central to SARS-CoV-2 viability, the RTC is therefore dynamic and sophisticated. We have conducted a systematic structural investigation of three components that make up the RTC: Nsp7, Nsp8, and Nsp12 (also known as RNA-dependent RNA polymerase). We have solved high-resolution crystal structures of the Nsp7/8 complex, providing insight into the interaction between the proteins. We have used small-angle x-ray and neutron solution scattering (SAXS and SANS) on each component individually as pairs and higher-order complexes and with and without RNA. Using size exclusion chromatography and multiangle light scattering-coupled SAXS, we defined which combination of components forms transient or stable complexes. We used contrast-matching to mask specific complex-forming components to test whether components change conformation upon complexation. Altogether, we find that individual Nsp7, Nsp8, and Nsp12 structures vary based on whether other proteins in their complex are present. Combining our crystal structure, atomic coordinates reported elsewhere, SAXS, SANS, and other biophysical techniques, we provide greater insight into the RTC assembly, mechanism, and potential avenues for disruption of the complex and its functions.

## Significance

The SARS-CoV-2 virus has been implicated in 3,000,000 deaths. No therapeutic has been developed with sufficient efficacy to change the course of the pandemic. The single US-approved therapeutic targets the replication transcription complex (RTC), which is responsible for copying the viral RNA genome to pass to new virus. Here, we structurally characterize three components of the RTC with and without RNA using crystallography and solution small-angle scattering. Combining the structural information on each component on its own and in combinatorial mixtures, we develop an understanding of how the RTC assembles. This understanding provides insights into the RTC functions and assembly processes that could be inhibited. This impacts not only SARS-CoV-2 but most RNA viruses.

## Introduction

The severe acute respiratory syndrome coronavirus 2 (SARS-CoV-2) virus has plagued every populated continent and has been implicated in 3,000,000 deaths at the time of writing. Recently deployed vaccines have provided much hope. However, distribution, voluntary immunization, and mutations in the virus remain major concerns. The development and application of treatments that attack fundamental viral machinery will remain critical for the foreseeable future.

In the US thus far, a single drug, remdesivir, has been conditionally approved for specifically targeting the virus. It targets the RNA-dependent RNA polymerase (RdRp) (1). SARS-CoV-2 RdRp, also referred to as nonstructural protein 12 (Nsp12), is the catalytic center of the macromolecular complex frequently referred to as the replication transcription complex (RTC). The RTC is essential for virus replication because it makes copies of genomic and subgenomic RNAs and polymerizes antisense RNA. After maturation, these RNAs serve as mRNAs to produce virus Nsps, which are structural and accessory proteins. Copies of genomic RNA are eventually packaged into mature virions that bud out of host cells. The remdesivir triphosphate is incorporated into newly synthesized RNA by RdRp and either stalls or interrupts viral RNA polymerization.

Although the coronavirus genome is larger than most viruses (~30 kbs), it remains 100,000 times smaller than that of humans. The highly evolved and efficient SARS-CoV2 RTC is a major part of how the virus overcomes the limitations of its genome size. The RTC specifically identifies and polymerizes viral over host RNA without activating host defenses. The RTC contextually balances synthesis of subgenomic RNAs for use in translation of viral proteins and polymerization of genomic RNA for new virus maturation. The subgenomic RNA produced must be 3′ polyadenylated and 5′ capped, conforming to host ribosome mRNA conventions. The RTC can discontinuously read long RNAs and produce shorter subgenomic RNAs that serve as mRNAs that code for structural and accessory proteins within the long RNA (discontinuous transcription) (2). The mechanisms must be robust to varied attacks by the host cell defenses. All this must be done while retaining primary polymerase activities of recognizing initiation and termination sequences, discriminating DNA versus RNA bases, and basepairing. Reverse engineering this complex macromolecule is likely to be a challenge. However, as a central mechanism with so many functions, several avenues for sabotage by small molecules or proteins may be possible beyond the one exploited by remdesivir.

In SARS-CoV-2, besides RdRp (Nsp12), the RTC involves nonstructural proteins Nsp7, Nsp8, and others (3, 4, 5). For an excellent review that largely summarized our understanding before the pandemic, see Snijder et al. (6). Nsp12 is the largest component of the complex, and Nsp7 and Nsp8 are considered cofactors.

Several additional functions have been reported for Nsp7 and 8. Both Nsp7 and 8 are transcribed at higher rates relative to Nsp12 as part of open reading frame 1a and earlier in the infection so they may act to prepare the cell for viral replication. In a thorough study, Nsp8 has been shown to polyadenylate RNA (7), which could play a role in 3′ mRNA preparation. Nsp8 has also been implicated in blocking ribosomal membrane protein recognition signals (8). Crystal structures and other supporting methods have visualized Nsp7/8 in a ring-like hexadecamer (9), which provocatively suggests a processive sliding complex on double-stranded RNA with a possibly independent role.

Our understanding of the RTC has been remarkably advanced through recent cryo-electron microscopy (cryo-EM) studies resolving structures of Nsp7/8/12 with and without RNA (1,4,10,11). Most recently, structures of Nsp7/8/12/13 (12,13) and Nsp7/8/9/12/13 (14) have been reported. However, many questions remain about connecting the cryo-EM structures with the assembly, the role of cofactors, and the mechanism. The integration of many structural, biochemical, and genomics studies will be required to provide a mechanistic and actionable model of each protein contributing to the RTC and to assess their assembly, varied functions, and potential vulnerabilities.

**Figure 1:**
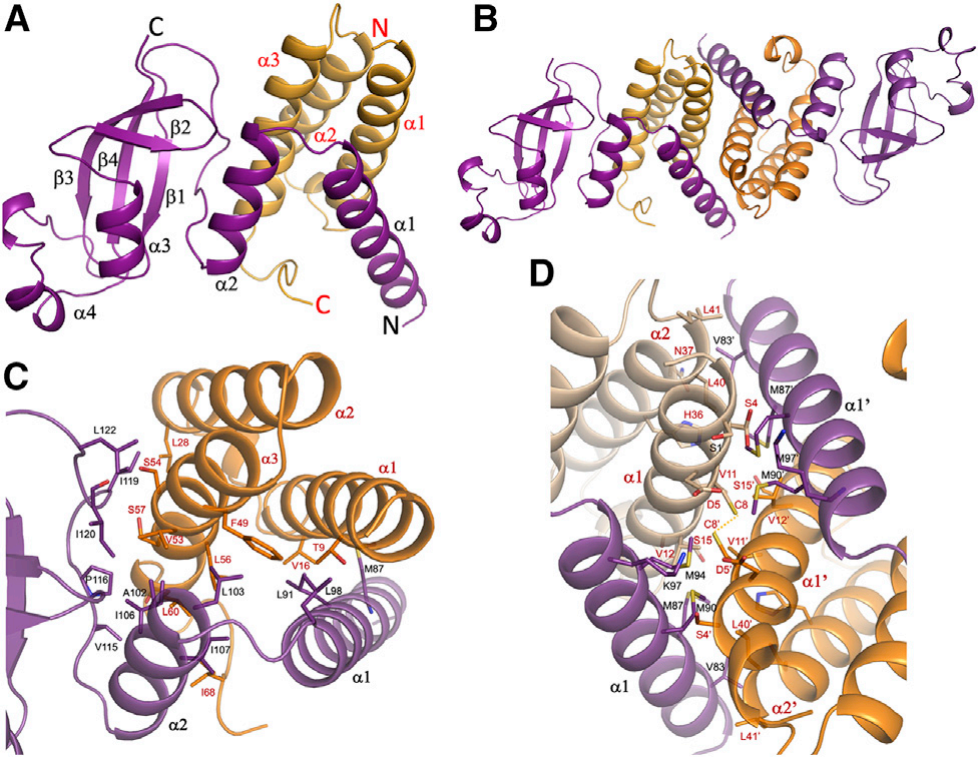
Structure of Nsp7/8 complex. The crystallized structure of the heterodimer Nsp7/8 (*A*) shows Nsp7’s (*orange*) C-terminal helices intercalated between Nsp8’s (*purple*) long *α*1 N-terminal helix (truncated in our structure) and *α*2. The heterotetramer structure (*B*) is also shown and present in all three of our crystal forms. Details of the heterodimer interface are shown in (*C*). Details of the heterotetramer interface are shown in (*D*), with interacting residues shown by sticks labeled red for Nsp7 and labeled black for Nsp8. An interchain, symmetrically formed disulfide bond in Nsp7 formed by C8 is shown in dashes.

**Figure 2:**
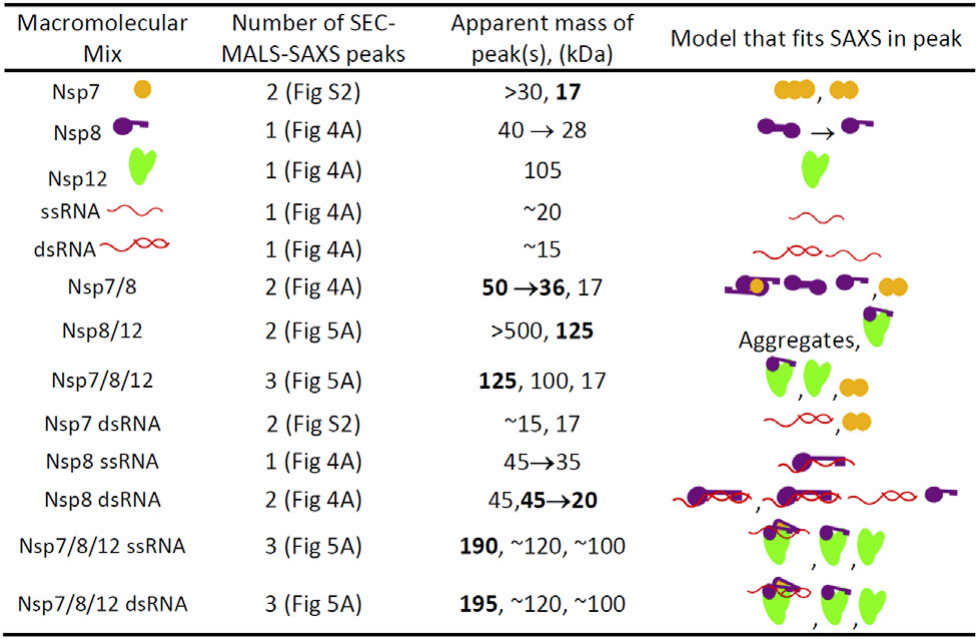
Nsp7, Nsp8, Nsp12, and RNA complexes found in elution profiles from SEC-MALS-SAXS. Each molecule was measured independently and is represented pictorially (*top left*). The number of peaks and the figure associated with the elution profile is indicated in the second column. The apparent mass through each peak is indicted in the third column. When an elution has more than one peak, the largest peak is indicated by bold mass. In some peaks, the mass changes across the peak. Mass values prefaced with ~ indicate weak signals by MALS because of low abundance. For comparison, the calculated masses for monomeric Nsp7, Nsp8, and Nsp12 are 9, 24, and 100 kDa respectively. The rightmost column depicts mixtures of models that fit the SAXS data through analysis described in the remainder of the text.

Toward the goal of understanding the macromolecular machinery of the RTC, we have undertaken a systematic study of individual components and their complexes. We have solved a 1.5 Å x-ray macromolecular crystal structure of the complex between Nsp7 and Nsp8 (Fig. 1). This structure, along with those solved by others (15), particularly the recent cryo-EM structures of the Nsp7/8/12 RNA complex (10), provided key information for the interpretation of our solution scattering measurements. We have used solution scattering along with other biochemical techniques to study each protein in isolation, pairs (Nsp7/8, Nsp8/12, Nsp7-RNA, and Nsp8-RNA), ternary complexes, and all four components together (Nsp7/8/12 RNA) (Figs. 2, 4, 5, and 6).

**Figure 3:**
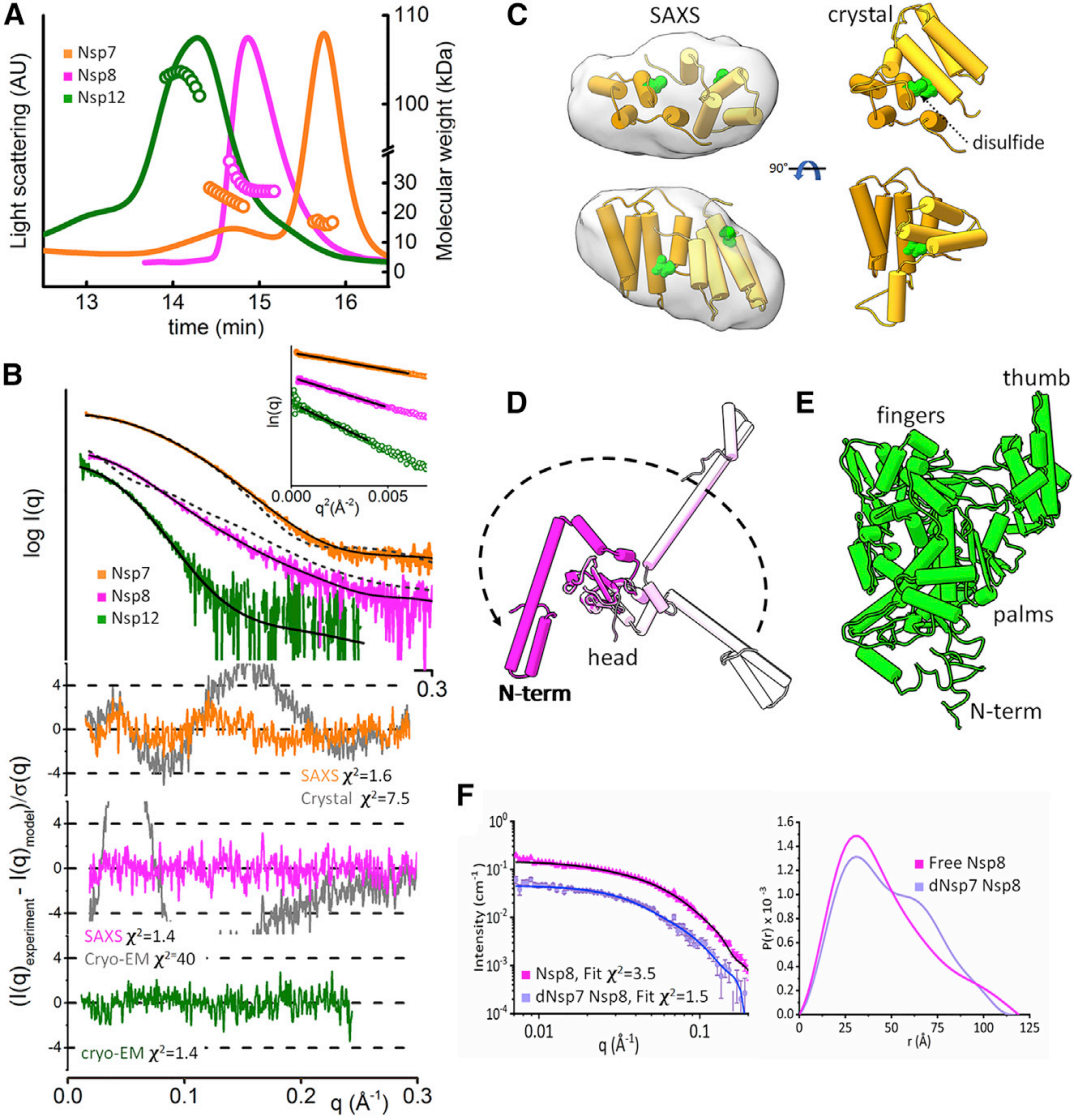
Solution states of independent Nsp7, Nsp8, and Nsp12 (*A*) SEC-MALS-SAXS elution profiles for Nsp7 (*orange*), Nsp8 (*purple*), and Nsp12 (*green*) by light scattering intensity (*solid lines*, *left axis*), with mass indicated by circles (*right axis*). (*B*) Experimental (*colored lines*) SAXS profiles for each protein. Guinier plots for experimental SAXS curves are shown in the inset. Calculated best-fit models (*solid black lines*) and alternate models from available structures (*dashed lines*) are shown along with residuals (*lower plot*, *gray* for alternate models) and goodness-of-fit parameter *χ*^2^. (*C*) Best-fit model for Nsp7 is an alternate dimer than that found in our crystal structure, with the disulfide forming Cys8 shown in green. The average SAXS envelop is superimposed on the SAXS model. (*D*) The Nsp8 monomer is found in a thus far unobserved conformation (*dark magenta*) relative to the N-terminal (N-term) domain in the superimposed atomically resolved cryo-EM structures (*pink*) (PDB: 6YYT). (*E*) Nsp12 measurements agree with available atomic structures (PDB: 6YYT). (*F*) SANS profiles (*left*) were measured for Nsp8 (*magenta*) and dNsp7-Nsp8 complex (*light blue circles*) in 90% D_2_O, masking dNsp7. Fits to models described in the text are shown in black and blue, respectively. The P(r) calculated from SANS for both (*right plot*) shows Nsp7 alters Nsp8 structure.

**Figure 4:**
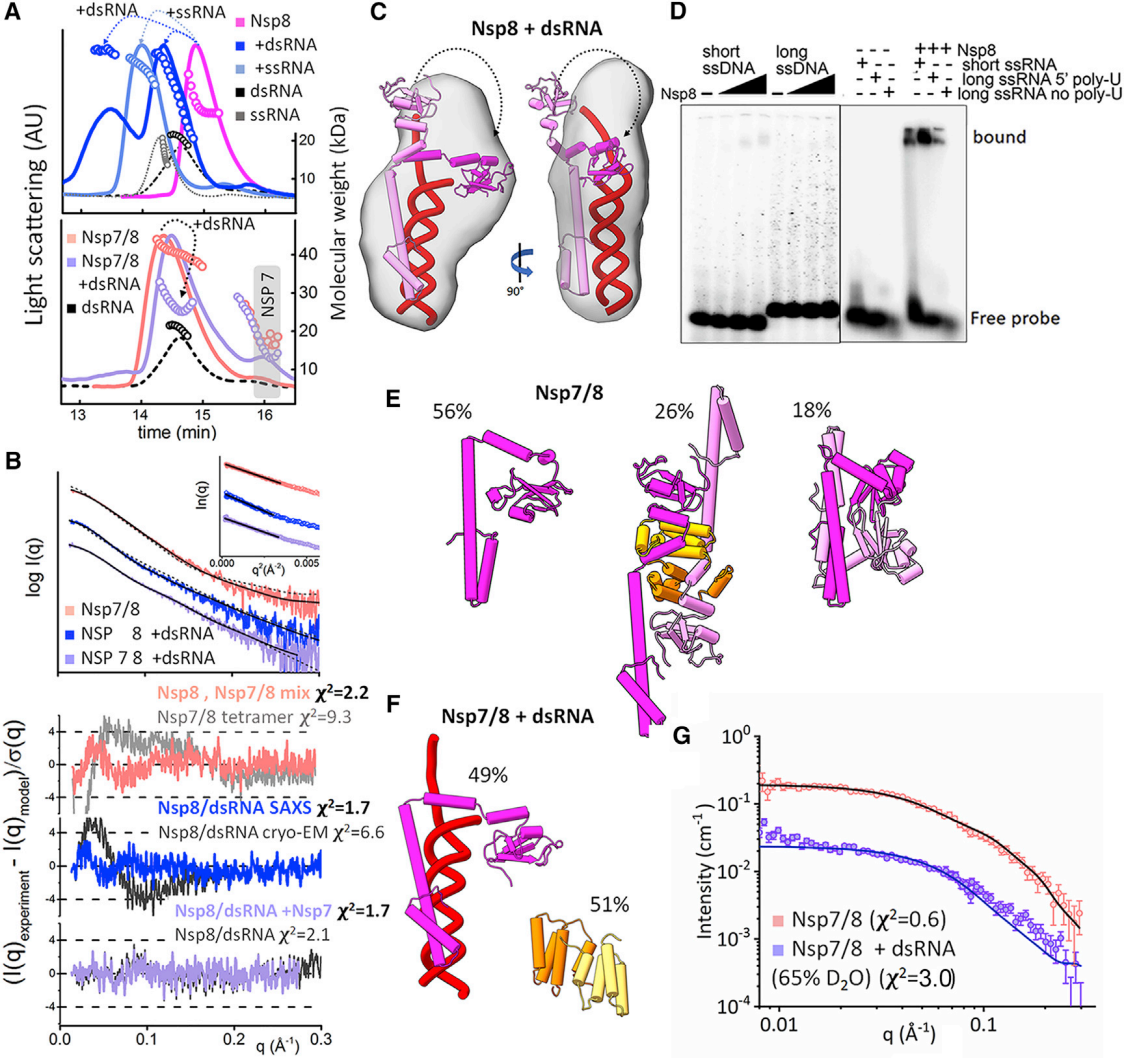
Complexation of Nsp8 and Nsp7/8 with the nucleic acid. (*A*) SEC-MALS chromatograms for Nsp8, Nsp8 + dsRNA, Nsp8 + ssRNA, dsRNA, and ssRNA (*top*) and Nsp7/8, Nsp7/8 + dsRNA, and dsRNA (*bottom*) are colored as indicated. Solid lines represent the light scattering in detector units (*left axis*), and symbols represent molecular mass versus elution time (*right axis*). (*B*) Experimental SAXS profiles for Nsp7/8, Nsp8 + dsRNA, and Nsp7/8 + dsRNA collected at the SEC peak shown together with calculated SAXS profiles from best fitting atomic models (*black line*) or alternative model (*dash line*). Guinier plots for experimental SAXS curves are shown in the inset. Residuals of best-fit models (colored as indicated), alternative models (*gray*), and goodness-of-fit values (*χ*^2^) are shown in bottom plot. (*C*) Solution model of Nsp8-dsRNA (*magenta* and RNA in *red*) used in the calculate SAXS profile in (*B*) with overlaid SAXS-based shape. (*D*) Nsp8 EMSA with radio-labeled polynucleotides shows no binding of ssDNA (*right*) and binding of all ssRNA substrates. (*E*) Ensemble of structures that fit Nsp7/8 used in the calculated SAXS profile in (*B*). Mass of each model is indicated. (*F*) The ensemble that fits the SAXS from Nsp7/8 + dsRNA with mass of each model indicated. (*G*) SANS data for the Nsp7/8/RNA complex (*pink circles*) and Nsp7/8/DNA in 65% D_2_O (*light blue circles*) were fit by the models shown in (*E*) and (*C*).

**Figure 5:**
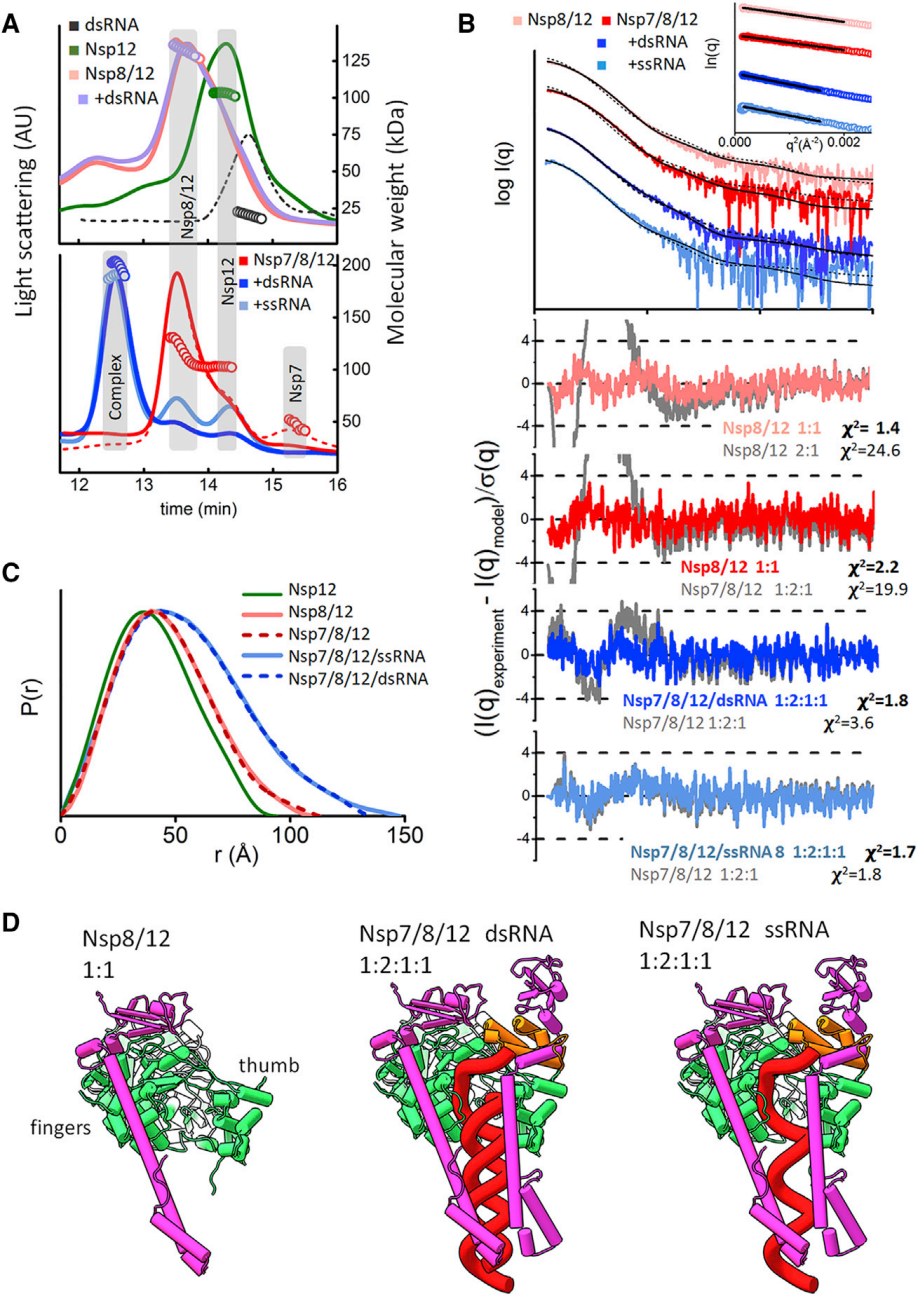
RNA-stabilized Nsp7/8/12 complex. (*A*) SEC-MALS chromatograms for Nsp12, Nsp8/12, Nsp8/12 + dsRNA, and dsRNA (*top*) and Nsp7/8/12, Nsp7/8/12 + dsRNA, and Nsp7/8/12 + ssRNA (*bottom*) are colored as indicated. Solid lines represent the light scattering detector units, and symbols represent molecular mass versus elution time. (*B*) Experimental SAXS profiles for Nsp8/12, Nsp7/8/12, Nsp8/12 + dsRNA, and Nsp7/8/12 + ssRNA collected at the SEC peak are shown together with the theoretical SAXS profiles for best fitting models (*black line*) and alternative models (*dash line*). SAXS fits are shown together with the fit residuals for the solution-state model (colored as indicated), alternative model (*gray*), and goodness-of-fit values (*χ*^2^). Guinier plots for experimental SAXS curves are shown in the inset. (*C*) Normalized P(r) function for Nsp12, Nsp8/12, Nsp7/8/12, Nsp7/8/12 + dsRNA, and Nsp7/8/12 + ssRNA. The similarity of P(r) functions between Nsp8/12 and Nsp7/8/12 further confirms the absence of Nsp7 and one Nsp8 in the Nsp7/8/12 mixture. (*D*) Solution-state models for Nsp8/12, Nsp7/8/12 + dsRNA, and Nsp7/8/12 + ssRNA were used to fit experimental data shown in (*B*).

**Figure 6:**
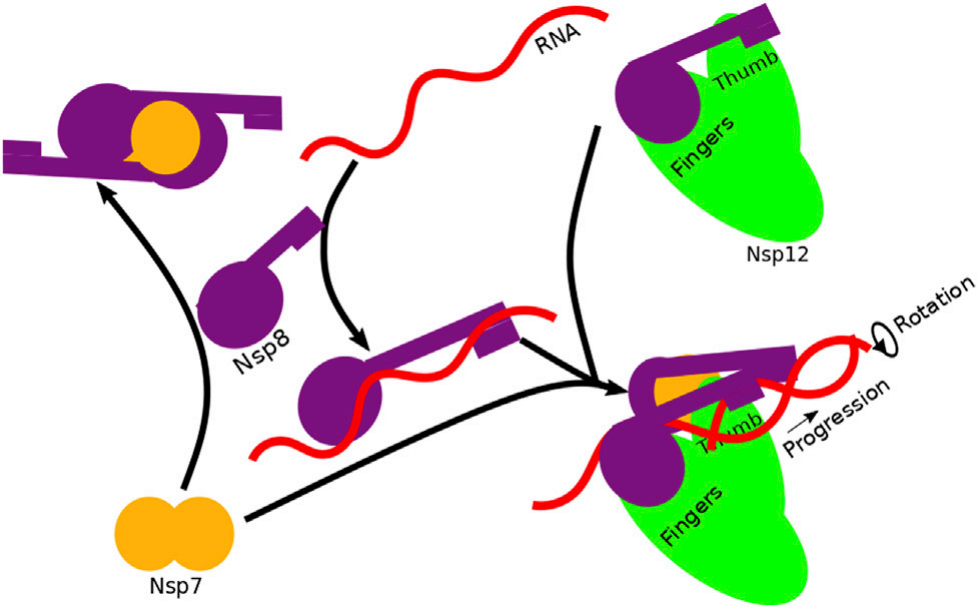
Assembly of the RTC components. The solved crystal structure (*top left*) reported in this work exists in a dynamic equilibrium and forms at high concentrations of Nsp7 and Nap8. Nsp7 is dominantly dimeric on its own (*bottom left*), though it can form linear oligomers. Nsp8 alone is in a compact conformation (*center*). However, this conformation becomes extended when RNA or Nsp7 binds in a competitive manner for available binding sites on Nsp8. When all three are combined with a stabilized form of 1:1 Nsp8/12, a very stable Nsp7/8/12 RNA complex forms in a 1:2:1:1 ratio for RNA transcription. The architecture, preferred binding sites for Nsp8 on the fingers domain, and strong binding of RNA by Nsp8 suggest a mechanism in which the Nsp8 on the thumb domain may swap positions with that on the fingers while the one on the fingers progresses with the RNA.

Because many proteins in the viral genome are expected to have multiple functions that require conformational or assembly changes, heterogeneities are expected in solution. All complexes were examined during elution from size exclusion chromatography (SEC) by multiangle light scattering (MALS) and small-angle x-ray scattering (SAXS): SEC-MALS-SAXS. MALS provides mass and SAXS informs on structure at every point in elution. Together, they detail the oligomeric and conformational heterogeneity characteristic of each mixture, complementing the cryo-EM and crystallographic results. To further interpret complexes, we also used small-angle neutron scattering (SANS) and its capability to contrast match out components within complexes. Using these and other biochemical techniques, we provide insights into the function, the assembly process, and the dynamics and identify stable complexes that are tractable for further mechanistic interrogation.

## Materials and methods

Further detailed methods are provided in the Supporting materials and methods.

### Gene cloning, protein expression, and purification

NCBI reference sequences for Nsp7, Nsp8, and Nsp12 are YP_009725303.1, YP_009725304.1, and YP_009725307.1, respectively, and are further available in the Supporting materials and methods. SARS-CoV-2 (taxid: 2697049) genes were expressed in *Escherichia coli* (16) and included N-terminal His_6_ (histidine)-TEV (tobacco etch mosaic virus) affinity tags that were subsequently cleaved. For SANS, deuterium-labeled Nsp7 was produced using previously described methods (17). Proteins were purified first via His_6_-tag using an Ni^+2^ column, with subsequent cleavage using TEV protease, and finally by SEC. Samples were concentrated and dialyzed using centrifugation and dialysis membranes.

### RNA and DNA constructs

The [^32^P]-labeled RNA used in the function testing extension assay was synthesized using previously described methods (18), folds into a hairpin, and has the sequence 5′-pppGGCUUAGGAGAUGAUGAAAGUCAUUCUCCU-OH-3′. The 36 base long single-stranded RNA (ssRNA) had the sequence (5′-UUU UCA UGC UAC GCG UAG CAU GCU ACG CGU AGC AUG-3′), and the 28 base short ssRNA had the sequence (5′-CAU GCU ACG CGU AGC AUG CUA CGC GUA G-3′). Both long and short ssRNA were ordered from IDT.

### Crystallization, data collection, and structural analysis of the Nsp7/8 complex

Crystallization trials using 400 nL volumes were performed with MCSG1, MCSG2, MCSG3, MCSG4, INDEX, Natrix high-throughput crystallizations screens (Anatrace, Hampton Research, Maumee, OH). The x-ray diffraction experiments were carried out at the Structural Biology Center 19-ID beamline at the Advanced Photon Source, Argonne National Laboratory. The data sets were processed and scaled with the HKL3000 suite (19), the Ctruncate program (20,21) from the CCP4 package (22), and molrep (23) using the SARS-CoV Nsp7/8 complex structure (Protein Data Bank (PDB): 5F22) as a search model. Further refinement used REFMAC (22,24), COOT, and PHENIX (25). Throughout the refinement, the same 5% of reflections were kept out from the refinement in both REFMAC and PHENIX refinement. The final structures converged to R_work_ = 0.218 and R_free_ = 0.252 for Nsp7/8A, R_work_ = 0.187 and R_free_ = 0.229 for Nsp7/8B, and R_work_ = 0.161 and R_free_ = 0.199 for Nsp7/8C with regards to the data quality of each structure. The stereochemistry of the structures was checked with PROCHECK (26) and the Ramachandran plot and validated with the PDB validation server. The data collection and processing statistics are given in Table S1. The atomic coordinates and structure factors have been deposited in the PDB under accession codes 6WIQ, 6WQD, and 6XIP for Nsp7/8A, Nsp7/8B, and Nsp7/8C, respectively.

### SEC-MALS-SAXS

SEC-MALS-SAXS data were collected at the advanced light source (ALS) beamline SIBYLS (beamline 12.3.1) in Berkeley, California (27,28). The x-ray wavelength was set at *λ* = 1.127 Å, and the sample-to-detector distance was 2070 mm, resulting in scattering vectors, q, ranging from 0.01 to 0.35 Å^−1^. The scattering vector is defined as q = 4*π*sin*θ*/*λ*, where 2*θ* is the scattering angle. All experiments were performed at 20°C, and data were processed as described (29). The subtracted frames were investigated by the radius of gyration (R_g_) derived by the Guinier approximation I(q) = I(0) exp(−q^2^R_g_^2^/3) with the limits q R_g_ < 1.5 (30). The elution peak was mapped by comparing the integral ratios to background and R_g_ relative to the recorded frame using the program SCÅTTER. The program SCÅTTER was used to compute the pair-distribution function (P(r)). P(r) functions were normalized based on the molecular mass of the assemblies as determined by SCÅTTER using the volume of correlation Vc (Table S2; (31)). The SAXS flow cell was additionally connected inline to a 1290 series ultraviolet-visible light diode array detector measuring at 280 and 260 nm (Agilent, Santa Clara, CA), 18-angle DAWN HELEOS II MALS and quasielastic light scattering, and Optilab rEX refractometer (Wyatt Technology, Santa Barbra, CA).

### High-throughput SAXS and SANS

SAXS data were collected in “batch” high-throughput mode at the ALS beamline 12.3.1 (SIBYLS) at Lawrence Berkeley National Laboratory (32) on Nsp8 at 10, 5, and 2.5 mg/mL because of the concentration dependence we observed in SEC-SAXS and differences from SANS data. Experiments were performed at 20°C as described elsewhere (27).

SANS measurements were collected at the Bio-SANS and EQ-SANS instruments located at the High Flux Isotope Reactor and Spallation Neutron Source, respectively, at Oak Ridge National Laboratory (33,34). The protein concentrations for SANS measurements were 1.5 mg/mL Nsp7/8, 4 mg/mL Nsp8, 4 mg/mL deuterated Nsp7/Nsp8 complex, and mixture of Nsp7/8/double-stranded RNA (dsRNA) in 1:1 molar ratio at 3.5 mg/mL Nsp7/8 complex in the same buffer as the SAXS experiments were performed in. For the contrast-matching SANS experiments of deuterated dNsp7 with protiated Nsp8 (dNsp7/Nsp8 complex), 90% D_2_O buffer was used to selectively highlight the scattering from Nsp8. Studies of the Nsp7/8/dsRNA mixture were performed in 65% D_2_O buffer to selectively highlight the scattering from the Nsp7/8 complex. Initial SANS data analysis, including Guinier fits and pair-distribution calculations, was performed using the BioXTAS RAW program and ATSAS suite (35,36). The SAXS-derived molecular mass was determined using the volume of Porod method as implemented in RAW (37).

### Solution structure modeling

Tools used to find conformations and assemblies of each of the structures included MODELER (38), BILBOMD (39), FOXS (40,41), MultiFOXS (42), FOXSDock (43), OLIGOMER (44), CRYSON (45), SAXS/REFMX (46,47), and GASBOR (48). To help build models that fitted the SAXS data, aside from those reported here, we relied on the following structures—PDB: 6YYT (10), PDB: 3UB0 (49), PDB: 2AHM. Full details are available in the Supporting materials and methods.

## Results

### Atomic-resolution crystal structure of Nsp7/8

To provide insights into the structures, key residues of association, and multimeric state between the SARS-CoV-2 RTC’s cofactors, we solved high-resolution crystal structures of Nsp7 bound to Nsp8. Structures were determined in three different crystal forms (Nsp7/8A, Nsp7/8B, and Nsp7/8C) and deposited in the PDB (PDB: 6WIQ, 6WQD, and 6XIP, Table S1) early in the pandemic. Our deposited structures are similar to that reported later by Konkolova et al. (15). Crystals of Nsp7/8A were shown to have a truncated N-terminus (at residue Glu78) for Nsp8. The other structures were obtained from crystallization in the presence of protease Glu-C (V8). Below, the description of the Nsp7/8 structure is based on the 1.5 Å structure Nsp7/8C, unless otherwise mentioned.

The structures of Nsp7 and Nsp8 have strong similarities to the same proteins and variants from other coronaviruses. Nsp7 (Fig. 1 *A*) has three consecutive *α*-helices (*α*1, *α*2, and *α*3) forming a three-helical coiled-coil bundle and the C-terminal loop (residues 62–70) that in some structures is followed by a short, not well-defined helix (residues 68–72). The conformation of Nsp8 has previously been described to resemble a golf club (Fig. 1 *A*; (9)). It has an N-terminal *α*-helix (*α*1) that starts in our structure at Asp78 because of the truncation by Glu-C, which is highly positively charged for binding RNA. This helix is followed by the second *α*-helix (*α*2), which connects through a long loop to a half *β*-barrel-like domain formed by five antiparallel *β* strands (*β*1–5) with a small *α*-helix (*α*3) inserted between the first and second strands and another insertion of a long loop that contains two half-turn helices (labeled *α*4). The C-terminus (193–198) of the Nsp8 is well defined in Nsp7/8B, but not in other forms.

In all of our crystal forms, the heterodimer formed between Nsp7 and 8 places Nsp7 near the half *β*-barrel-like domain of Nsp8 with the N-terminal helix of Nsp8 extended and pointing away (approximate dimensions of 40 × 40 × 46 Å) (Fig. 1 *A*). The similarity between all heterodimers in all our crystal forms is remarkable, with a root mean square deviation of 0.82 Å for 184 aligned C*α* atoms despite different unit cell dimensions, different space groups, and different asymmetric units. These heterodimers are also similar to those from other coronaviruses, for example, RMSD between the heterodimer of Nsp7/8C and that of the Nsp7/8 from SARS-CoV (PDB: 5F22) is 0.91 Å for 184 C*α* atoms. These similarities suggest that, once formed, the heterodimer is rigid.

The interface between Nsp7 and Nsp8 is made through hydrophobic contacts between six bundled helices. The helices involved are the two N-terminal helices (*α*1 and *α*2) from Nsp8 and four helices from Nsp7. At the C-terminus, the fourth helix in Nsp7 is not well defined, and the loop of residues 66–72 wedges in between the two (*α*1 and *α*2) Nsp8 helices to extend the interacting surface. The dimer interface area is 2834 Å^2^ with 72% hydrophobic contacts. The key interfacial residues are shown in Fig. 1 *C*.

Our heterodimer structure is in good agreement with the recent cryo-EM structures of Nsp7 and Nsp8 bound to Nsp12 with and without RNA. Using the most complete structure (PDB: 6YYT) from Hillen et al. (10), the superposition has an RMSD of 1.0 Å. There are two Nsp8s in this structure, but only one is in contact with Nsp7. The interactions between Nsp12 and the Nsp7/8 are mostly through the Nsp7 and the region known as the thumb domain (residues 812–932) located at the C-terminus of Nsp12. The Nsp8 monomer interacts with the fingers domain (residues 250–398) of Nsp12 primarily through *α*2, the following loop, and *β*1 (residues 99–126) and has a significantly different conformation compared to the Nsp7/8 heterodimer. The C-terminal two-thirds of Nsp8 (residues 127–192) is quite similar (overall difference of 1.2 Å over 67 residues). Finally, the location and orientation of the N-terminal helices are different from those in the Nsp7/8 heterodimer. In the cryo-EM structures, the N-terminal helix of Nsp8 makes significant contacts with Nsp12, including the Glu81 region, which perhaps protects Nsp8 from the proteolysis we observed during our crystallographic efforts.

In contrast to previously reported Nsp7/8 structures from other coronaviruses, in all three of our structures, either as in the asymmetric content or symmetry related, a heterotetramer or a dimer of heterodimers is present as an elongated, thick rod shape of 40 × 92 Å (Fig. 1 *B*). The differing crystal forms show only slight variation in the packing of Nsp7/8 heterodimers to form heterotetramers. Comparing the heterotetramers in the asymmetric units, the RMSD is 1.28 Å for 368 aligned C*α* atoms. The heterotetramers are formed between the two heterodimers that are related by the noncrystallographic twofold rotational symmetry. In Nsp7/8A, the tetramers are formed by the crystallographic twofold rotational symmetry.

The heterotetramer interface involves a total of six helices: *α*1 and *α*2 of Nsp7 and their symmetry mates (twofold rotation) *α*1′ and *α*2′ and *α*1 of Nsp8 and the symmetry mate *α*1′. The symmetry-related secondary structures are indicated by apostrophes. The tetramer interface is not as extensive as that of the dimer having 1813 Å^2^ with 73% being hydrophobic. The residues involved are mostly making hydrophobic contacts with symmetrically related residues. Importantly, a symmetrically related interchain disulfide bond is also found between Cys8 of Nsp7 and its symmetry mate (Fig. 1 *D*).

Among the two crystal structures of Nsp7/8 complexes from SARS-CoV reported, the tetrameric form (PDB: 5F22) is similar to our tetrameric structures. The second form (PDB: 2AHM) of the hexadecameric superstructure of eight each of Nsp7/8 presents a cylindrical ring ~90 Å long and with ~30 Å inner diameter lined with positive charge and could accommodate two and a half turns of RNA duplex (9), suggesting a role as a primase for Nsp12. In the hexadecamer, there are two different heterodimers with two different conformations of Nsp8. The tetramer interface is angularly shifted to propagate a ring-like rather than linear topology. In our tetrameric form, each heterodimer is in near identical conformations.

### Assemblies in solution by SAXS and SANS

We demonstrated we could successfully produce an active RTC complex from Nsp7/8/12 using a radioactive-based label extension assay. The extension assay showed the RTC creating duplexed 36 basepaired RNA from a 31 base hairpin substrate where the 5′ overhang had five unpaired bases (Fig. S1). Confident in the relevance of our largest complex, we sought to characterize all potential subcomplexes.

To inform on assembly and conformation in solution, we collected SEC-MALS-SAXS, SANS, and other biochemical data on mixtures of Nsp7, Nsp8, Nsp12, and RNA (Fig. 2). Before experiments, all samples were extensively purified and prepared with the sequences as described in Materials and methods. We used two primary RNA substrates. Our dsRNA has an 8 base overhang and 28 bases of duplex (Fig. S3). Uridine comprises the first four bases of the 5′ overhang. The sequence is analogous to that used in a cryo-EM study (10) differing only in that the strands are continuous rather than containing breaks. The 36-base ssRNA is derived from a long chain of dsRNA and is referred to as long ssRNA.

Each SEC-MALS-SAXS elution contains considerable information (e.g., Fig. 3 *A*). Analysis of the MALS on elution traces (*left axis*, *solid* and *dashed lines*) is used to determine the molecular mass (*right axis*, *circles*). Analyzed SAXS profiles are generated by integrating in regions of constant mass and Rg from SEC-MALS-SAXS (e.g., Fig. 3 *B*) across the main peaks in an elution. Calculations performed on an atomic model generate a SAXS curve (using FOXS (40,41) that can be compared to the experiment using *χ*^2^ or residual as metric of agreement. If agreement is poor (e.g., Fig. 3 *B*, *lower plots*), then the models are adjusted by molecular dynamics in BILBOMD (39) until an adequate fit and residual are attained. In cases of conformational flexibility, a single model will not be sufficient to fit the data, and an ensemble of models must be used (e.g., Fig. 3, *C*–*E*). All scattering measurements were made in slightly reducing conditions, mimicking intracellular space, to prevent disulfide bond formation. Using the isotope-based masking properties of SANS, proteins were also examined for conformational change when interacting with masked components in complexes (e.g., Fig. 3 *F*). Global parameters extracted from these SAXS and SANS profiles can be found in Table S2. Below, we organize our results in order of increasing mixture complexity.

### Nsp7 forms multimers in solution and does not bind RNA

The SEC-MALS-SAXS of Nsp7 reveals that Nsp7 forms multimers with a different organization than that found in our crystal structure of Nsp7/8 (Figs. 2 and 3). From the dominant late peak, MALS-SAXS measurements are consistent with a well-folded dimer with a mass of 17 kDa (monomeric mass 9 kDa) and a maximal dimension (Dmax) of 60 Å (Fig. S2 *B*). The primary peak in the MALS-based chromatogram is preceded with larger multimer of Nsp7 (Fig. 3 *A*). The presence of larger multimers of Nsp7 are important to note because they complicate the analysis of complexes of Nsp7 with Nsp8, Nsp12, and RNA described further. The Nsp7 multimers can erroneously lead to an interpretation that Nsp7 is part of larger complexes, as homo-oligomers of Nsp7 coelute with larger macromolecular complexes.

Indicative of a different assembly in solution, SAXS calculated from the Nsp7 dimer taken from the Nsp7/8C crystal structure disagreed with the measured SAXS data (*χ*^2^ = 40). Poor agreement remained (*χ*^2^ = 7.5, Fig. 3 *B*) even after optimized remodeling of the C-terminal helix (69–84) that is solvent exposed in the absence of Nsp8. Furthermore, the shape calculated from our SAXS data by DAMMIF (50) is more elongated than the dimer taken from the Nsp7/8 structure (Fig. 3 *C*). Assuming the Nsp7 monomer fold visualized in our crystal structure, orientations of Nsp7 in the dimer that fitted the SAXS data were found using the SAXS guided computational docking program FOXDOCK (42). The best docking results were from head-to-tail orientations of Nsp7 monomers (*χ*^2^ = 1.6) (Fig. 3 *C*). The head-to-tail interface may also allow the formation of the larger oligomers observed in MALS chromatograms (Fig. S2 *A*). These oligomers were further analyzed and were consistent with an elongated, rather than globular, form of Nsp7 dimers (Fig. S2 *B*).

Nsp7 did not complex with RNA by SEC-MALS-SAXS analysis. Measurements of mixtures of Nsp7 with RNA resulted in SAXS and masses consistent with the two eluting separately (Fig. S2 *A*). These results were supported by comparisons to measurements on the RNA run separately (Fig. S3, A and *B*).

### Nsp8 forms concentration-dependent multimers and has an alternate conformation when monomeric

Nsp8 forms concentration-dependent oligomers in solution. SAXS profiles collected in high-throughput mode at three concentrations (2.5, 5, 10 mg/mL) varied more significantly than could be explained by a concentration-dependent long-range attraction, indicating complexation between monomeric units (Fig. S4 *A*). Similarly, SEC-MALS-SAXS from Nsp8 showed an asymmetric elution peak, with the front of the peak having a larger molecular mass than the later-eluting material that matched the monomeric mass of Nsp8 (Fig. 3 *A*).

As a monomer, Nsp8 adopts a different conformation to that observed in atomically resolved complexes. The conformations of Nsp8 monomers found in the Nsp7/8 crystal structure (PDB: 3UB0 (49) and Nsp7/8/12 cryo-EM structure (PDB: 6YYT (10) did not match the SEC-SAXS data from the elution region containing the monomers (*χ*^2^ = 40, Fig. 3 *B*). In these Nsp7/8 or Nsp7/8/12/RNA complex structures, the Nsp8 N-terminal helix-turn-helix bundle (1–100) adopts various extended conformations. To find a conformation that fits the SAXS data, we employed conformational sampling of the N-terminal helix bundle. The flexible tethers between the head and two distinct helix regions (1–82 and 86–100) were identified by structural comparison of the two Nsp8 conformers taken from the Nsp7/8/12/RNA complex (Fig. 3 *D*; (10)). Despite providing a nearly exhaustive search of extended and compact conformations to select an ensemble, an excellent fit to the SAXS data (Fig. 3 *B*) required a compact configuration with the head domain in close proximity to the N-terminal region of the helix-turn-helix region (Figs. 3 *D* and S4). The folding back of the structure may be driven by a polar interaction between a positively charged segment of the long connecting helix with a negatively charged portion of the beta-barrel containing domain (head), as calculated by PDB2PQR/Adaptive Poisson-Boltzmann Solver (Fig. S5; (51,52)). These regions could be further driving the multimerization, as shown in the crystal structure of the Nsp7/8 complex of feline coronavirus (PDB: 3UB0) (Fig. S4 *C*; (49)).

To fit higher concentrations of Nsp8, we created a model for dimers that combines our monomeric model with dimerization contacts of Nsp8 found in an analog crystal structure (49). A mixture of the dimer and monomer models fitted the SAXS data for Nsp8 at a 2.5 mg/mL concentration (Fig. S4 *A*) and the SAXS data from the leading edge of the SEC major elution peak. At our higher concentrations (5 and 10 mg/mL), the SAXS profiles could not be fitted using a monomer-dimer mixture and required a model with longer maximal distances, as shown by Dmax values determined from the P(r) function (Fig. S4 *B*). A small contribution of a larger tetrameric state built based on the Nsp7/8 structure (PDB: 2AHM) (9) was necessary to fit SAXS data for the two highest protein concentrations (Fig. S4 *C*).

### Nsp12

Nsp12 alone expresses poorly and has low solubility. This suggests that Nsp12 is unlikely to function alone and must form heteromeric complexes for stability. The maximal concentrations obtained were 2.5 mg/mL, above which aggregation of the protein was evident. The MALS and SAXS mass is consistent with a monomer (Figure 2, Figure 3 *A*). The interpretable SAXS signals below q < 0.2 Å^−1^ were in good agreement (*χ*^2^ = 1.4, Fig. 3 *B*) with the available atomic model of Nsp12 (Fig. 3 *E*; (10)). The Rg of the measured profile was 31.1 ± 0.5 Å, whereas that calculated from the model with added missing N-terminal region (1–31, 50–77) is 31.6 Å (Table S2).

### Nsp8 binds RNA, but not DNA

We characterized the interaction of Nsp8 with dsRNA (Figs. 4 and S3) and dsDNA (Fig. S5). The dsDNA is analogous in sequence to dsRNA. Nsp8 did not form a complex with dsDNA because both the Nsp8 and dsDNA elute separately at elution times consistent with each run independently. In contrast, Nsp8 forms two types of complexes with the dsRNA, contributing to two well-separated peaks (Fig. 4 *A*). The first elution peak (13.5 min after injection) is consistent with a 1:1 Nsp8 dsRNA complex, whereas in the second Nsp8/dsRNA peak (14.3 min), the mass decreases rapidly across the elution profile, indicating the presence of a heterogeneous mixture of Nsp8 and free dsRNA.

We further tested whether Nsp8 binding to DNA or RNA is length or sequence dependent. Nsp8 did not bind either long (36 base) or short (28 base) ssDNA in our SEC-MALS-SAXS analysis or in an electrophoretic mobility shift assay (EMSA) (Fig. 4 *D*). Nsp8 did bind the long ssRNA, but not the short ssRNA, at high affinity. The mixture of Nsp8 with long ssRNA shows only one distinct peak, consistent with the 1:1 complex as judged by determining the molecular mass and SAXS parameters (Figure 2, Figure 4 *A*). We further tested binding of long ssRNA, short ssRNA, and the long ssRNA without the poly-U 5′ end by EMSA. Nsp8 showed evidence of binding all three; however, the affinities are difficult to assess with EMSA bands. The EMSA bands with ssRNA are split. On the same mixtures from SEC-MALS-SAXS, the mass through the peak drops rapidly (Fig. 4 *A*) suggestive that multiple Nsp8s could bind the same ssRNA. These results demonstrate Nsp8 discriminates between RNA and DNA. SEC-MALS-SAXS showed a preference for longer ssRNA over the shorter.

We found Nsp8/dsRNA models that fit the SAXS data from the highest quality signal in the constant mass region of the main elution peak (Fig. 4, *A* and *B*). This model is supported from the elongated shape determined from SAXS by DAMMIF (50) and consists of Nsp8 binding dsRNA along their long axes (Fig. 4 *C*). In our rigid-body modeling using BILBOMD (39), we preserved the Nsp8-dsRNA interaction at the N-terminal Nsp8 helix bundle that contains positively charge patches (Fig. S5) and sampled conformations of C-terminal head domain. An excellent fit (*χ*^2^ = 1.7, Fig. 4 *B*) was found with the Nsp8 head domain wrapping around the RNA capping the terminal region of the RNA molecule (Fig. 4 *C*), providing a structural model for Nsp8 RNA binding.

### The Nsp7/8 complex is transient in solution

The shape of the elution peak from coexpressed and affinity-tag-purified Nsp7/8 is consistent with a transient complex composed of oligomers and free proteins. The SEC-MALS-SAXS elution is dominated by an asymmetric peak with a long tailing shoulder region (Fig. 4 *A*, *lower*). The mass at the leading edge by MALS is 45 kDa, and SAXS is ~42 kDa, which is smaller than the tetramer’s expected molecular mass (63 kDa) (Fig. 2). Furthermore, a well-separated peak observed at later elution times is consistent with that observed from Nsp7 dimers, further supporting a transient Nsp7/8 complex (Fig. 4 *A*).

To fit the SAXS data from the dominant peak, we used an ensemble of models. The ensemble pool included a dimer and heterotetramer of Nsp7/8 built from our crystal structure in which Nsp8 is in an extended conformation to include a helix and N-terminal bundle. We also included a monomeric Nsp8 with various conformations of the N-terminal helix bundle and an Nsp8 dimer with open and compact head domain (see Fig. S4 *C*). The data are the best fit by a mixture of 26% Nsp7/8 heterotetramer, 56% monomeric Nsp8, and 18% Nsp8 dimer with a compact head domain (*χ*^2^ = 2.2, Fig. 4, *B* and *E*). The Nsp7/8 heterotetramer observed in our crystal structures is a minor population at these concentrations.

A similar analysis was performed on SANS data collected from the same complex differing by collection at high concentration and in a fully equilibrated sample (non-SEC). The mass determined from SANS was also slightly lower (56 ± 3 kDa) than the Nsp7/8 heterotetramer. The Nsp7/8 heterotetramer crystal structure did not fit the SAXS data. However, using OLIGOMER, a good fit (*χ*^2^ < 1.0) was obtained with a mixture of 88% Nsp7/8 heterotetramer and 12% Nsp8 monomer. This supports the SEC-SAXS analysis that shows the dynamic and concentration-dependent nature of the interaction between Nsp7 and Nsp8.

Furthermore, to test our findings that Nsp7 induces structural change in Nsp8 relative to its monomeric state, we performed contrast-matching SANS experiments with the Nsp7/8 complex (Fig. 3 *F*). Deuterated Nsp7 (dNsp7) was copurified with protiated Nsp8 to form dNsp7-Nsp8. Using the contrast match point of dNsp7 (90% D_2_O), we single out the scattering from complexed Nsp8. Fig. 3 *F* compares the P(r) of dNsp7-Nsp8 against a solution of Nsp8 without Nsp7. The P(r) from the complex dNsp7-Nsp8 has a bimodal shape, indicating two well-separated protein densities, whereas the P(r) of Nsp8 alone is more centralized to one main distance. The change verifies Nsp7 dramatically modifies Nsp8 conformation or assembly.

### RNA competes with Nsp7 to interact with Nsp8

In contradiction to several proposed pathways, our SEC-MALS-SAXS experiments do not support a stable ternary Nsp7/8 dsRNA complex. Quite the opposite; our results indicate a competition between Nsp7 and dsRNA for Nsp8 binding. The elution peak from mixtures of Nsp7/8 with RNA is shifted in agreement with the smaller measured mass (MALS) from 45 kDa for Nsp7/8 to ~35 kDa for Nsp7/8 + dsRNA (Fig. 4 *A*, *lower*). The secondary SEC peak for free Nsp7 is more pronounced and well separated (Fig. 4 *A*), suggesting the uncoupling of Nsp7 from the complex.

Fitting of the SAXS data provides further evidence that dsRNA destabilized the Nsp7/8 interface. From the pool of models that includes conformers of Nsp8, Nsp7 monomer and dimer, Nsp7/8 dimer and tetramer, and multiple conformers of Nsp8-dsRNA, the best fit was obtained with the Nsp8-dsRNA complex (*χ*^2^ = 1.7, Fig. 4 *B*) described above. The multistate model selection that contains Nsp7 dimer further improved the SAXS fit (*χ*^2^ = 1.6, Fig. 3, *B* and *D*) and suggested transient binding of dsRNA that led to the decoupling of Nsp7/8 and presence of free Nsp7 across the peak. The presence of Nsp7 dimers also explains the smaller Rg and mass determined by SAXS or MALS relatively to the values measured for the Nsp8/dsRNA complex (Rg 28.9 Å vs. 32.2 Å, Mass_MALS_ 32 vs. 45 kDa, and Mass_SAXS_ 30 vs. 45 kDa) (Figure 2, Figure 4 *A*; Table S2).

Contrast-matching SANS experiments, masking the RNA signal in 65% D_2_O, were also performed on Nsp7/8 dsRNA mixtures. The contrast-matched SANS profile is best fitted (*χ*^2^ = 3.0) with the Nsp8 monomer (Fig. 4 *G*), supporting the dissociation of the Nsp7/8 complex observed in the SEC-SAXS. Altogether, the combined SANS and SAXS indicate that RNA alters the interactions found in our crystal structure of Nsp7/8 alone, leading to the formation of a smaller Nsp8-dsRNA complex and disassociation of Nsp7 from the complex (Fig. 4 *F*).

### Without Nsp7, Nsp12 recruits one Nsp8 to its finger region and does not bind RNA

The mass across the center of the SEC-MALS-SAXS main peak from coexpressed Nsp8 and 12 is 130 kDa, in agreement with the mass of an Nsp8/12 1:1 complex (Fig. 5 *A*). The main peak trails into a mass (110 kDa) consistent with a free Nsp12. The broadening of the elution peak indicates disassociation of Nsp8 from Nsp12. The central portion of the main peak was merged to obtain a SAXS profile sufficient to be modeled in detail.

The Nsp8/12 SAXS profile was fitted with a pool of the models containing various conformers of Nsp8 (Fig. 3 *D*), Nsp12 (Fig. 3 *E*), and three models of the Nsp8/12 complex with Nsp8 located at the fingers, thumb region, or both (10). The pool also contained alternative models of the Nsp8/12 complex with an extended Nsp8-helix bundle region. The best fit (*χ*^2^ = 1.4) was obtained with a single model of Nsp8/12 containing one Nsp8 bound to the finger region (Fig. 5 *D*). This model matches experimental SAXS better than the Nsp8/12 model with both Nsp8 bound (*χ*^2^ = 24.6) (Fig. 5 *B*) and agrees with the determined mass (Fig. 2). We attempted to improve the fit with the multistate models, but the single conformation remained the best fit.

These findings suggest Nsp12 recruits only one Nsp8 to the finger region and raises the question of whether Nsp7, RNA, or both are required to stabilize the Nsp8 interaction at the thumb region. Therefore, we investigated Nsp7/8/12 assembly in solution and the interactions of Nsp8/12 with RNAs.

Nsp7, Nsp8, and Nsp12 were coexpressed and purified for SEC-MALS-SAXS analysis. The first SEC trace, before SAXS measurements, shows a split peak and tail with a significant peak from Nsp7 at later elution times, already suggesting transient dissociation of Nsp7 from the Nsp8/12 complex (Fig. S7). The early fractions were subsequently analyzed (and consequently purified by SEC a second time) by SEC-MALS-SAXS. The mass of the first elution peak (132 kDa) agrees with the Nsp8/12 1:1 complex. The elution time of the peak (Fig. 5 *A*), determined mass, SAXS parameters (Fig. 2), and calculated P(r) functions (Fig. 5 *C*) from this first peak are identical to those reported above from Nsp8/12 when no Nsp7 was present. Furthermore, the Nsp8/12 1:1 model (Fig. 4 *D*) gives an excellent match to the experimental SAXS curve (*χ*^2^ = 2.2) (Fig. 5 *B*) that is distinct from other potential models, including an Nsp7/8/12 1:2:1 complex with a significantly worse fit (*χ*^2^ = 19.9). The trailing shoulder and peak from the elution trace is consistent with Nsp12 alone and an Nsp7 dimer (Fig. 5 *A*), further supporting the dissociation of Nsp7 from the Nsp8/12 1:1 complex.

Mixing an excess of RNA with Nsp8/12 does not lead to a high-affinity complex. Identical elution time and MALS-measured molecular mass (~130 kDa) of the early elution peak of Nsp8/12 + dsRNA (Fig. 5 *A*), Nsp8/12 + ssRNA (Fig. S7), and Nsp8/12 alone clearly show the absence of an Nsp8/12 interaction with RNA. The low or nonexistent binding of RNA by Nsp8/12 is surprising because Nsp8 alone binds RNA strongly. We find that Nsp8/12 does not stably interact with Nsp7 or RNA on its own; rather, Nsp7 needs to interact with Nsp8 to form the complete SARS-Cov-2 polymerase machinery.

### Nsp7/8/12 requires RNA for stability

Adding dsRNA or ssRNA stabilizes the Nsp7/8/12/RNA within a 1:2:1:1 complex as visualized in cryo-EM atomic-resolution structures (10). Thoroughly mixing near equivalent molar ratios of dsRNA or ssRNA with the initially purified Nsp7/8/12 complex, assuming a 1:2:1 complex, yields a sharp and near symmetric SEC elution peak (Figs. 5 *A* and S6). The shift in the SEC peak and determined mass (~190 kDa) suggests the formation of Nsp7/8/12/RNA in 1:2:1:1 complex for both complexes, which agrees with the theoretical mass of 1:2:1:1 complexes with 181 kDa for Nsp7/8/12/ssRNA and 172 kDa Nsp7/8/12/dsRNA (Fig. 2). The experimental SAXS profiles (Fig. 5 *B*) for both complexes are consistent with models built from the cryo-EM structure (10) by adding all missing regions in the protein and RNA region and removing a shorter RNA strand for Nsp7/8/12/ssRNA complex (Fig. 5 *D*). This agreement further suggests that both Nsp8 copies remain pointed in the direction of RNA polymerization rather than flexing in alternate conformations.

## Discussion

Our overall aim was to further develop a comprehensive understanding of the SARS-CoV-2 RTC and identify strategies for inhibiting its assembly and mechanism. To this end, we have integrated information from available cryo-EM and x-ray crystallography structures with the results reported above, which include biochemical assays, atomic-resolution structures of Nsp7/8, and 20 SAXS and SANS experiments on components of the RTC. Our crystal structures, those resolved by others, and cryo-EM results provide a basis to interpret our solution scattering studies that probe the transient and plastic properties of the assembly, which are difficult to attain with static structures alone. The highly evolved and dynamic nature of this essential complex greatly enables SARS-CoV-2; however, these same properties also provide many avenues for disruption and inhibition. Summarizing our results, we have arrived at the following insights.

At high concentrations, as found in our crystal structure, Nsp7/8 forms a tetrameric structure composed of a dimer of heterodimers. However, this structure is transient, and at low concentrations, the proteins disassociate into mixtures of the individual components and smaller complexes. Nsp7 primarily exists as a dimer, but longer oligomeric structures are also present, which can be confounding to size-based purification methods because Nsp7 will be present in one oligomeric form or another at separate elution times. Without being bound to Nsp7, Nsp8 adopts a compact conformation not yet observed in crystal structures and will oligomerize into flat aggregates at high concentrations. Nsp8 will readily bind dsRNA and ssRNA, but not ssDNA. Nsp12 alone is unstable and prone to aggregation even at low concentrations (>2.5 mg/mL). Binding of Nsp8 markedly improves stability.

The atomic coordinates from our crystal structure and our solution scattering results inform on the consequences of Nsp7’s overall hydrophobic character. The eight hydrophobic amino acids make up 45% of the protein, including 18% leucine and 11% valine. The solvent-excluded interface made between Nsp7 and Nsp8 is 57% hydrophobic. In all of our structural studies, Nsp7 is found in complex with Nsp8 and Nsp12, or it has the propensity to form chains of Nsp7 oligomers on its own. A further consequence of its hydrophobic nature is its ability to disrupt the hydrophobic cores of the other proteins and modify their conformational states as exemplified by its interaction with Nsp8. The 2:2 Nsp7/8 we observe in our crystal structure may be a storage form of the complex when little RNA is available. Nsp7’s ability to compete with RNA, a hydrophilic and charged molecule, for Nsp8 binding is not intuitive. One mechanistic possibility is that the binding of Nsp7 reduces the footprint Nsp8 has to bind RNA and serves to offload RNA from Nsp8. This mechanism could be fundamental to the operation or assembly of the RTC and provides a rational for expressing Nsp7 as a separate peptide rather than as part of Nsp8 or Nsp12.

We observed two stabilized structures with Nsp12. In the absence of its binding partners, Nsp12 alone seems inherently unstable, which limited our ability to create complexes by mixing the purified proteins. However, by coexpressing the individual proteins, reasonable yields of the complexes could be attained. A stable 1:1 structure forms between Nsp8 and Nsp12, where an Nsp8 sits on the fingers of Nsp12. This complex, however, is not capable of binding the RNAs we tested. Although cryo-EM has partially visualized an Nsp7/8/12 structure without RNA, the sample had to be supplemented with significant concentrations of Nsp7 and 8 for resolving these structures. In our hands and at the concentrations we made our measurements (1–5 mg/mL), Nsp7/8/12 does not form a stable structure until RNA is present. Once RNA is present, a very stable 1:2:1:1 Nsp7/8/12/RNA complex is formed both with dsRNA and ssRNA.

In all the most complete cryo-EM structures of the Nsp7/8/12/RNA with and without Nsp13 or Nsp9, the structure and position of Nsp7 and 8 are remarkably similar. This similarity suggests Nsp7 and 8 are static components during replication. Their role is thought to be threefold: to help close the complex once RNA is bound, to guide RNA upon exit, and to stabilize contacts with Nsp13 and other proteins. As noted with the first complete structure, Nsp7 and 8 confer processivity (10). The orientations of the helical extensions on Nsp8 remain uniquely observed in the RTC and may function similar to sliding clamps in DNA replication. Nsp8’s structure is suited to preforming or further enhancing a straightened RNA with positively charged RNA binding patches spaced to coincide with an RNA double helix.

However, for these roles, expression of Nsp7 and Nsp8 as independent polypeptides, rather than as one protein or as part of Nsp12, seems inefficient. Nsp7 and Nsp8 have been postulated to take part in other viral activities, which may justify their independence. Exploring other potential avenues, we build upon cryo-EM and our results to suggest more dynamic roles for the components. We propose that Nsp8 is involved in recognizing ssRNA over ssDNA and guiding ssRNA to or from the RTC complex. Our observation that Nsp8 adopts a different conformation alone relative to bound states suggests Nsp8 is a dynamic component of the macromolecular machine. Based on the exclusive binding of either Nsp7 or RNA by Nsp8, but not both simultaneously, we propose that Nsp7 promotes the release of RNA from Nsp8 once it has been guided to the RTC by disrupting Nsp8’s head domain. The stability of the Nsp8 on the fingers domain is intriguing and certainly serves to further stabilize the RTC’s RNA binding. However, we have shown that the fingers-bound Nsp8 is not sufficient to bind RNA and Nsp7 is necessary.

The binding and position of two Nsp8s could play a role in transitions that occur during RNA transcription. RNA rotates and progresses during polymerization. The compacting and expanding properties of Nsp8 could be part of a retraction mechanism in which, once the N-terminus of Nsp8 is overstretched, it releases and rebinds closer to Nsp12, where newly synthesized and duplexed RNA is emerging. Alternatively, the combination of RNA rotation and progression may pull the fingers-bound Nsp8 off of Nsp12, and its vacant spot may be taken up by the Nsp8 bound to the thumb (Fig. 6). The energetics could be favorable because there is a 1:1 trade from an Nsp8 in a slightly destabilized configuration through rotation and progression of the RNA. The now-vacant spot on the thumb domain would then be occupied by free Nsp8, or perhaps both Nsp7 and 8 are replaced. The transition would also allow Nsp12 to reset or release its grasp on RNA because binding of RNA by Nsp12 is dependent on Nsp7. The available structures of Nsp7/8/12/13/RNA do not prohibit such an exchange and rather suggest that Nsp13 could also be part of the transition, with the template strand eventually released by the Nsp13 helicase and grabbed by a new one bound at the thumb as well.

The possible mechanistic roles of the components in the RTC described above (Fig. 6), require significant further investigation. In addition to the continued clarity provided by high-resolution cryo-EM and crystallographic structures, further solution- and cell-based studies to examine the dynamics of RTC in its many contexts will be helpful. For example, RNA transcription and polymerization appear to occur in double-membrane vesicles, where the concentrations of the components may be manipulated. We observed several concentration-dependent assemblies in our studies. Additionally, this study was conducted with a limited and specific set of RNAs that may bias our findings. Further studies with a variety of RNA substrates are necessary.

## Conclusions

In our study, we systematically examined components and complexes relevant to the RTC of SARS-CoV-2. We were able to crystalize Nsp7/8, which provides us atomic-resolution details on the interactions between the two components. We have uncovered conformations and identified stable complexes and, to some extent most interestingly, transient complexes. Our results are relevant for a comprehensive understanding of the RTC mechanism and suggest strategies for the disruption of assembly. Developing a further mechanistic understanding will provide insight into how current drugs inhibit the RTC and other potential ways to interfere with the complex.

## Author contributions

G.L.H. wrote the draft, organized experiments, analyzed structures, and is a corresponding author. M.H. organized experiments, analyzed SAXS and MALS data, analyzed structures, prepared SAXS figures and helped write the manuscript. D.J.R. collected SEC-MALS-SAXS data. J.C.B. helped prepare SAXS samples and checked on reduction state of buffers. S.E.T. helped organize experiments and write the manuscript. A.H.S. performed RNA binding assays. J.W. performed RNA extension assay. M.W. purified and crystallized proteins and determined and refined crystal structures. R.J. cloned genes, expressed and coexpressed proteins, and purified complexes. Y.K. collected data and solved and refined structures. A.J. initiated the project and coordinated efforts between sites. W.L. performed SANS experiments and data analysis and helped to write the manuscript. Q.Z. developed expression and purification protocols for deuterated complexes. K.L.W. developed deuteration protocol for individual proteins for SANS. Y.F. expressed and purified protiated Nsp7/8 complex. S.V.P. supported SANS experiments. H.M.O. led the ORNL effort, performed sample preparation for SANS experiments and data analysis, and helped to write the manuscript.

## Supporting material

Document S1. Supporting materials and methods, Figs. S1–S7, and Tables S1 and S2

Document S2. Article plus supporting material
