# Supplementary material for: Transient and stabilized complexes of Nsp7, Nsp8, and Nsp12 in SARS-CoV-2 replication
Source: Biophys J. 2021 Jun 29;120(15):3152–65. doi: 10.1016/j.bpj.2021.06.006 (PMC8238635; doi:10.1016/j.bpj.2021.06.006)
Supplement: Document S1. Supporting materials and methods, Figs. S1–S7, and Tables S1 and S2 [file mmc1.pdf]

Biophysical Journal, Volume *120*

Supplemental information

Transient and stabilized complexes of Nsp7, Nsp8, and Nsp12 in SARS-CoV-2 replication

Mateusz Wilamowski, Michal Hammel, Wellington Leite, Qiu Zhang, Youngchang Kim, Kevin L. Weiss, Robert Jedrzejczak, Daniel J. Rosenberg, Yichong Fan, Jacek Wower, Jan C. Bierma, Altaf H. Sarker, Susan E. Tsutakawa, Sai Venkatesh Pingali, Hugh M. O'Neill, Andrzej Joachimiak, and Greg L. Hura

Supporting Material for "Transient and stabilized complexes of Nsp7, Nsp8 and Nsp12 in SARS-CoV-2 replication

Authors: Mateusz Wilamowski*, Michal Hammel*, Wellington Leite*, Qiu Zhang, Youngchang Kim, Kevin Weiss, Robert Jedrzejczak, Daniel J. Rosenberg, Yichong Fan, Jacek Wower, Jan Bierma, Altaf H. Sarker, Susan E. Tsutakawa, Sai Venkatesh Pingali, Hugh M. O'Neill[#], Andrzej Joachimiak[#], Greg L. Hura[#]

***contributed equally [#]corresponding authors**

Correspondence should be addressed to:

glhura@lbl.gov, oneillhm@ornl.gov, andrzejj@anl.gov

Supporting Material Materials and Methods

Macromolecular sequences, expression and purification prior to structural studies

Protein Sequences: NCBI reference sequences for Nsp7, Nsp8 and Nsp12 are YP_009725303.1, YP_009725304.1, and YP_009725307.1 respectively and are further available in the supplementary information.

Protein Sequences:

Nsp7:SKMSDVKCTSVVLLSVLQQLRVESSSKLWAQCVQLHNDILLAKDTTEAFEKMVSLLSVLLSMQGAVDINKLCEE
MLDNRATLQ

Nsp8:AIASEFSSLPSYAAFATAQEAYEQAVANGDSEVVLKKLKKSLNVAKSEFDRDAAMQRKLEKMADQAMTQMYK
QARSEDKRAKVTSAMQTMLFTMLRKLDNDALNNIINNARDGCVPLNIIPLTTAAKLMVVIPDYNTYKNTCDGTTFTYAS
ALWEIQQVVDADSKIVQLSEISMDNSPNLAWPLIVTALRANSAVKLQ

Nsp12:SADAQSFLNRVCGVSAARLTPCGTGTSTDVVYRAFDIYNDKVAGFAKFLKTNCCRFQEKDEDDNLIDSYFVVKR
HTFSNYQHEETIYNLLKDCPAVAKHDFFKFRIDGDMVPHISRQRLTKYTMADLVYALRHFDEGNCDTLKEILVTYNCCD
DDYFNKKDWYDFVENPDILRVYANLGERVRQALLKTVQFCDAMRNAGIVGVLTLDNQDLNGNWYDFGDFIQTTPGS
GVPVVDSYYSLLMPILTLTRALTAESHVDTDLTKPYIKWDLLKYDFTEERLKLFDRYFKYWDQTYHPNCVNCLDDRCILHC
ANFNVLFSTVFPPTSFGPLVRKIFVDGVPFVVSTGYHFRELGVVHNQDVNLHSSRLSFKELLVYAADPAMHAASGNLLL
DKRTTCFSVAALTNNVAFQTVKPGNFNKDFYDFAVSKGFFKEGSSVELKHFFFAQDGNAAISDYDYYRYNLPTMCDIRQ
LLFVVEVVDKYFDCYDGGCINANQVIVNNLDKSAGFPFNKWGKARLYYDSMSYEDQDALFAYTKRNVIPTITQMNLKY
AISAKNRARTVAGVSICSTMTNRQFHQKLLKSIAATRGATVVIGTSKFYGGWHNMLKTVYSDVENPHLMGWDYPKCD
RAMPNMLRIMASLVLARKHTTCCSLSHRFYRLANECAQVLSEMVMCGGSLYVKPGGTSSGDATTAYANSVFNICQAV
TANVNALLSTDGNKIADKYVRNLQHRLYECLYRNRDVDTDFVNEFYAYLRKHFSMMILSDDAVVCFNSTYASQGLVASI
KNFKSVLYYQNNVFMSEAKCWTETDLTKGPHEFCSQHTMLVKQGDDYVYLPYPDPSRILGAGCFVDDIVKTDGTLMIE
RFVSLAIDAYPLTKHPNQEYADVFHLYLQYIRKLHDELTGHMLDMYSVMLTNDNTSRYWEPEFYEAMYTPHTVLQ

Gene cloning and protein expression:

Genes coding of the Nsp7, Nsp8, and Nsp12 from SARS-CoV-2 (taxid: 2697049) were codon optimized for efficient expression in *E. coli*, and synthetized by Twist Bioscience, San Francisco CA. Cloning of the coding sequences were conducted as reported previously using ligation independent cloning (LIC) method (26). The sequences of Nsp12, and Nsp7 were cloned into the pMCSG53 vector, that possesses an N-terminal His-Tag followed by a Tobacco Etch Mosaic Virus (TEV) protease cleavage site. The coding sequence of

Nsp8 was inserted into pRSF vector to obtain untagged protein, and to pMCSG53 for purification of Nsp8 with His-Tag. The protein expression plasmids were transformed into the E. coli BL21(DE3)-Gold strain (Stratagene, San Diego CA). For purification of Nsp7/8/12, and Nsp8/12 complexes we co-expressed Nsp12 (pMCSG53), and Nsp8 (pRSF) in single cell culture. For purification of Nsp7/8 complex we co-expressed Nsp8 (pRSF), and Nsp7 (pMCSG53). We also expressed Nsp7, Nsp8, and Nsp12 using pMCSG53 vectors containing N-terminal His-Tag. The large-scale expression of the the SARS-CoV-2 proteins were done in LB Lennox medium supplemented with ampicillin 150 µg/ml. The cells were grown at 37°C (190 rpm) until OD_{600nm} was approximately equal to 1. After that, the incubator temperature was changed to 4°C to cool down the bacterial cell suspension. When the culture reached 18°C the medium were supplemented with the following compounds at concentrations 0.2 mM IPTG, 0.1% glucose, 40mM K_2HPO_4. Next, the induced bacteria culture was grown for 20 hours at 16°C with 190 rpm shaking. Subsequently cells were harvested by centrifugation at 7k RCF, and cell pellets were resuspended in lysis buffer comprising 50 mM HEPES pH 8.0, 500 mM NaCl, 5% v/v glycerol, 20 mM imidazole, and 10 mM β-mercaptoethanol.

Deuterium labeled Nsp7, for contrast variation SANS experiments, was expressed using a protocol established by Marley et al. (27). Briefly, LB cultures were grown and monitored until OD_{600nm} reached ~0.7. At that point, the cells were pelleted by centrifugation at 4000×g for 10 minutes in pre-sterilized bottles. Each pellet was then washed in deuterated minimal medium and re-pelleted by centrifugation at 4000×g for 10 minutes. The washed pellets were then resuspended in fresh deuterated minimal medium and transferred to dry, sterile 2.8L Fernbach flasks (250 mL per flask). The cells were then cultivated at 18 °C for 1 hour, induced with 0.2 mM IPTG for ~20 hours, and harvested by centrifugation at 4000×g for 30 minutes. After decanting the supernatant, the protiated and deuterated pellets were stored at -80°C until needed for subsequent protein purification.

Protein purification:

Harvested *E. coli* cell pellets were sonicated on ice at 120W for 5 minutes (4 sec pulses of sonication followed by 20 sec breaks). Cell debris were removed through centrifugation at 30k RCF, 4°C for 1 hour. Supernatants were mixed with 5 ml of Ni^{2+} Sepharose (GE Healthcare Life Sciences, Marlborough MA) previously equilibrated with lysis buffer. Solutions were transferred on chromatography Flex-Column (420400-2510) attached to Vac-Man vacuum system (Promega, Madison WI). After loading of the cell lysate the nickel beads were washed three times with 60 ml of lysis buffer. As next, proteins were eluted using 20 ml of lysis buffer enriched with 500 mM imidazole pH 8.0. Protein concertation's were measured using NanoDrop (Wilmington, DE) spectrophotometer. Subsequently after determination of sample concentration the TEV protease were added in molar ratio 1:30 (TEV : protein). These solutions were incubated for 16 hours at 4°C.

To obtain the Nsp7/8/12 complex we purified Nsp8/12 complex from co-expression system, and as next we added separately purified Nsp7, His-Tags were removed from both Nsp7, and Nsp12 using TEV protease. For purification of Nsp8/12 complex we used co-expression system with a His-Tag on Nsp12. For purification of Nsp7/8 we used a co-expression system with His-Tag on Nsp7. We also purified the Nsp7, Nsp8, and Nsp12 separately using the TEV to remove N-terminal His-Tag attached to the proteins. Subsequently, all purified proteins or obtained complexes were concentrated using 30 kDa cut-off

centrifugal protein concentrators (Merck-Millipore, Burlington, MA). The complex of Nsp7/8/12 was concentrated to 20 mg/ml, the Nsp7/8 was concentrated to 50 mg/ml, and Nsp8, Nsp7, Nsp8/12, Nsp12 were concentrated to approximately 30 mg/ml. Depending on the sample volume, SEC was carried out using a Superdex 200 column in lysis buffer containing 1 mM TCEP instead of β-mercaptoethanol. After SEC gel electrophoresis was done to select the pure fractions that were frozen for further analysis. For purification of Nsp7, Nsp8, Nsp12 the selected fractions after SEC were pooled together and subsequently loaded on Ni^{2+} Sepharose to remove TEV, and contaminating material that binds to the nickel column. Flow-through containing pure protein were concentrated using centrifugal concentrators, and buffer was exchanged with 20 mM HEPES pH 7.5, 150 mM NaCl, 1 mM TCEP, pH 7.5. Protein purity was analyzed and compared with Precision Plus Protein™ Dual Color Standard (BioRad, Hercules, CA) using SDS-PAGE in Tris-glycine buffer and molecular markers. For screening crystallization conditions, we used freshly purified proteins. Proteins were flash cooled at liquid nitrogen for subsequent SAXS and SANS data collection.

Co-purification of deuterated Nsp7 and protiated Nsp8 complex:

The overexpressed deuterated Nsp7 (dNSP7) and protiated Nsp8 *E. coli* cell pastes were resuspended in 50 mL lysis buffer containing 50 mM HEPES, 500 mM NaCl, 20 mM imidazole, 5% glycerol, 1 mM TCEP and EDTA-free protease inhibitor tablet Complete (05056489001, Roche, USA), respectively. The resuspended cells were mixed together and lysed by sonication on ice for 10 min with 50% amplitude using a 4 sec on/10 sec off cycle. The clarified lysate was loaded onto 5 mL HisTrap^{TM} HP column (17524802, Cytiva, Marlborough MA) after centrifugation at 18,000 rpm for 30 min. The column was washed by 10 column volumes (CV) of buffer A (50 mM HEPES, 500 mM NaCl, 10 mM imidazole, 5% glycerol, and 1mM TCEP), 10 CV of 6% buffer B (50 mM HEPES, 500 mM NaCl, 500 mM imidazole, 5% glycerol, and 1 mM TCEP) before elution. The protein was eluted with a 10 CV, 6 to 100% linear gradient. The fractions containing dNsp7 and Nsp8 were confirmed by SDS-PAGE and pooled. The pooled fractions were dialyzed against buffer containing 50 mM HEPES, 300 mM NaCl, 5% glycerol, 1 mM TCEP with TEV protease (1:50 ratio) at 4°C for 16 hours. The cleaved protein was loaded to a 5-mL HisTrap^{TM} HP column to remove TEV protease and other contaminants. Flow-through was collected and concentrated using a centrifugal concentrator with a 30kDa MW cutoff and then loaded to a HiLoad 16/60 Superdex 200 column equilibrated in 50 mM HEPES, 150 mM NaCl, 1 mM TCEP for final. After pooling and concentrating the peak fractions, the purity and concentration of the purified protein were determined by SDS-PAGE and UV-Vis spectrophotometry. Using dialysis, the relative ratio of D_2O/H_2O in the protein solution was adjusted to 90% D_2O. In 90% D2O solvent, the scattering contribution of dNSP7 was negligible because the scattering length density of dNSP7 was matched to that of the solvent.

RNA extension essay

The RNA used in the extension assay folds into a hairpin with a GAAA tetraloop and a single-stranded tail, 5’-GGCUU-3’, at its 5’ terminus (Fig. S1). Its sequence is composed of 31 nucleotides: 5’-pppGGCUUAGGAGAUGAUGAAAGUCAUUCUCCU-OH-3’. We synthesized the 31-mer by *in vitro* transcription as described previously (28). Oligonucleotides used for the construction of the DNA template are:5’-AATTCCTGCAG**TAATACGACTCACTATA**GGCTTAGGAGAATGATGAAAGTC-3’ and 5’-mAmGGAGAATGACTTTCATCATTCT-3’. mA and mG represent the 2’-O-methyl nucleotides used to reduce the proportion of runover transcripts. The underlined portions represent the overlapped segment and bold type indicates the T7 RNA polymerase promoter. To synthesize the internally [^{32}P]-labeled 31-mer

transcripts, 10 ug/ml duplex DNA in a solution containing 200 mM HEPES/KOH (pH7.5), 30 mM $MgCl_2$, 2 mM spermidine, 40 mM DTT, 3 mM each ATP, CTP, GTP and UTP, 0.33 µM [alpha-^{32}P]ATP was incubated with 100 µg/mL T7 RNA polymerase, 5 U/ml inorganic phosphatase (New England BioLabs, Boston MA), SUPERase·In RNase Inhibitor (Invitrogen, Walton MA) in a 50 µL reaction for 5 hours at 37 ^oC. DNA template was removed by addition of 50 U/ml RQ1 RNase-free DNase (Promega, Madison WI) and incubation for 15 min at at 37 ^oC, followed by phenol-chloroform extraction. The 31-mer RNA was recovered from the aqueous phase by ethanol precipitation and then purified by electrophoresis on a 15% denaturing polyacrylamide gel. To remove homodimers and produce a homogeneous solution of hairpins, the gel-purified RNA transcripts dissolved in water were heated to 75 ^oC and gradually cooled to room temperature. The RNA extension reactions contained 10 µM 31-mer RNA hairpin, 5 µM Nsp12, 10 µM Nsp8, 5 µM Nsp7 in 20 mM HEPES/KOH (pH 7.5), 75 mM NaCl, 2 mM $MgCl_2$ and 0.5 mM TCEP. Reactions were incubated for 30 min at 30 ^oC and the RNA extension was initiated by addition of 1 mM each ATP, CTP, GTP and UTP. Reactions were stopped by the addition of 2.5 mM EDTA (pH 8.0) followed by phenol-chloroform extraction. RNA products of extension reactions were recovered from the aqueous phase by ethanol precipitation, analyzed by electrophoresis on a 15% denaturing polyacrylamide gel and visualized by autoradiography.

RNA and DNA constucts:

long ssRNA: 36 bases (5'-UUU UCA UGC UAC GCG UAG CAU GCU ACG CGU AGC AUG-3')

short ssRNA: 28 bases (5'-CAU GCU ACG CGU AGC AUG CUA CGC GUA G-3')

The RNA and DNA constructs were highly purified synthetic polynucleotides ordered from IDT. The dsRNA consisted of duplexed long ssRNA (36 bases) paired to short ssRNA (28 bases). RNA oligos were dissolved and annealed in water. Both RNAs were validated by the SEC-MALS-SAXS experiment on a Shodex KW403 (Shodex, Tokyo Japan) column using 50 mM HEPES (pH 7.5), 150 mM NaCl, 1 mM TCEP running buffer. Both MALS and SAXS measurements agree with models of monodispersed dsRNA and ssRNA (Fig. S3). The atomistic models of both RNA's give an excellent match to the SAXS data (Fig. S3C).

Crystallization, data collection, and structural analysis of Nsp7/8 complex

For crystallizations trials we used 400 nl protein solution mixed with 400 nl of buffer reservoir solution. The sitting drops were equilibrated against 130 µl of reservoir solution. Screening of crystallization condition of Nsp7/Nsp8 complex were performed at 289K with MCSG1, MCSG2, MCSG3, MCSG4, INDEX, Natrix HT crystallizations screens (Anatrace, Hampton Research, Maumee, OH; Hampton Research, Aliso Viejo, CA). NSP7/8A (PDB: 6WIQ) crystals were obtain from 20 mg/ml protein concentration in crystallization condition 0.1 M Tris pH 8.5, 1.5 M ammonium phosphate dibasic. Crystals suitable for high-resolution diffraction data collection appeared after three weeks. For Nsp7/8B (PDB entry 6WQD) the crystal of the Nsp7/8 was obtained using 40 mg/ml of the complex in the buffer: 0.2 M magnesium chloride, 0.1 M Tris, pH 8.5, 20% w/v PEG8000. The Nsp7/8C (PDB entry 6XIP) crystal grow from 40 mg/ml in the buffer: 0.2 M magnesium chloride hexahydrate, 0.1 M BIS-TRIS pH 5.5, 25% w/v polyethylene glycol 3350. The Nsp7/8B and C (PDB entries 6WQD and 6XIP) crystals were both obtained in a presence of V8 protease. Before setting the crystallization plates to purified Nsp7/8 we added endo-proteinase Glu-C (V8 protease) from Staphylococcus aureus (Sigma Aldrich, St. Louis MO). We used 300 times excess of Nsp7/8

over V8 protease. After 16 hours of incubation of the Nsp7/8 complex with V8 protease we set up the crystallization plates.

Prior to data collection at 100 K, all cryoprotected crystals of Nsp7-Nsp8 complex were flash-cooled in liquid nitrogen. The x-ray diffraction experiments were carried out at the Structural Biology Center 19-ID beamline at the Advanced Photon Source, Argonne National Laboratory. The diffraction images were recorded from the crystals of three different forms Nsp7/8A, Nsp7/8B, and Nsp7/8C on the PILATUS3X 6M detector using 0.3-0.5° rotation on ω and 0.5 sec exposure for 210°, 375°, and 260°, and to resolution of 2.9 Å, 1.95 Å, and 1.50 Å, respectively. For Nsp7/8B crystals, the diffractions were collected from three different spots from a crystal. The data sets were processed and scaled with the HKL3000 suite (29). Intensities were converted to structure factor amplitudes in the Ctruncate program (30, 31) from the CCP4 package (32). The structures were determined using molrep (33) implemented in the HKL3000 software package using the SARS-CoV Nsp7/8 complex structure (PDB id 5F22) as a search model. Initial models were refined as rigid bodies and then refined all atoms by 12 cycles of REFMAC (32, 34) before they were iteratively refined using COOT and PHENIX (35). Throughout the refinement, the same 5% of reflections were kept out from the refinement in both REFMAC and PHENIX refinement. The final structures converged to R_{work} = 0.218 and R_{free} = 0.252 for Nsp7/8A, R_{work} = 0.187 and R_{free} = 0.229 for Nsp7/8B, R_{work} = 0.161 and R_{free} = 0.199 for Nsp7/8C with regards to each data quality. The stereochemistry of the structures were checked with PROCHECK (36) and the Ramachandran plot and validated with the PDB validation server. The data collection and processing statistics are given in Table S1. The atomic coordinates and structure factors have been deposited in the Protein Data Bank under accession code 6WIQ, 6WQD and 6XIP for Nsp7/8A, Nsp7/8B, and Nsp7/8C, respectively.

Size-exclusion chromatography coupled with multi-angle light scattering and small-angle X-ray scattering (SEC-MALS-SAXS)

For SEC-MALS-SAXS experiments, 60 μL containing either 10 mg/ml Nsp7; 8 mg/ml Nsp8; 8 mg/ml Nsp7/8; 6 mg/ml Nsp8/12; 6 mg/ml and Nsp7/8/12; in 50 mM HEPES pH 7.5, 150 mM NaCl, 1mM TCEP were utilized. Additionally, we also performed SEC-MALS-SAXS for the mixture of dsRNA or ssRNA with the Nsp7, Nsp8, Nsp7/8, Nsp12/8, and Nsp7/8/12 in 1:1 molar ratio.

SEC-MALS-SAXS data were collected at the Advanced Light Source (ALS) beamline SIBYLS (beamline 12.3.1) in Berkeley, California (37, 38). The X-ray wavelength was set at λ=1.127 Å, and the sample-to-detector distance was 2070 mm, resulting in scattering vectors, q, ranging from 0.01 $Å^{-1}$ to 0.35 $Å^{-1}$. The scattering vector is defined as $q = 4\pi \sin\theta/\lambda$, where 2θ is the scattering angle. All experiments were performed at 20°C and data was processed as described (39). Briefly, a SAXS flow cell was coupled in-line with an Agilent 1290 Infinity HPLC system using a Shodex KW803 column equilibrated at a 0.5 mL/min flow rate with the running buffer as indicated above. 55 µL of each sample was run through the SEC column and 2s X-ray exposures were collected continuously during a 20-minute elution. The SAXS frames recorded prior to the protein elution peak were used to subtract all other frames. The subtracted frames were investigated by the radius of gyration (Rg) derived by the Guinier approximation $I(q) = I(0) \exp(-q^2R_g^2/3)$ with the limits q R_g<1.5 (40). The elution peak was mapped by comparing the integral ratios to background and R_g relative to the recorded frame using the program SCÅTTER. Non-uniform Rg values across an elution peak represent a heterogeneous assembly. Final merged SAXS profiles, derived by

integrating multiple frames at the peak of the elution peak or further indicated, were used for analysis, including the Guinier plot, which determined aggregation-free state. The program SCÅTTER was used to compute the pair distribution function (P(r)). The distance r where P(r) approaches zero intensity identifies the maximal dimension of the macromolecule (Dmax) (Table S2). P(r) functions were normalized based on the molecular weight of the assemblies as determined by SCÅTTER using the volume of correlation Vc (41) (Table S2). The SAXS flow-cell was additionally connected inline to a 1290 series UV-vis diode array detector (DAD) measuring at 280 and 260 nm(Aglient), 18-angle DAWN HELEOS II multi-angle light scattering (MALS) and quasi-elastic light scattering (QELS), and Optilab rEX refractometer (Wyatt Technology, Santa Barbra CA). System normalization and calibration were performed with a BSA monomer using a 45 µL sample at 10 mg/mL in the same running buffer and a dn/dc value of 0.175. The light scattering experiments were used to perform analytical scale chromatographic separations for mass and hydrodynamic radius (Rh) determination. UV, MALS, and differential refractive index data were analyzed using Wyatt Astra 7 software to monitor sample homogeneity across the elution peak complementary to the above-mentioned SEC-SAXS signal validation. The SEC-MALS signal was used as an indicator of homogeneous vs. heterogeneous samples.

High-throughput SAXS and SANS

SAXS data were collected in "batch" high throughput mode (HT-SAXS) at the ALS beamline 12.3.1 (SIBYLS) at LBNL Berkeley (42) on Nsp8 at 10, 5 and 2.5 mg/mL because of the concentration dependence we observed in SEC-SAXS and differences from SANS data. Experiments were performed 20^0C as described elsewhere (37). Briefly, the sample was exposed for 10 s with the detector framing at 0.3 s to maximize signal while ensuring only non-radiation damaged signal is included in the data for analysis. Once radiation damage data was removed and a SAXS profile was integrated, processing occurred using the SCÅTTER package as described for the SEC-SAXS profiles.

The protein concentrations for SANS measurements were 1.5 mg/mL Nsp7/8; 4 mg/mL Nsp8; 4 mg/mL deuterated Nsp7/Nsp8 complex, and mixture of Nsp7/8/dsRNA in 1:1 molar ratio, at 3.5 mg/mL Nsp7/8 complex in the same buffer as was performed SAXS experiments. For the contrast matching SANS experiments of deuterated dNsp7 with protiated Nsp8 (dNsp7/Nsp8 complex), 90% D_2O buffer was used to selectively highlight the scattering from Nsp8. Studies of the Nsp7/8/dsRNA mixture were performed in 65% D_2O buffer to selectively highlight the scattering from Nsp7/8 complex.

SANS measurements were collected at the Bio-SANS and EQ-SANS instruments located at the High Flux Isotope Reactor and Spallation Neutron Source (SNS), respectively, at Oak Ridge National Laboratory (43, 44). At the Bio-SANS, a single configuration of the dual detector system was used with the main detector at 7m (SSD) and the wing detector at 3.2°. Using this configuration, the *q* range spanning $0.007 < q$ ($Å^{-1}$) < 1 was obtained using 6 Å wavelength neutrons with an ($\Delta\lambda/\lambda$) of 15%. At EQ-SANS, two instrument configurations were used - 2.5Å and 2.5m (SSD) for high-*q* and 10Å and 4m (SSD) for low-*q* to provide an *q*-range of 0.006 to 1.2 $Å^{-1}$. The data were corrected for instrument background, detector sensitivity, and instrument geometry using facility data reduction software, drt-SANS. All SANS measurements were performed in 1 mm path length cylindrical quartz cuvettes (Hellma, Müllheim, Germany) at 10°C. Initial SANS data analysis, including Guinier fits and pair-distribution calculations, were performed using the BioXTAS RAW program and ATSAS suite(45),(46). The pair distance distribution function (P(r)) was

calculated using the indirect Fourier transform method implemented in the program GNOM (47). Scattering data over the range 0.007 < q (Å^{-1}) < 8/R_g were used for P(r) analysis and subsequent modeling. The SAXS-derived molecular weight was determined using the volume of Porod method as implemented in RAW (48).

Solution Structure Modeling.

Nsp7: To fit experimental SEC-SAXS curves of Nsp7 dimer, we initially modeled an Nsp7 dimer by adding missing C and N-terminal regions into our Nsp7 structure taken from the Nsp7/8 crystal structure (PDBID: 6WIQ) by MODELLER (49). To test whether the discrepancy between SAXS and Nsp7 dimer structure was due to the flexibility of exposed C-terminal helix (68-86), we perform rigid body modeling using BILBOMD (19). In this step, the disulfide bond (Cys8-Cys8) was preserved. The experimental SAXS was then compared to theoretical scattering curves generated from atomistic models using the FOXS (17, 18) and followed by multistate model selection by MultiFOXS (21). The bad SAXS fit leads us to search for the alternative Nsp7 dimer by bypassing disulfide bond restraints. We performed SAXS based docking of two Nsp7 monomers using FOXSDock approach(50). The best score model was superimposed on the average SAXS envelope calculated by GASBOR with a P2 symmetry operator (51) (Fig. 3C).

Nsp8 monomer: To find an Nsp8 conformation that fits the SEC-SAXS Data, we built an Nsp8 monomer by adding missing C and N-terminal regions into the Nsp8 monomer, taken from Nsp7/8/12/RNA structure (PDBID: 6YYT)(16). We employed a conformational sampling of the N-terminal helix-bundle region using BILBOMD (19). The flexible tethers in between the head and two distinct helix regions (1-82 and 86-100) were identified by structural comparison of the two Nsp8 conformers from the Nsp7/8/12/RNA complex (16). The experimental SAXS profiles were then compared to theoretical scattering curves generated from atomistic models using the FOXS (17, 18) followed by multistate model selection by MultiFoXS (21). Despite providing a nearly exhaustive search of extended and compact conformations, an excellent fit to the SAXS data was obtained for a single closed state (Fig. 3D).

Nsp8 dimer and tetramer: To find a multistate model that fits a higher oligomerization state collected in HT-SAXS mode or at the peak of SEC elution, we built the Nsp8 dimer and tetramer. Missing regions were added to the Nsp8 dimer taken from the crystal structure of the Nsp7/8 complex (PDBID: 3UB0) (22). By keeping the dimerization interface, we used BILBOMD (19) to build an Nsp8 dimer with open and close C-terminal head regions. Additionally, we built the Nsp8 tetramer by adding missing regions into the Nsp8 tetramer taken from the crystal structure of Nsp7/8 (PDBID: 2AHM). The experimental SAXS profiles were then compared to theoretical SAXS curves generated from the pool of the monomers, dimers, and tetramer using the FOXS (17, 18) followed by multistate model selection by MultiFOXS (21) (Fig. S4).

Nsp12: To fit experimental SEC-SAXS curves of Nsp12 monomer, we add missing regions into Nsp12 structure, taken from Nsp7/8/12/RNA cryo-EM structure (PDBID: 6YYT), by using MODELLER (49). The experimental SAXS profiles were then compared to theoretical scattering curves generated from atomistic models using the FOXS (17, 18) (Fig. 3E). The experimental SANS profile of Nsp8 was compared to theoretical SANS of an Nsp8 tetramer built using the SAXS model of Nsp8 dimer (PDBID: G1TQHV) using SAXS/REFMX to perform the quaternary structure modeling (52, 53).

Nsp7/8: To find a multistate model that fit SEC-SAXS data for Nsp7/8, we initially build a complete model of Nsp7/8 heterotetramer by combining our crystal structure (PDBID: 6WIQ) and Nsp8 structure taken from Nsp7/8/12/RNA structure (PDBID: 6YYT)(16). The experimental SAXS profile was then compared to theoretical SAXS curves generated from the pool of the Nsp8 monomers, Nsp8 dimers, Nsp7 dimers, Nsp7/8 heterotetramer, and Nsp7/8 heterodimer using the FOXS (17, 18) followed by multistate model selection by MultiFOXS (21) (Fig. 4E). The experimental SANS profile of Nsp7/8 complex was compared to theoretical SANS profile using a mixture of SAXS models of Nsp7/8 heterotetramer (PDBID: DJTRUW) and Nsp8 monomer (PDBID: IDDWOG). OLIGOMER was used to determine the volume fraction of component (54). The experimental SANS profile of dNsp7/Nsp8 complex was compared to theoretical SANS profile using a mixture of SAXS models of Nsp8 dimers (PDBID: DJTRUW) and Nsp8 monomers (PDBID: IDDWOG). SAXS/REFMX was used to perform the quaternary structure modeling to account for polydispersity of the system.

Nsp8/12 and Nsp7/8/12: To find a model that fits SEC-SAXS data of Nsp8/12 and Nsp7/8/12, we initially built a complete model of Nsp7/8/12 using the cryo-EM Nsp7/8/12/RNA structure (PDBID: 6YYT) (16). The experimental SAXS curves were then compared to theoretical SAXS curves generated from the pool of the models: (Nsp7 dimer, Nsp8 monomers, Nsp8 dimers, Nsp12 monomer, Nsp8/12 with 1:1 and 2:1 ratios; and Nsp7/8/12 with 1:1:1, 1:2:1, 0:1:1, 0:2:1 ratio) using the FOXS (17, 18) followed by multistate model selection by MultiFOXS (21). Despite providing a large pool of the models to select a multistate model (21) an excellent fit for the SAXS data required only Nsp8/12 1:1 conformer to fit both data sets (Fig. 5D).

Nsp8/dsRNA: To find a model that fits SEC-SAXS data of Nsp8/dsRNA complex, we built a complete model of Nsp8dsRNA using the cryo-EM Nsp7/8/12/RNA structure (PDBID: 6YYT) (16). The flexible tethers in between the head (101-199) and helix bundle regions (1-82 and 86-100) were used to perform conformational sampling of the head region using BILBOMD (19). The experimental SAXS profiles were then compared to theoretical scattering curves generated from atomistic models using the FOXS (17, 18) followed by multistate model selection by MultiFoXS (21). Despite providing a nearly exhaustive search of extended and compact conformations, an excellent fit to the SAXS data was obtained for a single closed state (Fig. 4C). The Nsp8/dsRNA conformers pool, together with the above characterized Nsp7 dimer, were used to select a multistate model by MultiFoXS (21) for Nsp7/8+ dsRNA SEC-SAXS data (Fig. 4F).

Nsp7/8/12/dsRNA and Nsp7/8/12/ssRNA: To fit SEC-SAXS data of Nsp7/8/12/dsRNA complex, we built a complete model of Nsp7/8/12/dsRNA using cryo-EM structure (PDBID: 6YYT) (16). Despite providing a large pool of the models to select a multistate model (21) an excellent fit for the SAXS data required only a complete complex of Nsp7/8/12/dsRNA with 1:1:2:1:1 ratio (Fig. 5D). Nsp7/8/12/ssRNA fit SEC-SAXS data of Nsp7/8/12/ssRNA complex with 1:1:2:1:1 ratio model using longer ssRNA (Fig. 5D). The experimental SANS profile of Nsp7/8/dsRNA mixture was compared to theoretical SANS profile using the SAXS model of Nsp8 monomer (PDBID: 7MRNJA) CRYSON (55).

Table S1. Data Collection and Refinement Statistics

	Nsp7-8A	Nsp7-8B	Nsp7-8C
**Crystallization Conditions**			
**Data Collection**			
Space group	*C222₁*	*P2₁*	*P2₁*
Unit cell parameters (Å;°)	*a* = 52.15, *b* = 70.78, *c* = 115.46; α = β = γ = 90.0	*a* = 47.26, *b* = 55.10, *c* = 84.40; α = γ = 90.0, β = 90.4	*a=35.62, b=119.90, c=43.38;* α = γ = 90.0, β= 92.2
Resolution range (Å) [a]	50.00 – 2.85 (2.90 - 2.85)	50.00 - 1.95 (1.98 - 1.95)	50.00 – 1.50 (1.53-1.50)
No. of reflections	5,144 (235) [a]	30,923 (1,220)	56,701 (2,392)
R_{merge} [b]	0.13 (1.11)	0.18 (1.15)	0.083 (0.84)
Completeness (%)	97.9 (88.7)	97.3 (77.9)	97.7 (83.9)
$CC_{1/2}$ [c]	0.853 (0.468)	0.962 (0.456)	0.999 (0.826)
$I/\sigma(I)$	18.2 (1.01)	22.5 (1.1)	30.2 (1.85)
Multiplicity	6.7 (4.4)	8.7 (2.2)	4.7 (3.0)
Wilson *B* factor	105.9	38.6	22.8
**Structure Determination**			
MR initial model (PDB ID)	5F22	6WIQ	6WIQ
**Refinement**			
Resolution range (Å)	35.39 - 2.85 (3.59 - 2.85)	41.12 - 2.00 (2.01 - 1.95)	40.76 - 1.50 (1.53 -1.50)
Completeness (%)	97.2 (96.0)	96.2 (73.0)	97.6 (82.0)
No. of reflections	5,108 (2,462)	30,700 (2,095)	56,635 (2,468)
R_{work}/R_{free} [d] (%)	20.8/25.2 (31.2/32.3)	18.7/22.9 (32.6/41.4)	16.1/19.9 (21.6/25.5)
Protein chains/atoms	2/1,507	4/3,077	4/2,988
Ligand/Solvent atoms	-	12/84	20/235
Mean temperature factor (Å^2)	125.6	53.1	35.5
**Coordinate Deviations**			
R.m.s.d. bonds (Å)	0.001	0.008	0.011
R.m.s.d. angles (º)	0.357	0.829	1.158
**Ramachandran plot** [e]			
Favored (%)	96.86	98.04	99.19
Allowed (%)	2.62	1.68	0.81
Outside allowed (%)	0.52	0.28	0.0
**PDB Accession Code**	6WIQ	6WQD	6XIP

[a] Values in parentheses correspond to the highest resolution shell.
[b] Rmerge = ΣhΣj|Ihj–<Ih>|/ΣhΣjIhj, where Ihj is the intensity of observation j of reflection h.
[c] As defined by Karplus and Diederichs (56). [d]R = Σh|Fo|–|Fc|/Σh|Fo| for all reflections, where Fo and Fc are observed and calculated structure factors, respectively. R_{free} is calculated analogously for the test reflections, randomly selected and excluded from the refinement. [e]As defined by Molprobity (57)

Table S2. Structural parameters from SAXS, SANS and MALS data

SAXS sample SimpleScattering ID#	D_{max}[a] (Å)	R_g (Å) from Guinier plot	Rg (Å) from P(r)	MW Seq. Monomer (kDA)[b]	MW SAXS[b] or SANS (kDa)	MW MALS (kDa)	Model fit[d] χ^2	data source
Nsp7 XSJUFZRI	~ 65	18.6± 0.6	18.8	9	19	17	1.5	SEC-SAXS peak
Nsp8 XSIDDWOG	~ 90	24.6± 0.7	25.8	22	29	28	1.4	SEC-SAXS tail
Nsp8 XSG1TQHV	~ 110	26.8± 0.1	27.8	22	33	35	1.9	SEC-SAXS peak
Nsp8	~ 110	29.9± 0.9	30.6	22	38	ND	ND	SANS
Nsp12 XSQIWPPC	~ 110	31.1± 0.5	41.9	107	88	93	1.3	SEC-SAXS
Nsp7/8 XSDJTRUW	~ 100	28.8± 0.1	30.3	31	42	45	2.2	SEC-SAXS peak
Nsp7/8	~ 110	30.0± 0.1	ND	31	56	ND	ND	SANS
Nsp8+dsRNA XS7MRNJA	~ 120	32.2± 0.6	29.8	45	46	45	1.7	SEC-SAXS peak
Nsp8+ssRNA XSXMU3AK	~105	30.3± 0.2	31.4	33	35	35	ND	SEC-SAXS peak
Nsp7/8 +dsRNA XSTKZWCR	~ 105	28.9± 0.2	31	54	32	30	2.1	SEC-SAXS peak
Nsp8/12 XSPPENAB	~ 115	34.8± 0.5	35.2	129	118	131	1.4	SEC-SAXS peak
Nsp7/8/12 XSX1TKUI	~ 115	34.7± 0.7	35.2	138	114	132	2.2	SEC-SAXS peak
Nsp7/8/12 +dsRNA XSZWMCYC	~ 140	41.8± 1.7	43.0	159	170	195	1.8	SEC-SAXS peak
Nsp7/8/12 +ssRNA XSE38FZM	~ 145	41.1± 2.2	43.0	126	170	190	1.7	SEC-SAXS peak

a) Maximal dimension (Dmax) defined from pair distribution function (P(r)) calculated by SCATTER
b) Theoretical MW calculated for monomer or assembly within the equal molar ratio
c) MW determined by volume of correlation Vc (41)
d) Goodness of fit (χ^2) for the atomistic models is determined FOXS(17, 18) for single model or MultiFOXS(50) for multistate model.

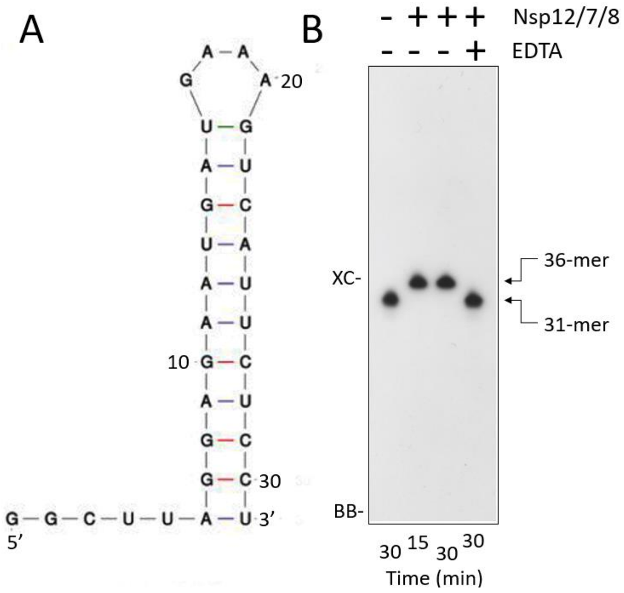

FIGURE S1. SARS-CoV-19 RTC activity**.** (A) Minimal substrate for RTC: a hairpin composed of 31 nucleotides. (B) Analysis of the minimal substrate extension by the Nsp12/7/8 complex on a 15% denaturing polyacrylamide gel. The hairpin was [^{32}P]-labeled and its extension was monitored for 30 min. 2.5 mM EDTA inhibits the extension reaction. BB and XC denote positions bromophenol blue

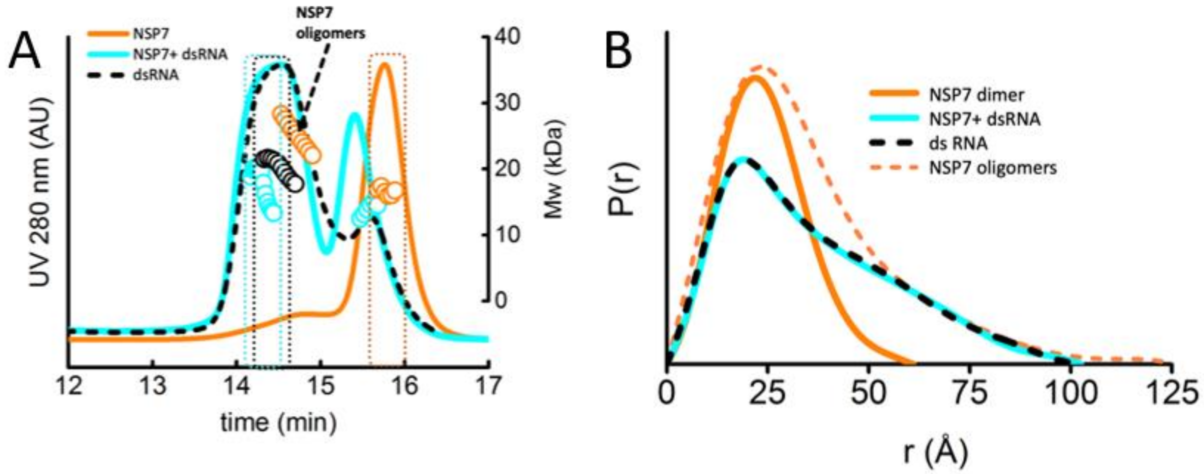

FIGURE S2 Nsp7 does not interact with dsRNA. (*A*) SEC-MALS chromatograms for Nsp7, Nsp7+dsRNA, and dsRNA are colored as indicated. Solid lines represent the UV at 280nm in arbitrary units, while symbols represent molecular mass versus elution time. (*B*) Normalized P(r) function calculated for SAXS profiles of Nsp7 dimer, Nsp7+dsRNA, and dsRNA collected at the SEC peak and Nsp7 oligomers collected at the SEC-leading peak. The similarity of P(r) functions between Nsp7+dsRNA and dsRNA further confirms the absence of Nsp7+dsRNA complexation.

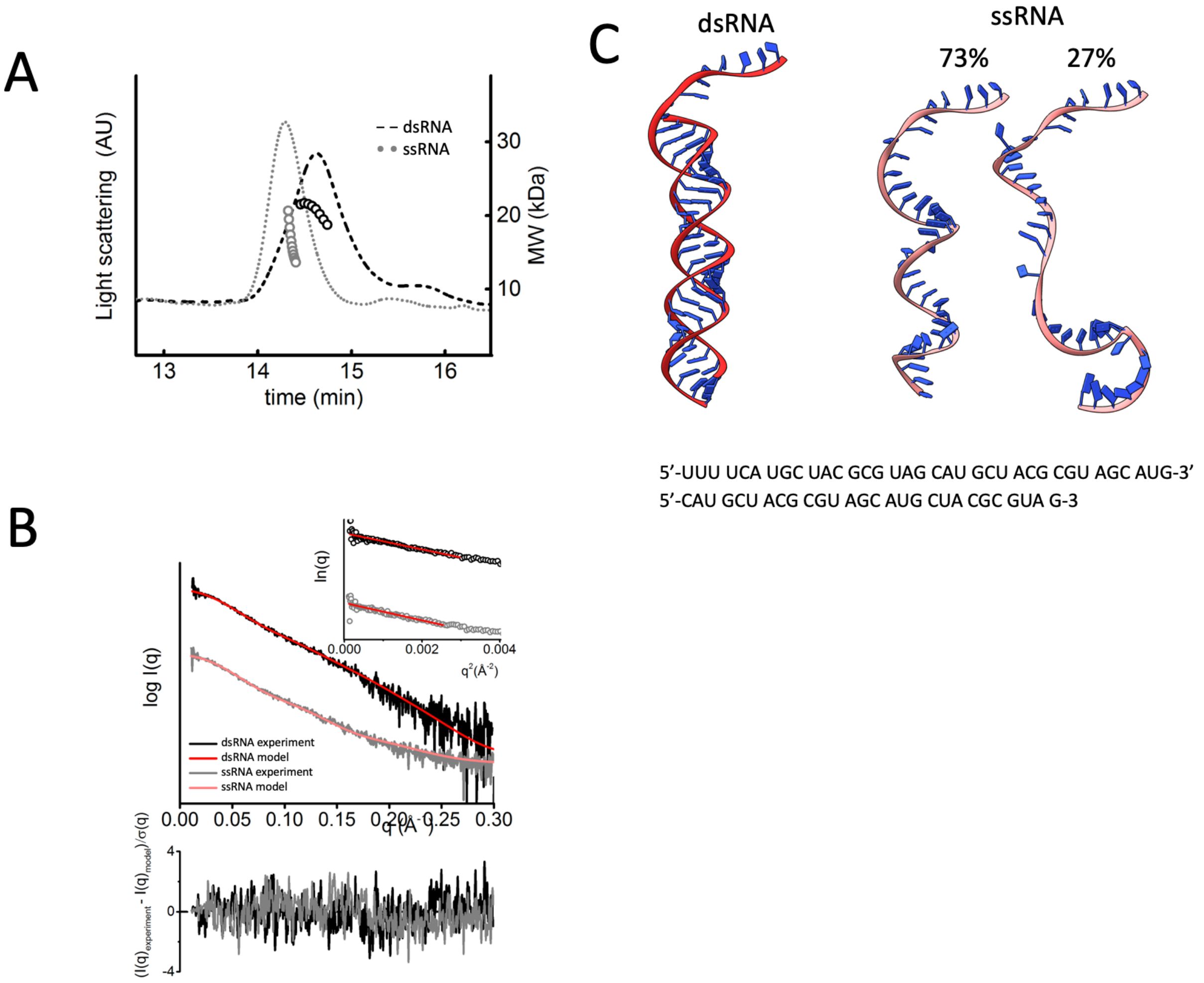

FIGURE S3. Monodisperse dsRNA and ssRNA. (*A*) SEC-MALS chromatograms for dsRNA and dsRNA are colored as indicated. Solid lines represent the UV at 280nm in arbitrary units, while symbols represent molecular mass versus elution time. (*B*) Experimental SAXS profiles for dsRNA (black) and ssRNA (gray) collected at the SEC peak are shown together with the theoretical SAXS profiles for solution-state models of ds RNA (red) and ssRNA (light red) shown in panel C. SAXS fits are shown together with the fit residuals and goodness of fit values (χ^2). Guinier plots for experimental SAXS curves are shown in the inset. (*C*) Solution state models for dsRNA and ssRNA were used to fit experimental data shown in panel B.

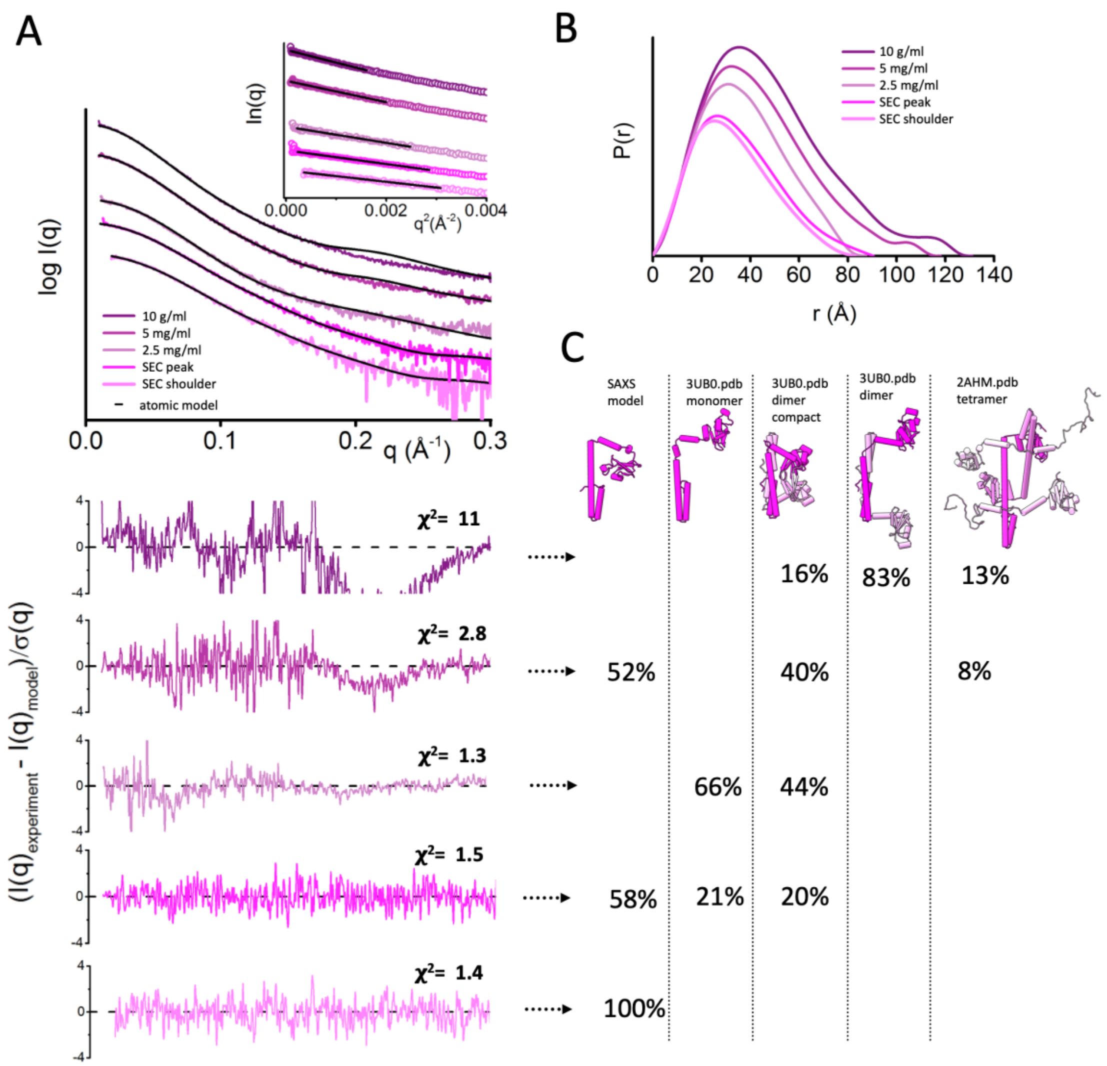

Figure S4 Nsp8 forms a dimer and larger oligomer at high concentration. (*A*) Experimental SAXS profiles for Nsp8 monomer, dimer, and the sizeable oligomeric state collected at SEC-SAXS elution shoulder and peak compared to the SAXS data collected in HT-SAXS mode at 2.5, 5, and 10 mg/ml (colored as indicated). SAXS data are shown together with a theoretical SAXS profile for multistate models shown in panel C and weights as indicated. SAXS fits are shown together with the fit residuals and goodness of fit values (χ^2). Guinier plots for experimental SAXS curves are shown in the inset. (*B*) P(r) functions calculated for the experimental SAXS profiles from panel A are normalized on the molecular mass determined by the volume of correlation Vc (41) (*C*) Solution state models for Nsp8 at various concentrations that fit experimental data shown in panel A.

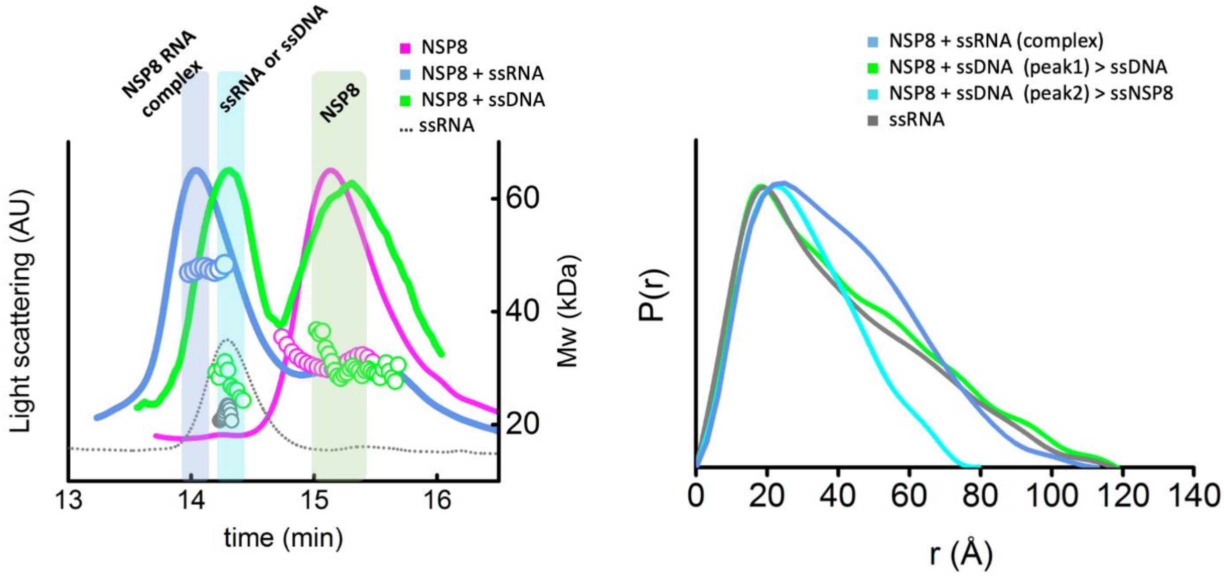

FIGURE S5 Nsp8 binds ssRNA but not ssDNA. (left) SEC-MALS-SAXS elution profiles of Nsp8, ssRNA, Nsp8 with ssRNA, and Nsp8 with ssDNA. The ssRNA is an analogous sequence and has the same length as the ssDNA. An analysis of the MALS data in each peak provides a mass as indicated by the circular points with values indicated on the right axis. The SAXS curves that were extracted from each peak containing either ssDNA or ssRNA are shown (left). The Nsp8 + ssRNA is distinct from all other curves.

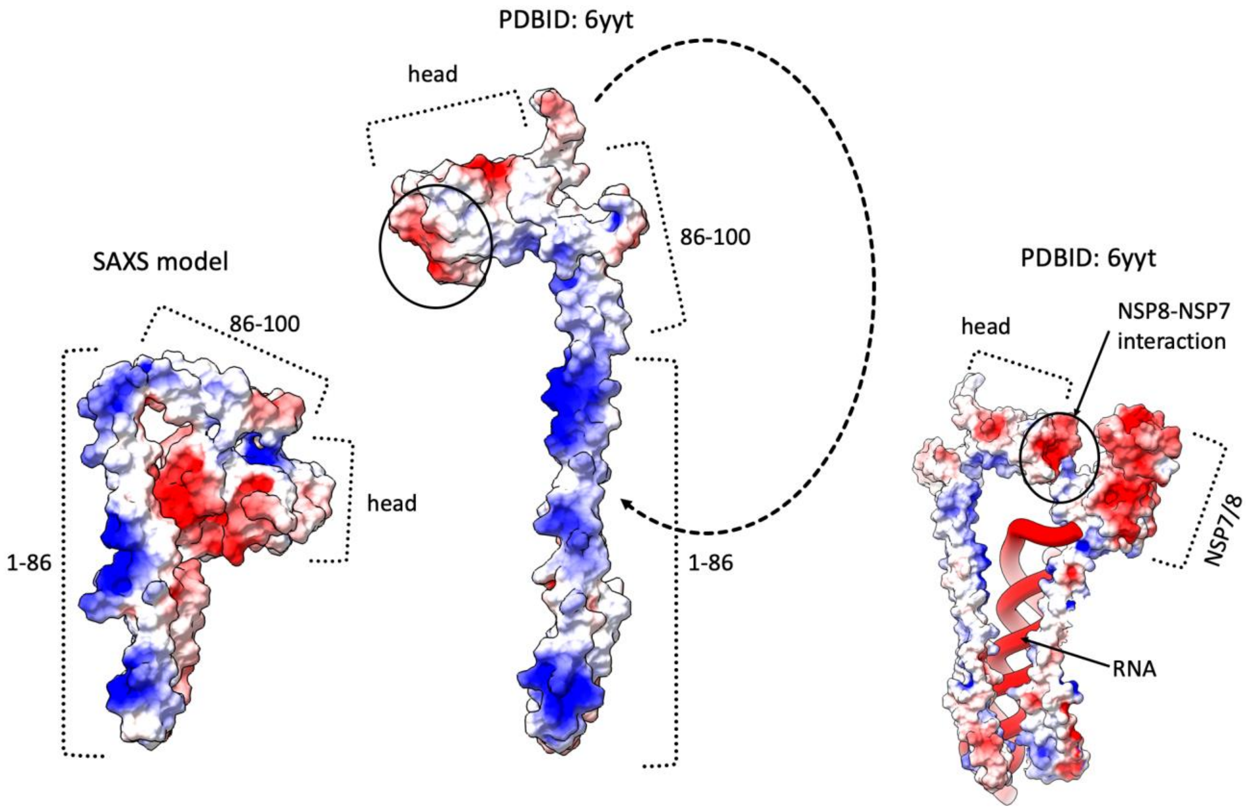

FIGURE S6 Distribution of electrostatic surface potential and flexibility of the Nsp8 helix bundle drive the compaction of Nsp8. Electrostatic surface potential for Nsp8 for SAXS model and Nsp8 conformer taken from the cryo-EM structure of Nsp7/8/12/dsRNA (PDBID: 6yyt) calculated at the pH= 7.0. The surface potential indicates that the flexible N-terminal helix bundle region (1-100) is mostly positively charged and is suited for nucleic acid-binding. In the absence of Nsp7 or Nsp12 (right panel), the negatively charged head region folds back (SAXS model) and interacts with the positively charged helix-bundle region.

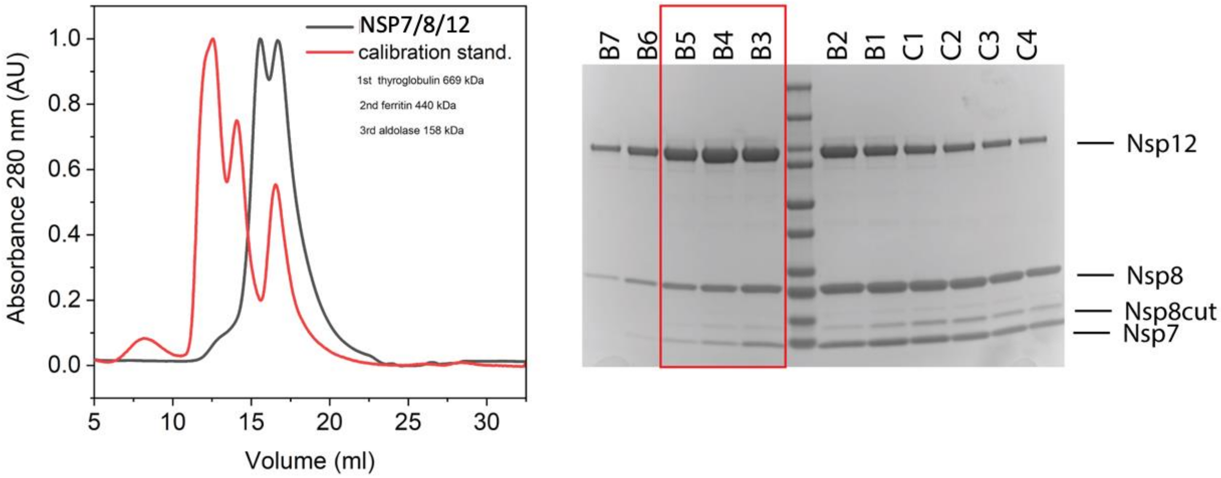

FIGURE S7 Initial SEC purification of Nsp7/8/12 sample. Splitting of SEC peak (left panel) shows heterogeneity of Nsp7/8/12 complex. The shift of Nsp8, Nsp7 bands relative to the Nsp12 on the SDS-PAGE gel (right panel) further indicates weak Nsp7/8/12 complex.
