# Supplementary material for: Transient and stabilized complexes of Nsp7, Nsp8, and Nsp12 in SARS-CoV-2 replication
Source: Biophys J. 2021 Jun 29;120(15):3152–65. doi: 10.1016/j.bpj.2021.06.006 (PMC8238635; doi:10.1016/j.bpj.2021.06.006)
Supplement: Document S2. Article plus supporting material [file mmc2.pdf]

Biophysical Journal
Article

Transient and stabilized complexes of Nsp7, Nsp8, and Nsp12 in SARS-CoV-2 replication

Mateusz Wilamowski,[1,2] Michal Hammel,[3] Wellington Leite,[4] Qiu Zhang,[4] Youngchang Kim,[1,5] Kevin L. Weiss,[4] Robert Jedrzejczak,[1,5] Daniel J. Rosenberg,[3,6] Yichong Fan,[4] Jacek Wower,[7] Jan C. Bierma,[3] Altaf H. Sarker,[3] Susan E. Tsutakawa,[3] Sai Venkatesh Pingali,[4] Hugh M. O'Neill,[4,*] Andrzej Joachimiak,[1,2,5,*] and Greg L. Hura[3,8,*]

[1]Center for Structural Genomics of Infectious Diseases, Consortium for Advanced Science and Engineering and [2]Department of Biochemistry and Molecular Biology, University of Chicago, Chicago, Illinois; [3]Molecular Biophysics and Integrated Bioimaging Division, Lawrence Berkeley National Lab, Berkeley, California; [4]Center for Structural Molecular Biology, Neutron Scattering Division, Oak Ridge National Laboratory, Oak Ridge, Tennessee; [5]Structural Biology Center, X-ray Science Division, Argonne National Laboratory, Argonne, Illinois; [6]Graduate Group in Biophysics, University of California, Berkeley, Berkeley, California; [7]Department of Animal Sciences, Auburn University, Auburn, Alabama; and [8]Chemistry and Biochemistry Department, University of California Santa Cruz, Santa Cruz, California

ABSTRACT The replication transcription complex (RTC) from the virus SARS-CoV-2 is responsible for recognizing and processing RNA for two principal purposes. The RTC copies viral RNA for propagation into new virus and for ribosomal transcription of viral proteins. To accomplish these activities, the RTC mechanism must also conform to a large number of imperatives, including RNA over DNA base recognition, basepairing, distinguishing viral and host RNA, production of mRNA that conforms to host ribosome conventions, interfacing with error checking machinery, and evading host immune responses. In addition, the RTC will discontinuously transcribe specific sections of viral RNA to amplify certain proteins over others. Central to SARS-CoV-2 viability, the RTC is therefore dynamic and sophisticated. We have conducted a systematic structural investigation of three components that make up the RTC: Nsp7, Nsp8, and Nsp12 (also known as RNA-dependent RNA polymerase). We have solved high-resolution crystal structures of the Nsp7/8 complex, providing insight into the interaction between the proteins. We have used small-angle x-ray and neutron solution scattering (SAXS and SANS) on each component individually as pairs and higher-order complexes and with and without RNA. Using size exclusion chromatography and multiangle light scattering-coupled SAXS, we defined which combination of components forms transient or stable complexes. We used contrast-matching to mask specific complex-forming components to test whether components change conformation upon complexation. Altogether, we find that individual Nsp7, Nsp8, and Nsp12 structures vary based on whether other proteins in their complex are present. Combining our crystal structure, atomic coordinates reported elsewhere, SAXS, SANS, and other biophysical techniques, we provide greater insight into the RTC assembly, mechanism, and potential avenues for disruption of the complex and its functions.

SIGNIFICANCE The SARS-CoV-2 virus has been implicated in 3,000,000 deaths. No therapeutic has been developed with sufficient efficacy to change the course of the pandemic. The single US-approved therapeutic targets the replication transcription complex (RTC), which is responsible for copying the viral RNA genome to pass to new virus. Here, we structurally characterize three components of the RTC with and without RNA using crystallography and solution small-angle scattering. Combining the structural information on each component on its own and in combinatorial mixtures, we develop an understanding of how the RTC assembles. This understanding provides insights into the RTC functions and assembly processes that could be inhibited. This impacts not only SARS-CoV-2 but most RNA viruses.

INTRODUCTION

The severe acute respiratory syndrome coronavirus 2 (SARS-CoV-2) virus has plagued every populated continent and has been implicated in 3,000,000 deaths at the time of writing. Recently deployed vaccines have provided much hope. However, distribution, voluntary immunization, and mutations in the virus remain major concerns. The development and application of treatments that attack fundamental

Submitted March 4, 2021, and accepted for publication June 4, 2021.

*Correspondence: oneillhm@ornl.gov or andrzejj@anl.gov or glhura@lbl.gov

Mateusz Wilamowski, Michal Hammel, and Wellington Leite contributed equally to this work.

Editor: Wendy Shaw.

https://doi.org/10.1016/j.bpj.2021.06.006

This is an open access article under the CC BY license (http://creativecommons.org/licenses/by/4.0/).

viral machinery will remain critical for the foreseeable future.

In the US thus far, a single drug, remdesivir, has been conditionally approved for specifically targeting the virus. It targets the RNA-dependent RNA polymerase (RdRp) (1). SARS-CoV-2 RdRp, also referred to as nonstructural protein 12 (Nsp12), is the catalytic center of the macromolecular complex frequently referred to as the replication transcription complex (RTC). The RTC is essential for virus replication because it makes copies of genomic and subgenomic RNAs and polymerizes antisense RNA. After maturation, these RNAs serve as mRNAs to produce virus Nsps, which are structural and accessory proteins. Copies of genomic RNA are eventually packaged into mature virions that bud out of host cells. The remdesivir triphosphate is incorporated into newly synthesized RNA by RdRp and either stalls or interrupts viral RNA polymerization.

Although the coronavirus genome is larger than most viruses (~30 kbs), it remains 100,000 times smaller than that of humans. The highly evolved and efficient SARS-CoV2 RTC is a major part of how the virus overcomes the limitations of its genome size. The RTC specifically identifies and polymerizes viral over host RNA without activating host defenses. The RTC contextually balances synthesis of subgenomic RNAs for use in translation of viral proteins and polymerization of genomic RNA for new virus maturation. The subgenomic RNA produced must be 3′ polyadenylated and 5′ capped, conforming to host ribosome mRNA conventions. The RTC can discontinuously read long RNAs and produce shorter subgenomic RNAs that serve as mRNAs that code for structural and accessory proteins within the long RNA (discontinuous transcription) (2). The mechanisms must be robust to varied attacks by the host cell defenses. All this must be done while retaining primary polymerase activities of recognizing initiation and termination sequences, discriminating DNA versus RNA bases, and basepairing. Reverse engineering this complex macromolecule is likely to be a challenge. However, as a central mechanism with so many functions, several avenues for sabotage by small molecules or proteins may be possible beyond the one exploited by remdesivir.

In SARS-CoV-2, besides RdRp (Nsp12), the RTC involves nonstructural proteins Nsp7, Nsp8, and others (3–5). For an excellent review that largely summarized our understanding before the pandemic, see Snijder et al. (6). Nsp12 is the largest component of the complex, and Nsp7 and Nsp8 are considered cofactors.

Several additional functions have been reported for Nsp7 and 8. Both Nsp7 and 8 are transcribed at higher rates relative to Nsp12 as part of open reading frame 1a and earlier in the infection so they may act to prepare the cell for viral replication. In a thorough study, Nsp8 has been shown to polyadenylate RNA (7), which could play a role in 3′ mRNA preparation. Nsp8 has also been implicated in blocking ribosomal membrane protein recognition signals (8). Crystal structures and other supporting methods have visualized Nsp7/8 in a ring-like hexadecamer (9), which provocatively suggests a processive sliding complex on double-stranded RNA with a possibly independent role.

Our understanding of the RTC has been remarkably advanced through recent cryo-electron microscopy (cryo-EM) studies resolving structures of Nsp7/8/12 with and without RNA (1,4,10,11). Most recently, structures of Nsp7/8/12/13 (12,13) and Nsp7/8/9/12/13 (14) have been reported. However, many questions remain about connecting the cryo-EM structures with the assembly, the role of cofactors, and the mechanism. The integration of many structural, biochemical, and genomics studies will be required to provide a mechanistic and actionable model of each protein contributing to the RTC and to assess their assembly, varied functions, and potential vulnerabilities.

Toward the goal of understanding the macromolecular machinery of the RTC, we have undertaken a systematic study of individual components and their complexes. We have solved a 1.5 Å x-ray macromolecular crystal structure of the complex between Nsp7 and Nsp8 (Fig. 1). This structure, along with those solved by others (15), particularly the recent cryo-EM structures of the Nsp7/8/12 RNA complex (10), provided key information for the interpretation of our solution scattering measurements. We have used solution scattering along with other biochemical techniques to study each protein in isolation, pairs (Nsp7/8, Nsp8/12, Nsp7-RNA, and Nsp8-RNA), ternary complexes, and all four components together (Nsp7/8/12 RNA) (Figs. 2, 4, 5, and 6).

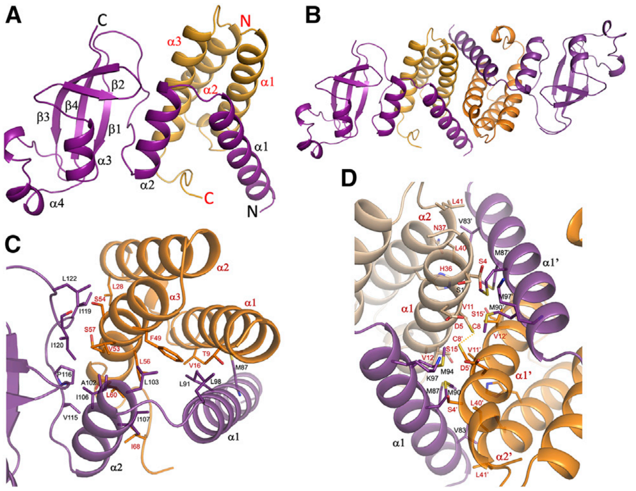

FIGURE 1 Structure of Nsp7/8 complex. The crystallized structure of the heterodimer Nsp7/8 (*A*) shows Nsp7's (*orange*) C-terminal helices intercalated between Nsp8's (*purple*) long $\alpha 1$ N-terminal helix (truncated in our structure) and $\alpha 2$. The heterotetramer structure (*B*) is also shown and present in all three of our crystal forms. Details of the heterodimer interface are shown in (*C*). Details of the heterotetramer interface are shown in (*D*), with interacting residues shown by sticks labeled red for Nsp7 and labeled black for Nsp8. An interchain, symmetrically formed disulfide bond in Nsp7 formed by C8 is shown in dashes.

Macromolecular Mix	Number of SEC-MALS-SAXS peaks	Apparent mass of peak(s), (kDa)	Model that fits SAXS in peak
Nsp7	2 (Fig S2)	>30, **17**	
Nsp8	1 (Fig 4A)	40 → 28	
Nsp12	1 (Fig 4A)	105	
ssRNA	1 (Fig 4A)	~20	
dsRNA	1 (Fig 4A)	~15	
Nsp7/8	2 (Fig 4A)	**50 →36**, 17	
Nsp8/12	2 (Fig 5A)	>500, **125**	Aggregates,
Nsp7/8/12	3 (Fig 5A)	**125**, 100, 17	
Nsp7 dsRNA	2 (Fig S2)	~15, 17	
Nsp8 ssRNA	1 (Fig 4A)	45→35	
Nsp8 dsRNA	2 (Fig 4A)	45,**45→20**	
Nsp7/8/12 ssRNA	3 (Fig 5A)	**190**, ~120, ~100	
Nsp7/8/12 dsRNA	3 (Fig 5A)	**195**, ~120, ~100	

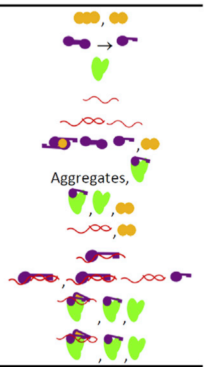

FIGURE 2 Nsp7, Nsp8, Nsp12, and RNA complexes found in elution profiles from SEC-MALS-SAXS. Each molecule was measured independently and is represented pictorially (*top left*). The number of peaks and the figure associated with the elution profile is indicated in the second column. The apparent mass through each peak is indicted in the third column. When an elution has more than one peak, the largest peak is indicated by bold mass. In some peaks, the mass changes across the peak. Mass values prefaced with ~ indicate weak signals by MALS because of low abundance. For comparison, the calculated masses for monomeric Nsp7, Nsp8, and Nsp12 are 9, 24, and 100 kDa respectively. The rightmost column depicts mixtures of models that fit the SAXS data through analysis described in the remainder of the text.

Because many proteins in the viral genome are expected to have multiple functions that require conformational or assembly changes, heterogeneities are expected in solution. All complexes were examined during elution from size exclusion chromatography (SEC) by multiangle light scattering (MALS) and small-angle x-ray scattering (SAXS): SEC-MALS-SAXS. MALS provides mass and SAXS informs on structure at every point in elution. Together, they detail the oligomeric and conformational heterogeneity characteristic of each mixture, complementing the cryo-EM and crystallographic results. To further interpret complexes, we also used small-angle neutron scattering (SANS) and its capability to contrast match out components within complexes. Using these and other biochemical techniques, we provide insights into the function, the assembly process, and the dynamics and identify stable complexes that are tractable for further mechanistic interrogation.

MATERIALS AND METHODS

Further detailed methods are provided in the Supporting materials and methods.

Gene cloning, protein expression, and purification

NCBI reference sequences for Nsp7, Nsp8, and Nsp12 are YP_009725303.1, YP_009725304.1, and YP_009725307.1, respectively, and are further available in the Supporting materials and methods. SARS-CoV-2 (taxid: 2697049) genes were expressed in *Escherichia coli* (16) and included N-terminal His_6 (histidine)-TEV (tobacco etch mosaic virus) affinity tags that were subsequently cleaved. For SANS, deuterium-labeled Nsp7 was produced using previously described methods (17). Proteins were purified first via His_6-tag using an Ni^{+2} column, with subsequent cleavage using TEV protease, and finally by SEC. Samples were concentrated and dialyzed using centrifugation and dialysis membranes.

RNA and DNA constructs

The [^{32}P]-labeled RNA used in the function testing extension assay was synthesized using previously described methods (18), folds into a hairpin, and has the sequence 5′-pppGGCUUAGGAGAUGAUGAAAGUCAUU CUCCU-OH-3′. The 36 base long single-stranded RNA (ssRNA) had the sequence (5′-UUU UCA UGC UAC GCG UAG CAU GCU ACG CGU AGC AUG-3′), and the 28 base short ssRNA had the sequence (5′-CAU GCU ACG CGU AGC AUG CUA CGC GUA G-3′). Both long and short ssRNA were ordered from IDT.

Crystallization, data collection, and structural analysis of the Nsp7/8 complex

Crystallization trials using 400 nL volumes were performed with MCSG1, MCSG2, MCSG3, MCSG4, INDEX, Natrix high-throughput crystallizations screens (Anatrace, Hampton Research, Maumee, OH). The x-ray diffraction experiments were carried out at the Structural Biology Center 19-ID beamline at the Advanced Photon Source, Argonne National Laboratory. The data sets were processed and scaled with the HKL3000 suite (19), the Ctruncate program (20,21) from the CCP4 package (22), and molrep (23) using the SARS-CoV Nsp7/8 complex structure (Protein Data Bank (PDB): 5F22) as a search model. Further refinement used REFMAC (22,24), COOT, and PHENIX (25). Throughout the refinement, the same 5% of reflections were kept out from the refinement in both REFMAC and PHENIX refinement. The final structures converged to $R_{work} = 0.218$ and $R_{free} = 0.252$ for Nsp7/8A, $R_{work} = 0.187$ and $R_{free} = 0.229$ for Nsp7/8B, and $R_{work} = 0.161$ and $R_{free} = 0.199$ for Nsp7/8C with regards to the data quality of each structure. The stereochemistry of the structures was checked with PROCHECK (26) and the Ramachandran plot and validated with the PDB validation server. The data collection and processing statistics are given in Table S1. The atomic coordinates and structure factors have been deposited in the PDB under accession codes 6WIQ, 6WQD, and 6XIP for Nsp7/8A, Nsp7/8B, and Nsp7/8C, respectively.

SEC-MALS-SAXS

SEC-MALS-SAXS data were collected at the advanced light source (ALS) beamline SIBYLS (beamline 12.3.1) in Berkeley, California (27,28). The x-ray wavelength was set at $\lambda = 1.127$ Å, and the sample-to-detector distance was 2070 mm, resulting in scattering vectors, q, ranging from 0.01 to 0.35 $Å^{-1}$. The scattering vector is defined as $q = 4\pi \sin\theta/\lambda$, where 2θ is the scattering angle. All experiments were performed at 20°C, and data were processed as described (29). The subtracted frames were investigated by the radius of gyration (R_g) derived by the Guinier approximation $I(q) = I(0)\exp(-q^2R_g^2/3)$ with the limits $q R_g < 1.5$ (30). The elution peak was mapped by comparing the integral ratios to background and R_g relative to the recorded frame using the program SCÅTTER. The program SCÅTTER was used to compute the pair-distribution function (P(r)). P(r) functions were normalized based on the molecular mass of the assemblies as determined by SCÅTTER using the volume of correlation Vc (Table S2; (31)). The SAXS flow cell was additionally connected inline to a 1290 series ultraviolet-visible light diode array detector measuring at 280 and 260 nm (Agilent, Santa Clara, CA), 18-angle DAWN HELEOS II MALS and quasielastic light scattering, and Optilab rEX refractometer (Wyatt Technology, Santa Barbra, CA).

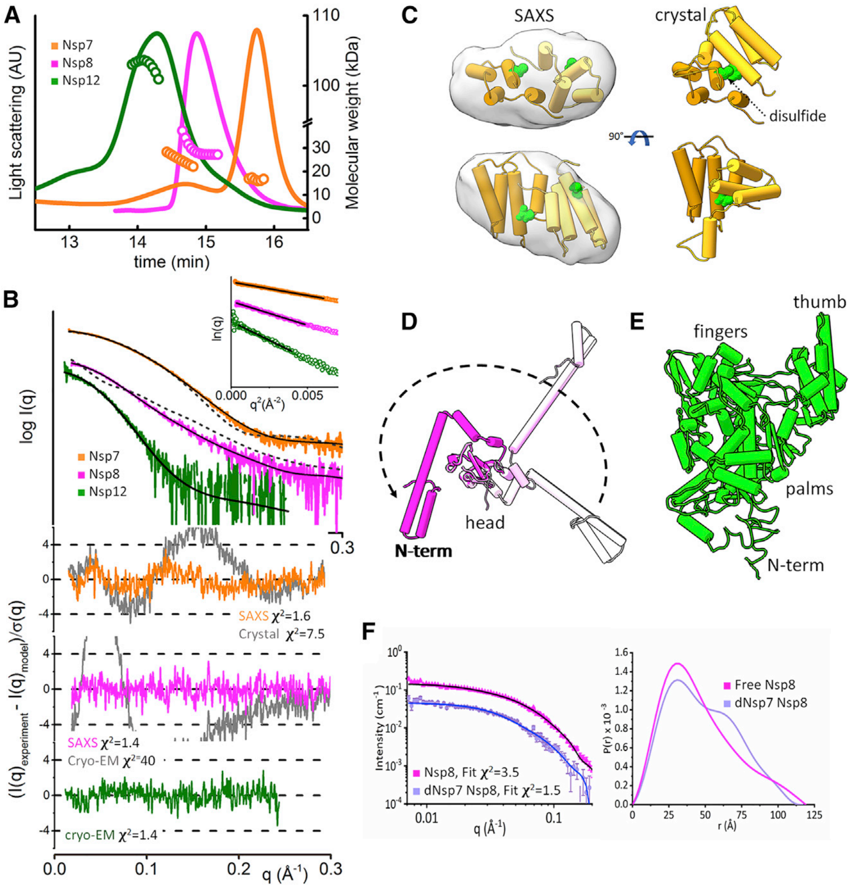

FIGURE 3 Solution states of independent Nsp7, Nsp8, and Nsp12 (*A*) SEC-MALS-SAXS elution profiles for Nsp7 (*orange*), Nsp8 (*purple*), and Nsp12 (*green*) by light scattering intensity (*solid lines*, *left axis*), with mass indicated by circles (*right axis*). (*B*) Experimental (*colored lines*) SAXS profiles for each protein. Guinier plots for experimental SAXS curves are shown in the inset. Calculated best-fit models (*solid black lines*) and alternate models from available structures (*dashed lines*) are shown along with residuals (*lower plot*, *gray* for alternate models) and goodness-of-fit parameter χ^2. (*C*) Best-fit model for Nsp7 is an alternate dimer than that found in our crystal structure, with the disulfide forming Cys8 shown in green. The average SAXS envelop is superimposed on the SAXS model. (*D*) The Nsp8 monomer is found in a thus far unobserved conformation (*dark magenta*) relative to the N-terminal (N-term) domain in the superimposed atomically resolved cryo-EM structures (*pink*) (PDB: 6YYT). (*E*) Nsp12 measurements agree with available atomic structures (PDB: 6YYT). (*F*) SANS profiles (*left*) were measured for Nsp8 (*magenta*) and dNsp7-Nsp8 complex (*light blue circles*) in 90% D_2O, masking dNsp7. Fits to models described in the text are shown in black and blue, respectively. The P(r) calculated from SANS for both (*right plot*) shows Nsp7 alters Nsp8 structure.

High-throughput SAXS and SANS

SAXS data were collected in "batch" high-throughput mode at the ALS beamline 12.3.1 (SIBYLS) at Lawrence Berkeley National Laboratory (32) on Nsp8 at 10, 5, and 2.5 mg/mL because of the concentration dependence we observed in SEC-SAXS and differences from SANS data. Experiments were performed at 20°C as described elsewhere (27).

SANS measurements were collected at the Bio-SANS and EQ-SANS instruments located at the High Flux Isotope Reactor and Spallation Neutron Source, respectively, at Oak Ridge National Laboratory (33,34). The protein concentrations for SANS measurements were 1.5 mg/mL Nsp7/8, 4 mg/mL Nsp8, 4 mg/mL deuterated Nsp7/Nsp8 complex, and mixture of Nsp7/8/double-stranded RNA (dsRNA) in 1:1 molar ratio at 3.5 mg/mL Nsp7/8 complex in the same buffer as the SAXS experiments were performed in. For the contrast-matching SANS experiments of deuterated dNsp7 with protiated Nsp8 (dNsp7/Nsp8 complex), 90% D_2O buffer was used to selectively highlight the scattering from Nsp8. Studies of the Nsp7/8/dsRNA mixture were performed in 65% D_2O buffer to selectively highlight the scattering from the Nsp7/8 complex. Initial SANS data analysis, including Guinier fits and pair-distribution calculations, was performed using the BioXTAS RAW program and ATSAS suite (35,36). The SAXS-derived molecular mass was determined using the volume of Porod method as implemented in RAW (37).

Solution structure modeling

Tools used to find conformations and assemblies of each of the structures included MODELER (38), BILBOMD (39), FOXS (40,41), MultiFOXS (42), FOXSDock (43), OLIGOMER (44), CRYSON (45), SAXS/REFMX (46,47), and GASBOR (48). To help build models that fitted the SAXS data, aside from those reported here, we relied on the following structures—PDB: 6YYT (10), PDB: 3UB0 (49), PDB: 2AHM. Full details are available in the Supporting materials and methods.

RESULTS

Atomic-resolution crystal structure of Nsp7/8

To provide insights into the structures, key residues of association, and multimeric state between the SARS-CoV-2 RTC's cofactors, we solved high-resolution crystal structures of Nsp7 bound to Nsp8. Structures were determined in three different crystal forms (Nsp7/8A, Nsp7/8B, and Nsp7/8C) and deposited in the PDB (PDB: 6WIQ, 6WQD, and 6XIP, Table S1) early in the pandemic. Our deposited structures are similar to that reported later by Konkolova et al. (15). Crystals of Nsp7/8A were shown to have a truncated N-terminus (at residue Glu78) for Nsp8. The other structures were obtained from crystallization in the presence of protease Glu-C (V8). Below, the description of the Nsp7/8 structure is based on the 1.5 Å structure Nsp7/8C, unless otherwise mentioned.

The structures of Nsp7 and Nsp8 have strong similarities to the same proteins and variants from other coronaviruses.

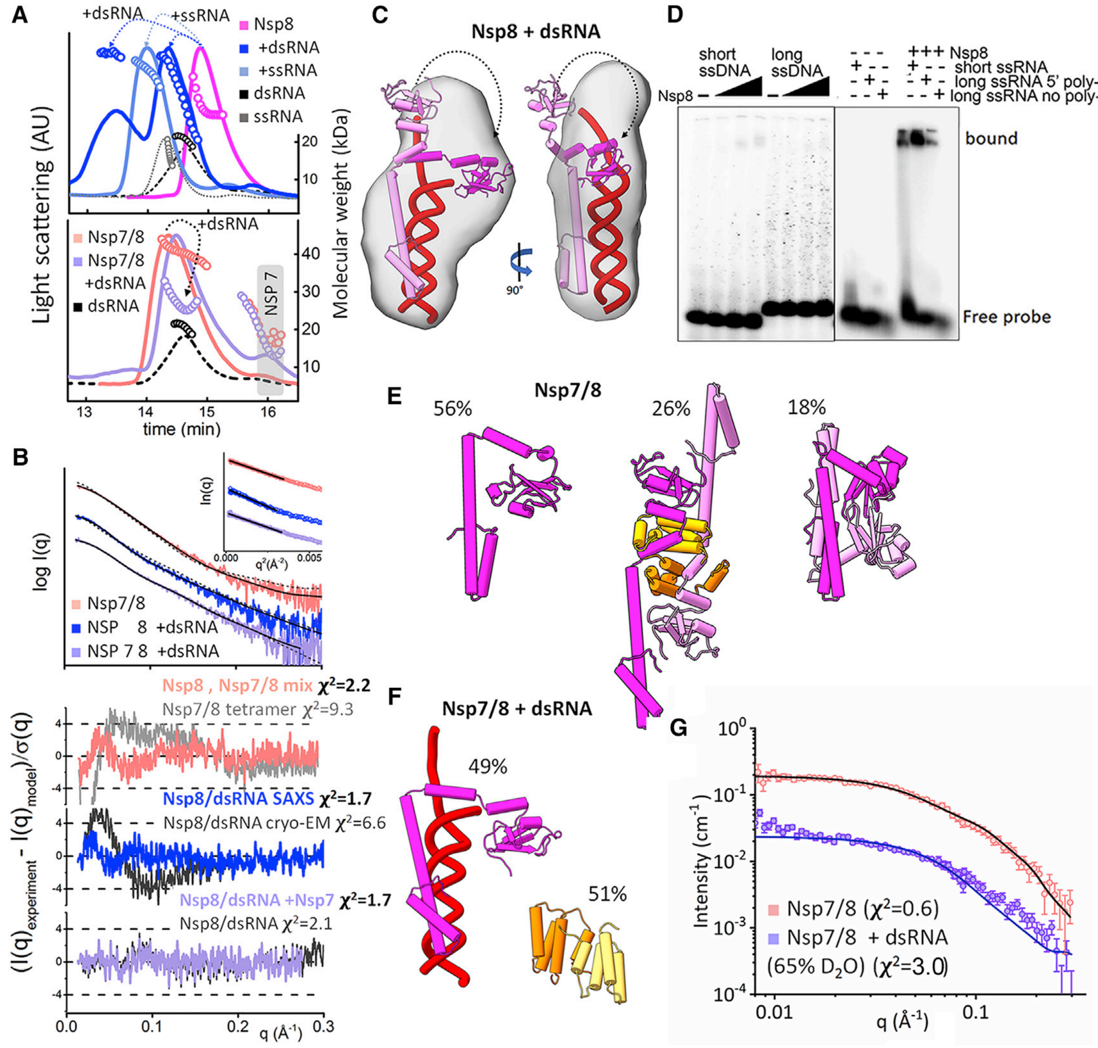

FIGURE 4 Complexation of Nsp8 and Nsp7/8 with the nucleic acid. (*A*) SEC-MALS chromatograms for Nsp8, Nsp8 + dsRNA, Nsp8 + ssRNA, dsRNA, and ssRNA (*top*) and Nsp7/8, Nsp7/8 + dsRNA, and dsRNA (*bottom*) are colored as indicated. Solid lines represent the light scattering in detector units (*left axis*), and symbols represent molecular mass versus elution time (*right axis*). (*B*) Experimental SAXS profiles for Nsp7/8, Nsp8 + dsRNA, and Nsp7/8 + dsRNA collected at the SEC peak shown together with calculated SAXS profiles from best fitting atomic models (*black line*) or alternative model (*dash line*). Guinier plots for experimental SAXS curves are shown in the inset. Residuals of best-fit models (colored as indicated), alternative models (*gray*), and goodness-of-fit values (χ^2) are shown in bottom plot. (*C*) Solution model of Nsp8-dsRNA (*magenta* and RNA in *red*) used in the calculate SAXS profile in (*B*) with overlaid SAXS-based shape. (*D*) Nsp8 EMSA with radio-labeled polynucleotides shows no binding of ssDNA (*right*) and binding of all ssRNA substrates. (*E*) Ensemble of structures that fit Nsp7/8 used in the calculated SAXS profile in (*B*). Mass of each model is indicated. (*F*) The ensemble that fits the SAXS from Nsp7/8 + dsRNA with mass of each model indicated. (*G*) SANS data for the Nsp7/8/RNA complex (*pink circles*) and Nsp7/8/DNA in 65% D_2O (*light blue circles*) were fit by the models shown in (*E*) and (*C*).

Nsp7 (Fig. 1 *A*) has three consecutive α-helices (α1, α2, and α3) forming a three-helical coiled-coil bundle and the C-terminal loop (residues 62–70) that in some structures is followed by a short, not well-defined helix (residues 68–72). The conformation of Nsp8 has previously been described to resemble a golf club (Fig. 1 *A*; (9)). It has an N-terminal α-helix (α1) that starts in our structure at Asp78 because of the truncation by Glu-C, which is highly positively charged for binding RNA. This helix is followed by the second α-helix (α2), which connects through a long loop to a half β-barrel-like domain formed by five antiparallel β strands (β1–5) with a small α-helix (α3) inserted between the first and second strands and another insertion of a long loop that contains two half-turn helices (labeled α4). The C-terminus (193–198) of the Nsp8 is well defined in Nsp7/8B, but not in other forms.

In all of our crystal forms, the heterodimer formed between Nsp7 and 8 places Nsp7 near the half β-barrel-like domain of Nsp8 with the N-terminal helix of Nsp8 extended and pointing away (approximate dimensions of 40 × 40 ×

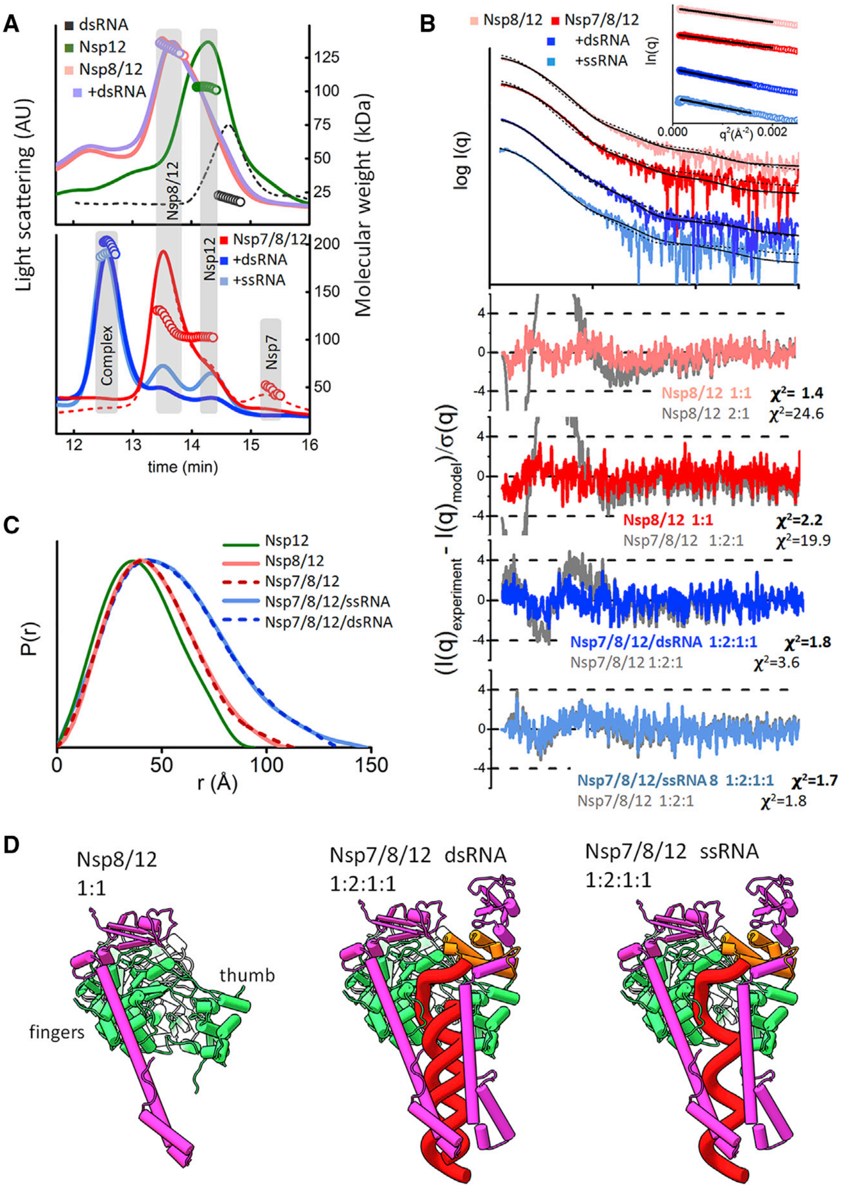

FIGURE 5 RNA-stabilized Nsp7/8/12 complex. (*A*) SEC-MALS chromatograms for Nsp12, Nsp8/12, Nsp8/12 + dsRNA, and dsRNA (*top*) and Nsp7/8/12, Nsp7/8/12 + dsRNA, and Nsp7/8/12 + ssRNA (*bottom*) are colored as indicated. Solid lines represent the light scattering detector units, and symbols represent molecular mass versus elution time. (*B*) Experimental SAXS profiles for Nsp8/12, Nsp7/8/12, Nsp8/12 + dsRNA, and Nsp7/8/12 + ssRNA collected at the SEC peak are shown together with the theoretical SAXS profiles for best fitting models (*black line*) and alternative models (*dash line*). SAXS fits are shown together with the fit residuals for the solution-state model (colored as indicated), alternative model (*gray*), and goodness-of-fit values (χ^2). Guinier plots for experimental SAXS curves are shown in the inset. (*C*) Normalized P(r) function for Nsp12, Nsp8/12, Nsp7/8/12, Nsp7/8/12 + dsRNA, and Nsp7/8/12 + ssRNA. The similarity of P(r) functions between Nsp8/12 and Nsp7/8/12 further confirms the absence of Nsp7 and one Nsp8 in the Nsp7/8/12 mixture. (*D*) Solution-state models for Nsp8/12, Nsp7/8/12 + dsRNA, and Nsp7/8/12 + ssRNA were used to fit experimental data shown in (*B*).

46 Å) (Fig. 1 *A*). The similarity between all heterodimers in all our crystal forms is remarkable, with a root mean square deviation of 0.82 Å for 184 aligned Cα atoms despite different unit cell dimensions, different space groups, and different asymmetric units. These heterodimers are also similar to those from other coronaviruses, for example, RMSD between the heterodimer of Nsp7/8C and that of the Nsp7/8 from SARS-CoV (PDB: 5F22) is 0.91 Å for 184 Cα atoms. These similarities suggest that, once formed, the heterodimer is rigid.

The interface between Nsp7 and Nsp8 is made through hydrophobic contacts between six bundled helices. The helices involved are the two N-terminal helices (α1 and α2) from Nsp8 and four helices from Nsp7. At the C-terminus, the fourth helix in Nsp7 is not well defined, and the loop of residues 66–72 wedges in between the two (α1 and α2) Nsp8 helices to extend the interacting surface. The dimer interface area is 2834 Å^2 with 72% hydrophobic contacts. The key interfacial residues are shown in Fig. 1 *C*.

Our heterodimer structure is in good agreement with the recent cryo-EM structures of Nsp7 and Nsp8 bound to Nsp12 with and without RNA. Using the most complete structure (PDB: 6YYT) from Hillen et al. (10), the superposition has an RMSD of 1.0 Å. There are two Nsp8s in this structure, but only one is in contact with Nsp7. The interactions between Nsp12 and the Nsp7/8 are mostly through the Nsp7 and the region known as the thumb domain (residues 812–932) located at the C-terminus of Nsp12. The Nsp8 monomer interacts with the fingers domain (residues 250–398) of Nsp12 primarily through

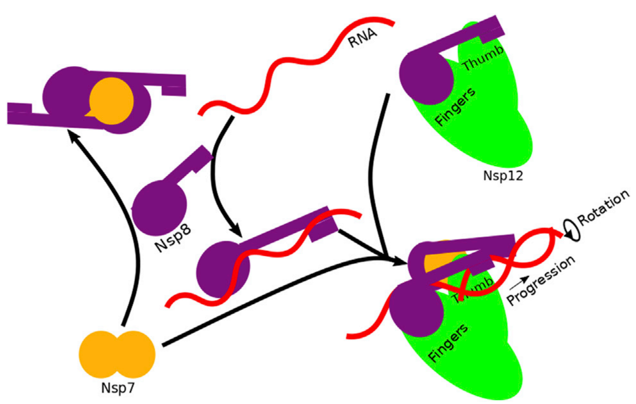

FIGURE 6 Assembly of the RTC components. The solved crystal structure (*top left*) reported in this work exists in a dynamic equilibrium and forms at high concentrations of Nsp7 and Nap8. Nsp7 is dominantly dimeric on its own (*bottom left*), though it can form linear oligomers. Nsp8 alone is in a compact conformation (*center*). However, this conformation becomes extended when RNA or Nsp7 binds in a competitive manner for available binding sites on Nsp8. When all three are combined with a stabilized form of 1:1 Nsp8/12, a very stable Nsp7/8/12 RNA complex forms in a 1:2:1:1 ratio for RNA transcription. The architecture, preferred binding sites for Nsp8 on the fingers domain, and strong binding of RNA by Nsp8 suggest a mechanism in which the Nsp8 on the thumb domain may swap positions with that on the fingers while the one on the fingers progresses with the RNA.

α2, the following loop, and β1 (residues 99–126) and has a significantly different conformation compared to the Nsp7/8 heterodimer. The C-terminal two-thirds of Nsp8 (residues 127–192) is quite similar (overall difference of 1.2 Å over 67 residues). Finally, the location and orientation of the N-terminal helices are different from those in the Nsp7/8 heterodimer. In the cryo-EM structures, the N-terminal helix of Nsp8 makes significant contacts with Nsp12, including the Glu81 region, which perhaps protects Nsp8 from the proteolysis we observed during our crystallographic efforts.

In contrast to previously reported Nsp7/8 structures from other coronaviruses, in all three of our structures, either as in the asymmetric content or symmetry related, a heterotetramer or a dimer of heterodimers is present as an elongated, thick rod shape of 40 × 92 Å (Fig. 1 *B*). The differing crystal forms show only slight variation in the packing of Nsp7/8 heterodimers to form heterotetramers. Comparing the heterotetramers in the asymmetric units, the RMSD is 1.28 Å for 368 aligned Cα atoms. The heterotetramers are formed between the two heterodimers that are related by the noncrystallographic twofold rotational symmetry. In Nsp7/8A, the tetramers are formed by the crystallographic twofold rotational symmetry.

The heterotetramer interface involves a total of six helices: α1 and α2 of Nsp7 and their symmetry mates (twofold rotation) $\alpha 1'$ and $\alpha 2'$ and α1 of Nsp8 and the symmetry mate $\alpha 1'$. The symmetry-related secondary structures are indicated by apostrophes. The tetramer interface is not as extensive as that of the dimer having 1813 Å^2 with 73% being hydrophobic. The residues involved are mostly making hydrophobic contacts with symmetrically related residues. Importantly, a symmetrically related interchain disulfide bond is also found between Cys8 of Nsp7 and its symmetry mate (Fig. 1 *D*).

Among the two crystal structures of Nsp7/8 complexes from SARS-CoV reported, the tetrameric form (PDB: 5F22) is similar to our tetrameric structures. The second form (PDB: 2AHM) of the hexadecameric superstructure of eight each of Nsp7/8 presents a cylindrical ring ~90 Å long and with ~30 Å inner diameter lined with positive charge and could accommodate two and a half turns of RNA duplex (9), suggesting a role as a primase for Nsp12. In the hexadecamer, there are two different heterodimers with two different conformations of Nsp8. The tetramer interface is angularly shifted to propagate a ring-like rather than linear topology. In our tetrameric form, each heterodimer is in near identical conformations.

Assemblies in solution by SAXS and SANS

We demonstrated we could successfully produce an active RTC complex from Nsp7/8/12 using a radioactive-based label extension assay. The extension assay showed the RTC creating duplexed 36 basepaired RNA from a 31 base hairpin substrate where the 5′ overhang had five unpaired bases (Fig. S1). Confident in the relevance of our largest complex, we sought to characterize all potential subcomplexes.

To inform on assembly and conformation in solution, we collected SEC-MALS-SAXS, SANS, and other biochemical data on mixtures of Nsp7, Nsp8, Nsp12, and RNA (Fig. 2). Before experiments, all samples were extensively purified and prepared with the sequences as described in Materials and methods. We used two primary RNA substrates. Our dsRNA has an 8 base overhang and 28 bases of duplex (Fig. S3). Uridine comprises the first four bases of the 5′ overhang. The sequence is analogous to that used in a cryo-EM study (10) differing only in that the strands are continuous rather than containing breaks. The 36-base ssRNA is derived from a long chain of dsRNA and is referred to as long ssRNA.

Each SEC-MALS-SAXS elution contains considerable information (e.g., Fig. 3 *A*). Analysis of the MALS on elution traces (*left axis*, *solid* and *dashed lines*) is used to determine the molecular mass (*right axis*, *circles*). Analyzed SAXS profiles are generated by integrating in regions of constant mass and Rg from SEC-MALS-SAXS (e.g., Fig. 3 *B*) across the main peaks in an elution. Calculations performed on an atomic model generate a SAXS curve (using FOXS (40,41) that can be compared to the experiment using χ^2 or residual as metric of agreement. If agreement is poor (e.g., Fig. 3 *B*, *lower plots*), then the models are adjusted by molecular dynamics in BILBOMD (39) until an adequate fit and residual are attained. In cases

of conformational flexibility, a single model will not be sufficient to fit the data, and an ensemble of models must be used (e.g., Fig. 3, *C–E*). All scattering measurements were made in slightly reducing conditions, mimicking intracellular space, to prevent disulfide bond formation. Using the isotope-based masking properties of SANS, proteins were also examined for conformational change when interacting with masked components in complexes (e.g., Fig. 3 *F*). Global parameters extracted from these SAXS and SANS profiles can be found in Table S2. Below, we organize our results in order of increasing mixture complexity.

Nsp7 forms multimers in solution and does not bind RNA

The SEC-MALS-SAXS of Nsp7 reveals that Nsp7 forms multimers with a different organization than that found in our crystal structure of Nsp7/8 (Figs. 2 and 3). From the dominant late peak, MALS-SAXS measurements are consistent with a well-folded dimer with a mass of 17 kDa (monomeric mass 9 kDa) and a maximal dimension (Dmax) of 60 Å (Fig. S2 *B*). The primary peak in the MALS-based chromatogram is preceded with larger multimer of Nsp7 (Fig. 3 *A*). The presence of larger multimers of Nsp7 are important to note because they complicate the analysis of complexes of Nsp7 with Nsp8, Nsp12, and RNA described further. The Nsp7 multimers can erroneously lead to an interpretation that Nsp7 is part of larger complexes, as homo-oligomers of Nsp7 coelute with larger macromolecular complexes.

Indicative of a different assembly in solution, SAXS calculated from the Nsp7 dimer taken from the Nsp7/8C crystal structure disagreed with the measured SAXS data ($\chi^2 = 40$). Poor agreement remained ($\chi^2 = 7.5$, Fig. 3 *B*) even after optimized remodeling of the C-terminal helix (69–84) that is solvent exposed in the absence of Nsp8. Furthermore, the shape calculated from our SAXS data by DAMMIF (50) is more elongated than the dimer taken from the Nsp7/8 structure (Fig. 3 *C*). Assuming the Nsp7 monomer fold visualized in our crystal structure, orientations of Nsp7 in the dimer that fitted the SAXS data were found using the SAXS guided computational docking program FOXDOCK (42). The best docking results were from head-to-tail orientations of Nsp7 monomers ($\chi^2 = 1.6$) (Fig. 3 *C*). The head-to-tail interface may also allow the formation of the larger oligomers observed in MALS chromatograms (Fig. S2 *A*). These oligomers were further analyzed and were consistent with an elongated, rather than globular, form of Nsp7 dimers (Fig. S2 *B*).

Nsp7 did not complex with RNA by SEC-MALS-SAXS analysis. Measurements of mixtures of Nsp7 with RNA resulted in SAXS and masses consistent with the two eluting separately (Fig. S2 *A*). These results were supported by comparisons to measurements on the RNA run separately (Fig. S3, *A* and *B*).

Nsp8 forms concentration-dependent multimers and has an alternate conformation when monomeric

Nsp8 forms concentration-dependent oligomers in solution. SAXS profiles collected in high-throughput mode at three concentrations (2.5, 5, 10 mg/mL) varied more significantly than could be explained by a concentration-dependent long-range attraction, indicating complexation between monomeric units (Fig. S4 *A*). Similarly, SEC-MALS-SAXS from Nsp8 showed an asymmetric elution peak, with the front of the peak having a larger molecular mass than the later-eluting material that matched the monomeric mass of Nsp8 (Fig. 3 *A*).

As a monomer, Nsp8 adopts a different conformation to that observed in atomically resolved complexes. The conformations of Nsp8 monomers found in the Nsp7/8 crystal structure (PDB: 3UB0 (49) and Nsp7/8/12 cryo-EM structure (PDB: 6YYT (10) did not match the SEC-SAXS data from the elution region containing the monomers ($\chi^2 =$ 40, Fig. 3 *B*). In these Nsp7/8 or Nsp7/8/12/RNA complex structures, the Nsp8 N-terminal helix-turn-helix bundle (1–100) adopts various extended conformations. To find a conformation that fits the SAXS data, we employed conformational sampling of the N-terminal helix bundle. The flexible tethers between the head and two distinct helix regions (1–82 and 86–100) were identified by structural comparison of the two Nsp8 conformers taken from the Nsp7/8/12/RNA complex (Fig. 3 *D*; (10)). Despite providing a nearly exhaustive search of extended and compact conformations to select an ensemble, an excellent fit to the SAXS data (Fig. 3 *B*) required a compact configuration with the head domain in close proximity to the N-terminal region of the helix-turn-helix region (Figs. 3 *D* and S4). The folding back of the structure may be driven by a polar interaction between a positively charged segment of the long connecting helix with a negatively charged portion of the beta-barrel containing domain (head), as calculated by PDB2PQR/Adaptive Poisson-Boltzmann Solver (Fig. S5; (51,52)). These regions could be further driving the multimerization, as shown in the crystal structure of the Nsp7/8 complex of feline coronavirus (PDB: 3UB0) (Fig. S4 *C*; (49)).

To fit higher concentrations of Nsp8, we created a model for dimers that combines our monomeric model with dimerization contacts of Nsp8 found in an analog crystal structure (49). A mixture of the dimer and monomer models fitted the SAXS data for Nsp8 at a 2.5 mg/mL concentration (Fig. S4 *A*) and the SAXS data from the leading edge of the SEC major elution peak. At our higher concentrations (5 and 10 mg/mL), the SAXS profiles could not be fitted using a monomer-dimer mixture and required a model with longer

maximal distances, as shown by Dmax values determined from the P(r) function (Fig. S4 *B*). A small contribution of a larger tetrameric state built based on the Nsp7/8 structure (PDB: 2AHM) (9) was necessary to fit SAXS data for the two highest protein concentrations (Fig. S4 *C*).

Nsp12

Nsp12 alone expresses poorly and has low solubility. This suggests that Nsp12 is unlikely to function alone and must form heteromeric complexes for stability. The maximal concentrations obtained were 2.5 mg/mL, above which aggregation of the protein was evident. The MALS and SAXS mass is consistent with a monomer (Figs. 2 and 3 *A*). The interpretable SAXS signals below $q < 0.2$ Å^{-1} were in good agreement ($\chi^2 = 1.4$, Fig. 3 *B*) with the available atomic model of Nsp12 (Fig. 3 *E*; (10)). The Rg of the measured profile was 31.1 ± 0.5 Å, whereas that calculated from the model with added missing N-terminal region (1–31, 50–77) is 31.6 Å (Table S2).

Nsp8 binds RNA, but not DNA

We characterized the interaction of Nsp8 with dsRNA (Figs. 4 and S3) and dsDNA (Fig. S5). The dsDNA is analogous in sequence to dsRNA. Nsp8 did not form a complex with dsDNA because both the Nsp8 and dsDNA elute separately at elution times consistent with each run independently. In contrast, Nsp8 forms two types of complexes with the dsRNA, contributing to two well-separated peaks (Fig. 4 *A*). The first elution peak (13.5 min after injection) is consistent with a 1:1 Nsp8 dsRNA complex, whereas in the second Nsp8/dsRNA peak (14.3 min), the mass decreases rapidly across the elution profile, indicating the presence of a heterogeneous mixture of Nsp8 and free dsRNA.

We further tested whether Nsp8 binding to DNA or RNA is length or sequence dependent. Nsp8 did not bind either long (36 base) or short (28 base) ssDNA in our SEC-MALS-SAXS analysis or in an electrophoretic mobility shift assay (EMSA) (Fig. 4 *D*). Nsp8 did bind the long ssRNA, but not the short ssRNA, at high affinity. The mixture of Nsp8 with long ssRNA shows only one distinct peak, consistent with the 1:1 complex as judged by determining the molecular mass and SAXS parameters (Figs. 2 and 4 *A*). We further tested binding of long ssRNA, short ssRNA, and the long ssRNA without the poly-U 5′ end by EMSA. Nsp8 showed evidence of binding all three; however, the affinities are difficult to assess with EMSA bands. The EMSA bands with ssRNA are split. On the same mixtures from SEC-MALS-SAXS, the mass through the peak drops rapidly (Fig. 4 *A*) suggestive that multiple Nsp8s could bind the same ssRNA. These results demonstrate Nsp8 discriminates between RNA and DNA. SEC-MALS-SAXS showed a preference for longer ssRNA over the shorter.

We found Nsp8/dsRNA models that fit the SAXS data from the highest quality signal in the constant mass region of the main elution peak (Fig. 4, *A* and *B*). This model is supported from the elongated shape determined from SAXS by DAMMIF (50) and consists of Nsp8 binding dsRNA along their long axes (Fig. 4 *C*). In our rigid-body modeling using BILBOMD (39), we preserved the Nsp8-dsRNA interaction at the N-terminal Nsp8 helix bundle that contains positively charge patches (Fig. S5) and sampled conformations of C-terminal head domain. An excellent fit ($\chi^2 = 1.7$, Fig. 4 *B*) was found with the Nsp8 head domain wrapping around the RNA capping the terminal region of the RNA molecule (Fig. 4 *C*), providing a structural model for Nsp8 RNA binding.

The Nsp7/8 complex is transient in solution

The shape of the elution peak from coexpressed and affinity-tag-purified Nsp7/8 is consistent with a transient complex composed of oligomers and free proteins. The SEC-MALS-SAXS elution is dominated by an asymmetric peak with a long tailing shoulder region (Fig. 4 *A*, *lower*). The mass at the leading edge by MALS is 45 kDa, and SAXS is ~42 kDa, which is smaller than the tetramer's expected molecular mass (63 kDa) (Fig. 2). Furthermore, a well-separated peak observed at later elution times is consistent with that observed from Nsp7 dimers, further supporting a transient Nsp7/8 complex (Fig. 4 *A*).

To fit the SAXS data from the dominant peak, we used an ensemble of models. The ensemble pool included a dimer and heterotetramer of Nsp7/8 built from our crystal structure in which Nsp8 is in an extended conformation to include a helix and N-terminal bundle. We also included a monomeric Nsp8 with various conformations of the N-terminal helix bundle and an Nsp8 dimer with open and compact head domain (see Fig. S4 *C*). The data are the best fit by a mixture of 26% Nsp7/8 heterotetramer, 56% monomeric Nsp8, and 18% Nsp8 dimer with a compact head domain ($\chi^2 = 2.2$, Fig. 4, *B* and *E*). The Nsp7/8 heterotetramer observed in our crystal structures is a minor population at these concentrations.

A similar analysis was performed on SANS data collected from the same complex differing by collection at high concentration and in a fully equilibrated sample (non-SEC). The mass determined from SANS was also slightly lower (56 ± 3 kDa) than the Nsp7/8 heterotetramer. The Nsp7/8 heterotetramer crystal structure did not fit the SAXS data. However, using OLIGOMER, a good fit ($\chi^2 < 1.0$) was obtained with a mixture of 88% Nsp7/8 heterotetramer and 12% Nsp8 monomer. This supports the SEC-SAXS analysis that shows the dynamic and concentration-dependent nature of the interaction between Nsp7 and Nsp8.

Furthermore, to test our findings that Nsp7 induces structural change in Nsp8 relative to its monomeric state, we performed contrast-matching SANS experiments with the

Nsp7/8 complex (Fig. 3 *F*). Deuterated Nsp7 (dNsp7) was copurified with protiated Nsp8 to form dNsp7-Nsp8. Using the contrast match point of dNsp7 (90% D_2O), we single out the scattering from complexed Nsp8. Fig. 3 *F* compares the P(r) of dNsp7-Nsp8 against a solution of Nsp8 without Nsp7. The P(r) from the complex dNsp7-Nsp8 has a bimodal shape, indicating two well-separated protein densities, whereas the P(r) of Nsp8 alone is more centralized to one main distance. The change verifies Nsp7 dramatically modifies Nsp8 conformation or assembly.

RNA competes with Nsp7 to interact with Nsp8

In contradiction to several proposed pathways, our SEC-MALS-SAXS experiments do not support a stable ternary Nsp7/8 dsRNA complex. Quite the opposite; our results indicate a competition between Nsp7 and dsRNA for Nsp8 binding. The elution peak from mixtures of Nsp7/8 with RNA is shifted in agreement with the smaller measured mass (MALS) from 45 kDa for Nsp7/8 to ~35 kDa for Nsp7/8 + dsRNA (Fig. 4 *A*, *lower*). The secondary SEC peak for free Nsp7 is more pronounced and well separated (Fig. 4 *A*), suggesting the uncoupling of Nsp7 from the complex.

Fitting of the SAXS data provides further evidence that dsRNA destabilized the Nsp7/8 interface. From the pool of models that includes conformers of Nsp8, Nsp7 monomer and dimer, Nsp7/8 dimer and tetramer, and multiple conformers of Nsp8-dsRNA, the best fit was obtained with the Nsp8-dsRNA complex ($\chi^2 = 1.7$, Fig. 4 *B*) described above. The multistate model selection that contains Nsp7 dimer further improved the SAXS fit ($\chi^2 = 1.6$, Fig. 3, *B* and *D*) and suggested transient binding of dsRNA that led to the decoupling of Nsp7/8 and presence of free Nsp7 across the peak. The presence of Nsp7 dimers also explains the smaller Rg and mass determined by SAXS or MALS relatively to the values measured for the Nsp8/dsRNA complex (Rg 28.9 Å vs. 32.2 Å, $Mass_{MALS}$ 32 vs. 45 kDa, and $Mass_{SAXS}$ 30 vs. 45 kDa) (Figs. 2 and 4 *A*; Table S2).

Contrast-matching SANS experiments, masking the RNA signal in 65% D_2O, were also performed on Nsp7/8 dsRNA mixtures. The contrast-matched SANS profile is best fitted ($\chi^2 = 3.0$) with the Nsp8 monomer (Fig. 4 *G*), supporting the dissociation of the Nsp7/8 complex observed in the SEC-SAXS. Altogether, the combined SANS and SAXS indicate that RNA alters the interactions found in our crystal structure of Nsp7/8 alone, leading to the formation of a smaller Nsp8-dsRNA complex and disassociation of Nsp7 from the complex (Fig. 4 *F*).

Without Nsp7, Nsp12 recruits one Nsp8 to its finger region and does not bind RNA

The mass across the center of the SEC-MALS-SAXS main peak from coexpressed Nsp8 and 12 is 130 kDa, in agreement with the mass of an Nsp8/12 1:1 complex (Fig. 5 *A*). The main peak trails into a mass (110 kDa) consistent with a free Nsp12. The broadening of the elution peak indicates disassociation of Nsp8 from Nsp12. The central portion of the main peak was merged to obtain a SAXS profile sufficient to be modeled in detail.

The Nsp8/12 SAXS profile was fitted with a pool of the models containing various conformers of Nsp8 (Fig. 3 *D*), Nsp12 (Fig. 3 *E*), and three models of the Nsp8/12 complex with Nsp8 located at the fingers, thumb region, or both (10). The pool also contained alternative models of the Nsp8/12 complex with an extended Nsp8-helix bundle region. The best fit ($\chi^2 = 1.4$) was obtained with a single model of Nsp8/12 containing one Nsp8 bound to the finger region (Fig. 5 *D*). This model matches experimental SAXS better than the Nsp8/12 model with both Nsp8 bound ($\chi^2 = 24.6$) (Fig. 5 *B*) and agrees with the determined mass (Fig. 2). We attempted to improve the fit with the multistate models, but the single conformation remained the best fit.

These findings suggest Nsp12 recruits only one Nsp8 to the finger region and raises the question of whether Nsp7, RNA, or both are required to stabilize the Nsp8 interaction at the thumb region. Therefore, we investigated Nsp7/8/12 assembly in solution and the interactions of Nsp8/12 with RNAs.

Nsp7, Nsp8, and Nsp12 were coexpressed and purified for SEC-MALS-SAXS analysis. The first SEC trace, before SAXS measurements, shows a split peak and tail with a significant peak from Nsp7 at later elution times, already suggesting transient dissociation of Nsp7 from the Nsp8/12 complex (Fig. S7). The early fractions were subsequently analyzed (and consequently purified by SEC a second time) by SEC-MALS-SAXS. The mass of the first elution peak (132 kDa) agrees with the Nsp8/12 1:1 complex. The elution time of the peak (Fig. 5 *A*), determined mass, SAXS parameters (Fig. 2), and calculated P(r) functions (Fig. 5 *C*) from this first peak are identical to those reported above from Nsp8/12 when no Nsp7 was present. Furthermore, the Nsp8/12 1:1 model (Fig. 4 *D*) gives an excellent match to the experimental SAXS curve ($\chi^2 = 2.2$) (Fig. 5 *B*) that is distinct from other potential models, including an Nsp7/8/12 1:2:1 complex with a significantly worse fit ($\chi^2 = 19.9$). The trailing shoulder and peak from the elution trace is consistent with Nsp12 alone and an Nsp7 dimer (Fig. 5 *A*), further supporting the dissociation of Nsp7 from the Nsp8/12 1:1 complex.

Mixing an excess of RNA with Nsp8/12 does not lead to a high-affinity complex. Identical elution time and MALS-measured molecular mass (~130 kDa) of the early elution peak of Nsp8/12 + dsRNA (Fig. 5 *A*), Nsp8/12 + ssRNA (Fig. S7), and Nsp8/12 alone clearly show the absence of an Nsp8/12 interaction with RNA. The low or nonexistent binding of RNA by Nsp8/12 is surprising because Nsp8 alone binds RNA strongly. We find that Nsp8/12 does not stably interact with Nsp7 or RNA on its own; rather, Nsp7

needs to interact with Nsp8 to form the complete SARS-Cov-2 polymerase machinery.

Nsp7/8/12 requires RNA for stability

Adding dsRNA or ssRNA stabilizes the Nsp7/8/12/RNA within a 1:2:1:1 complex as visualized in cryo-EM atomic-resolution structures (10). Thoroughly mixing near equivalent molar ratios of dsRNA or ssRNA with the initially purified Nsp7/8/12 complex, assuming a 1:2:1 complex, yields a sharp and near symmetric SEC elution peak (Figs. 5 *A* and S6). The shift in the SEC peak and determined mass (~190 kDa) suggests the formation of Nsp7/8/12/RNA in 1:2:1:1 complex for both complexes, which agrees with the theoretical mass of 1:2:1:1 complexes with 181 kDa for Nsp7/8/12/ssRNA and 172 kDa Nsp7/8/12/dsRNA (Fig. 2). The experimental SAXS profiles (Fig. 5 *B*) for both complexes are consistent with models built from the cryo-EM structure (10) by adding all missing regions in the protein and RNA region and removing a shorter RNA strand for Nsp7/8/12/ssRNA complex (Fig. 5 *D*). This agreement further suggests that both Nsp8 copies remain pointed in the direction of RNA polymerization rather than flexing in alternate conformations.

DISCUSSION

Our overall aim was to further develop a comprehensive understanding of the SARS-CoV-2 RTC and identify strategies for inhibiting its assembly and mechanism. To this end, we have integrated information from available cryo-EM and x-ray crystallography structures with the results reported above, which include biochemical assays, atomic-resolution structures of Nsp7/8, and 20 SAXS and SANS experiments on components of the RTC. Our crystal structures, those resolved by others, and cryo-EM results provide a basis to interpret our solution scattering studies that probe the transient and plastic properties of the assembly, which are difficult to attain with static structures alone. The highly evolved and dynamic nature of this essential complex greatly enables SARS-CoV-2; however, these same properties also provide many avenues for disruption and inhibition. Summarizing our results, we have arrived at the following insights.

At high concentrations, as found in our crystal structure, Nsp7/8 forms a tetrameric structure composed of a dimer of heterodimers. However, this structure is transient, and at low concentrations, the proteins disassociate into mixtures of the individual components and smaller complexes. Nsp7 primarily exists as a dimer, but longer oligomeric structures are also present, which can be confounding to size-based purification methods because Nsp7 will be present in one oligomeric form or another at separate elution times. Without being bound to Nsp7, Nsp8 adopts a compact conformation not yet observed in crystal structures and will oligomerize into flat aggregates at high concentrations. Nsp8 will readily bind dsRNA and ssRNA, but not ssDNA. Nsp12 alone is unstable and prone to aggregation even at low concentrations (>2.5 mg/mL). Binding of Nsp8 markedly improves stability.

The atomic coordinates from our crystal structure and our solution scattering results inform on the consequences of Nsp7's overall hydrophobic character. The eight hydrophobic amino acids make up 45% of the protein, including 18% leucine and 11% valine. The solvent-excluded interface made between Nsp7 and Nsp8 is 57% hydrophobic. In all of our structural studies, Nsp7 is found in complex with Nsp8 and Nsp12, or it has the propensity to form chains of Nsp7 oligomers on its own. A further consequence of its hydrophobic nature is its ability to disrupt the hydrophobic cores of the other proteins and modify their conformational states as exemplified by its interaction with Nsp8. The 2:2 Nsp7/8 we observe in our crystal structure may be a storage form of the complex when little RNA is available. Nsp7's ability to compete with RNA, a hydrophilic and charged molecule, for Nsp8 binding is not intuitive. One mechanistic possibility is that the binding of Nsp7 reduces the footprint Nsp8 has to bind RNA and serves to offload RNA from Nsp8. This mechanism could be fundamental to the operation or assembly of the RTC and provides a rational for expressing Nsp7 as a separate peptide rather than as part of Nsp8 or Nsp12.

We observed two stabilized structures with Nsp12. In the absence of its binding partners, Nsp12 alone seems inherently unstable, which limited our ability to create complexes by mixing the purified proteins. However, by coexpressing the individual proteins, reasonable yields of the complexes could be attained. A stable 1:1 structure forms between Nsp8 and Nsp12, where an Nsp8 sits on the fingers of Nsp12. This complex, however, is not capable of binding the RNAs we tested. Although cryo-EM has partially visualized an Nsp7/8/12 structure without RNA, the sample had to be supplemented with significant concentrations of Nsp7 and 8 for resolving these structures. In our hands and at the concentrations we made our measurements (1–5 mg/mL), Nsp7/8/12 does not form a stable structure until RNA is present. Once RNA is present, a very stable 1:2:1:1 Nsp7/8/12/RNA complex is formed both with dsRNA and ssRNA.

In all the most complete cryo-EM structures of the Nsp7/8/12/RNA with and without Nsp13 or Nsp9, the structure and position of Nsp7 and 8 are remarkably similar. This similarity suggests Nsp7 and 8 are static components during replication. Their role is thought to be threefold: to help close the complex once RNA is bound, to guide RNA upon exit, and to stabilize contacts with Nsp13 and other proteins. As noted with the first complete structure, Nsp7 and 8 confer processivity (10). The orientations of the helical extensions on Nsp8 remain uniquely observed in the

RTC and may function similar to sliding clamps in DNA replication. Nsp8's structure is suited to preforming or further enhancing a straightened RNA with positively charged RNA binding patches spaced to coincide with an RNA double helix.

However, for these roles, expression of Nsp7 and Nsp8 as independent polypeptides, rather than as one protein or as part of Nsp12, seems inefficient. Nsp7 and Nsp8 have been postulated to take part in other viral activities, which may justify their independence. Exploring other potential avenues, we build upon cryo-EM and our results to suggest more dynamic roles for the components. We propose that Nsp8 is involved in recognizing ssRNA over ssDNA and guiding ssRNA to or from the RTC complex. Our observation that Nsp8 adopts a different conformation alone relative to bound states suggests Nsp8 is a dynamic component of the macromolecular machine. Based on the exclusive binding of either Nsp7 or RNA by Nsp8, but not both simultaneously, we propose that Nsp7 promotes the release of RNA from Nsp8 once it has been guided to the RTC by disrupting Nsp8's head domain. The stability of the Nsp8 on the fingers domain is intriguing and certainly serves to further stabilize the RTC's RNA binding. However, we have shown that the fingers-bound Nsp8 is not sufficient to bind RNA and Nsp7 is necessary.

The binding and position of two Nsp8s could play a role in transitions that occur during RNA transcription. RNA rotates and progresses during polymerization. The compacting and expanding properties of Nsp8 could be part of a retraction mechanism in which, once the N-terminus of Nsp8 is overstretched, it releases and rebinds closer to Nsp12, where newly synthesized and duplexed RNA is emerging. Alternatively, the combination of RNA rotation and progression may pull the fingers-bound Nsp8 off of Nsp12, and its vacant spot may be taken up by the Nsp8 bound to the thumb (Fig. 6). The energetics could be favorable because there is a 1:1 trade from an Nsp8 in a slightly destabilized configuration through rotation and progression of the RNA. The now-vacant spot on the thumb domain would then be occupied by free Nsp8, or perhaps both Nsp7 and 8 are replaced. The transition would also allow Nsp12 to reset or release its grasp on RNA because binding of RNA by Nsp12 is dependent on Nsp7. The available structures of Nsp7/8/12/13/RNA do not prohibit such an exchange and rather suggest that Nsp13 could also be part of the transition, with the template strand eventually released by the Nsp13 helicase and grabbed by a new one bound at the thumb as well.

The possible mechanistic roles of the components in the RTC described above (Fig. 6), require significant further investigation. In addition to the continued clarity provided by high-resolution cryo-EM and crystallographic structures, further solution- and cell-based studies to examine the dynamics of RTC in its many contexts will be helpful. For example, RNA transcription and polymerization appear to occur in double-membrane vesicles, where the concentrations of the components may be manipulated. We observed several concentration-dependent assemblies in our studies. Additionally, this study was conducted with a limited and specific set of RNAs that may bias our findings. Further studies with a variety of RNA substrates are necessary.

CONCLUSIONS

In our study, we systematically examined components and complexes relevant to the RTC of SARS-CoV-2. We were able to crystalize Nsp7/8, which provides us atomic-resolution details on the interactions between the two components. We have uncovered conformations and identified stable complexes and, to some extent most interestingly, transient complexes. Our results are relevant for a comprehensive understanding of the RTC mechanism and suggest strategies for the disruption of assembly. Developing a further mechanistic understanding will provide insight into how current drugs inhibit the RTC and other potential ways to interfere with the complex.

SUPPORTING MATERIAL

Supporting material can be found online at https://doi.org/10.1016/j.bpj.2021.06.006.

AUTHOR CONTRIBUTIONS

G.L.H. wrote the draft, organized experiments, analyzed structures, and is a corresponding author. M.H. organized experiments, analyzed SAXS and MALS data, analyzed structures, prepared SAXS figures and helped write the manuscript. D.J.R. collected SEC-MALS-SAXS data. J.C.B. helped prepare SAXS samples and checked on reduction state of buffers. S.E.T. helped organize experiments and write the manuscript. A.H.S. performed RNA binding assays. J.W. performed RNA extension assay. M.W. purified and crystallized proteins and determined and refined crystal structures. R.J. cloned genes, expressed and coexpressed proteins, and purified complexes. Y.K. collected data and solved and refined structures. A.J. initiated the project and coordinated efforts between sites. W.L. performed SANS experiments and data analysis and helped to write the manuscript. Q.Z. developed expression and purification protocols for deuterated complexes. K.L.W. developed deuteration protocol for individual proteins for SANS. Y.F. expressed and purified protiated Nsp7/8 complex. S.V.P. supported SANS experiments. H.M.O. led the ORNL effort, performed sample preparation for SANS experiments and data analysis, and helped to write the manuscript.

ACKNOWLEDGMENTS

Inspiration for the finalizing of this work came from the story shared by Lawrence Riley's family, representing the hardships faced throughout this pandemic.

Research was supported by the U.S. Department of Energy (DOE) Office of Science through the National Virtual Biotechnology Laboratory, a consortium of DOE national laboratories focused on response to coronavirus disease 2019, with funding provided by the Coronavirus CARES Act. The Advanced Light Source is a DOE Office of Science user facility run under contract No. DE-AC02-05CH11231 with the SAXS SIBYLS

(12.3.1) beamline supported by the DOE Biological and Environmental Research Integrated Diffraction Analysis Technologies (DOE-BER-IDAT) program, National Cancer Institute Structural Biology of DNA Repair (NCI-SBDR) P01 CA092584, National Institute for General Medical Sciences ALS-ENABLE (P30 GM124169) and United States-Israel Binational Science Foundation (BSF) Grant 2016070. J.W. was supported by the Hatch program of the National Institute of Food and Agriculture, U.S. Department of Agriculture. Bio-SANS is supported by Oak Ridge National Laboratory's (ORNL) Center for Structural Molecular Biology funded by the DOE-BER. EQ-SANS is supported by the Scientific User Facilities Division, DOE Basic Energy Science (DOE-BES). Neutron scattering experiments were performed using the High Flux Isotope Reactor and Spallation Neutron Source, a DOE Office of Science User Facilities operated by ORNL. Preliminary protein expression and purification studies were supported by the ORNL Laboratory Directed Research and Development Program. Further funding for this research was provided by the National Institute of Allergy and Infectious Diseases, National Institutes of Health, Department of Health and Human Services, under Contract HHSN272201700060C. The use of the Structural Biology Center (SBC) beamlines at the Advanced Photon Source is supported by DOE Office of Science and operated for the DOE Office of Science by Argonne National Laboratory under Contract No. DE-AC02-06CH11357.

REFERENCES

1. Yin, W., C. Mao, …, H. E. Xu. 2020. Structural basis for inhibition of the RNA-dependent RNA polymerase from SARS-CoV-2 by remdesivir. *Science*. 368:1499–1504.
2. Kim, D., J. Y. Lee, …, H. Chang. 2020. The architecture of SARS-CoV-2 transcriptome. *Cell*. 181:914–921.e10.
3. Ahn, D. G., J. K. Choi, …, J. W. Oh. 2012. Biochemical characterization of a recombinant SARS coronavirus nsp12 RNA-dependent RNA polymerase capable of copying viral RNA templates. *Arch. Virol.* 157:2095–2104.
4. Gao, Y., L. Yan, …, Z. Rao. 2020. Structure of the RNA-dependent RNA polymerase from COVID-19 virus. *Science*. 368:779–782.
5. Subissi, L., C. C. Posthuma, …, I. Imbert. 2014. One severe acute respiratory syndrome coronavirus protein complex integrates processive RNA polymerase and exonuclease activities. *Proc. Natl. Acad. Sci. USA*. 111:E3900–E3909.
6. Snijder, E. J., E. Decroly, and J. Ziebuhr. 2016. The nonstructural proteins directing coronavirus RNA synthesis and processing. *Adv. Virus Res.* 96:59–126.
7. Tvarogová, J., R. Madhugiri, …, J. Ziebuhr. 2019. Identification and characterization of a human coronavirus 229E nonstructural protein 8-associated RNA 3′-terminal adenylyltransferase activity. *J. Virol.* 93:e00291-19.
8. Banerjee, A. K., M. R. Blanco, …, M. Guttman. 2020. SARS-CoV-2 disrupts splicing, translation, and protein trafficking to suppress host defenses. *Cell*. 183:1325–1339.e21.
9. Zhai, Y., F. Sun, …, Z. Rao. 2005. Insights into SARS-CoV transcription and replication from the structure of the nsp7-nsp8 hexadecamer. *Nat. Struct. Mol. Biol.* 12:980–986.
10. Hillen, H. S., G. Kokic, …, P. Cramer. 2020. Structure of replicating SARS-CoV-2 polymerase. *Nature*. 584:154–156.
11. Peng, Q., R. Peng, …, Y. Shi. 2020. Structural and biochemical characterization of the nsp12-nsp7-nsp8 core polymerase complex from SARS-CoV-2. *Cell Rep.* 31:107774.
12. Yan, L., Y. Zhang, …, Z. Lou. 2020. Architecture of a SARS-CoV-2 mini replication and transcription complex. *Nat. Commun.* 11:5874.
13. Chen, J., B. Malone, …, E. A. Campbell. 2020. Structural basis for helicase-polymerase coupling in the SARS-CoV-2 replication-transcription complex. *Cell*. 182:1560–1573.e13.
14. Yan, L., J. Ge, …, Z. Lou. 2021. Cryo-EM structure of an extended SARS-CoV-2 replication and transcription complex reveals an intermediate state in cap synthesis. *Cell*. 184:184–193.e10.
15. Konkolova, E., M. Klima, …, E. Boura. 2020. Structural analysis of the putative SARS-CoV-2 primase complex. *J. Struct. Biol.* 211:107548.
16. Aslanidis, C., and P. J. de Jong. 1990. Ligation-independent cloning of PCR products (LIC-PCR). *Nucleic Acids Res.* 18:6069–6074.
17. Marley, J., M. Lu, and C. Bracken. 2001. A method for efficient isotopic labeling of recombinant proteins. *J. Biomol. NMR*. 20:71–75.
18. Sherlin, L. D., T. L. Bullock, …, S. A. Scaringe. 2001. Chemical and enzymatic synthesis of tRNAs for high-throughput crystallization. *RNA*. 7:1671–1678.
19. Minor, W., M. Cymborowski, …, M. Chruszcz. 2006. HKL-3000: the integration of data reduction and structure solution–from diffraction images to an initial model in minutes. *Acta Crystallogr. D Biol. Crystallogr.* 62:859–866.
20. French, S., and K. Wilson. 1978. Treatment of negative intensity observations. *Acta Crystallogr. A*. 34:517–525.
21. Padilla, J. E., and T. O. Yeates. 2003. A statistic for local intensity differences: robustness to anisotropy and pseudo-centering and utility for detecting twinning. *Acta Crystallogr. D Biol. Crystallogr.* 59:1124–1130.
22. Winn, M. D., C. C. Ballard, …, K. S. Wilson. 2011. Overview of the CCP4 suite and current developments. *Acta Crystallogr. D Biol. Crystallogr.* 67:235–242.
23. Vagin, A., and A. Teplyakov. 2010. Molecular replacement with MOLREP. *Acta Crystallogr. D Biol. Crystallogr.* 66:22–25.
24. Murshudov, G. N., A. A. Vagin, and E. J. Dodson. 1997. Refinement of macromolecular structures by the maximum-likelihood method. *Acta Crystallogr. D Biol. Crystallogr.* 53:240–255.
25. Adams, P. D., P. V. Afonine, …, P. H. Zwart. 2010. PHENIX: a comprehensive Python-based system for macromolecular structure solution. *Acta Crystallogr. D Biol. Crystallogr.* 66:213–221.
26. Laskowski, R. A., M. W. MacArthur, …, J. M. Thornton. 1993. PROCHECK: a program to check the stereochemical quality of protein structures. *J. Appl. Cryst.* 26:283–291.
27. Hura, G. L., A. L. Menon, …, J. A. Tainer. 2009. Robust, high-throughput solution structural analyses by small angle X-ray scattering (SAXS). *Nat. Methods*. 6:606–612.
28. Classen, S., G. L. Hura, …, J. A. Tainer. 2013. Implementation and performance of SIBYLS: a dual endstation small-angle X-ray scattering and macromolecular crystallography beamline at the advanced light source. *J. Appl. Cryst.* 46:1–13.
29. Knott, G. J., B. F. Cress, …, J. A. Doudna. 2019. Structural basis for AcrVA4 inhibition of specific CRISPR-Cas12a. *eLife*. 8:e49110.
30. Guinier, A., and F. Fournet. 1955. Small Angle Scattering of X-rays. Wiley Interscience, New York.
31. Rambo, R. P., and J. A. Tainer. 2013. Accurate assessment of mass, models and resolution by small-angle scattering. *Nature*. 496:477–481.
32. Dyer, K. N., M. Hammel, …, G. L. Hura. 2014. High-throughput SAXS for the characterization of biomolecules in solution: a practical approach. *Methods Mol. Biol.* 1091:245–258.
33. Heller, W. T., V. S. Urban, …, D. A. Myles. 2014. The bio-SANS instrument at the high Flux isotope reactor of Oak Ridge National Laboratory. *J. Appl. Cryst.* 47:1238–1246.
34. Heller, W. T. 2010. Small-angle neutron scattering and contrast variation: a powerful combination for studying biological structures. *Acta Crystallogr. D Biol. Crystallogr.* 66:1213–1217.
35. Hopkins, J. B., R. E. Gillilan, and S. Skou. 2017. *BioXTAS RAW*: improvements to a free open-source program for small-angle X-ray scattering data reduction and analysis. *J. Appl. Cryst.* 50:1545–1553.
36. Franke, D., M. V. Petoukhov, …, D. I. Svergun. 2017. *ATSAS 2.8*: a comprehensive data analysis suite for small-angle scattering from macromolecular solutions. *J. Appl. Cryst.* 50:1212–1225.

37. Piiadov, V., E. Ares de Araújo, …, I. Polikarpov. 2019. SAXSMoW 2.0: online calculator of the molecular weight of proteins in dilute solution from experimental SAXS data measured on a relative scale. *Protein Sci.* 28:454–463.

38. Sali, A., and T. L. Blundell. 1993. Comparative protein modelling by satisfaction of spatial restraints. *J. Mol. Biol.* 234:779–815.

39. Pelikan, M., G. L. Hura, and M. Hammel. 2009. Structure and flexibility within proteins as identified through small angle X-ray scattering. *Gen. Physiol. Biophys.* 28:174–189.

40. Schneidman-Duhovny, D., M. Hammel, …, A. Sali. 2013. Accurate SAXS profile computation and its assessment by contrast variation experiments. *Biophys. J.* 105:962–974.

41. Schneidman-Duhovny, D., M. Hammel, and A. Sali. 2010. FoXS: a web server for rapid computation and fitting of SAXS profiles. *Nucleic Acids Res.* 38:W540–W544.

42. Schneidman-Duhovny, D., M. Hammel, …, A. Sali. 2016. FoXS, FoXSDock and MultiFoXS: single-state and multi-state structural modeling of proteins and their complexes based on SAXS profiles. *Nucleic Acids Res.* 44:W424–W429.

43. Schneidman-Duhovny, D., M. Hammel, and A. Sali. 2011. Macromolecular docking restrained by a small angle X-ray scattering profile. *J. Struct. Biol.* 173:461–471.

44. Konarev, P. V., V. V. Volkov, …, D. I. Svergun. 2003. PRIMUS: a Windows PC-based system for small-angle scattering data analysis. *J. Appl. Cryst.* 36:1277–1282.

45. Svergun, D. I., S. Richard, …, G. Zaccai. 1998. Protein hydration in solution: experimental observation by x-ray and neutron scattering. *Proc. Natl. Acad. Sci. USA.* 95:2267–2272.

46. Petoukhov, M. V., D. Franke, …, D. I. Svergun. 2012. New developments in the *ATSAS* program package for small-angle scattering data analysis. *J. Appl. Cryst.* 45:342–350.

47. Petoukhov, M. V., and D. I. Svergun. 2006. Joint use of small-angle X-ray and neutron scattering to study biological macromolecules in solution. *Eur. Biophys. J.* 35:567–576.

48. Svergun, D. I., M. V. Petoukhov, and M. H. Koch. 2001. Determination of domain structure of proteins from X-ray solution scattering. *Biophys. J.* 76:2879–2886.

49. Xiao, Y., Q. Ma, …, R. Hilgenfeld. 2012. Nonstructural proteins 7 and 8 of feline coronavirus form a 2:1 heterotrimer that exhibits primer-independent RNA polymerase activity. *J. Virol.* 86:4444–4454.

50. Franke, D., and D. I. Svergun. 2009. *DAMMIF*, a program for rapid *ab-initio* shape determination in small-angle scattering. *J. Appl. Cryst.* 42:342–346.

51. Baker, N. A., D. Sept, …, J. A. McCammon. 2001. Electrostatics of nanosystems: application to microtubules and the ribosome. *Proc. Natl. Acad. Sci. USA.* 98:10037–10041.

52. Dolinsky, T. J., J. E. Nielsen, …, N. A. Baker. 2004. PDB2PQR: an automated pipeline for the setup of Poisson-Boltzmann electrostatics calculations. *Nucleic Acids Res.* 32:W665–W667.

Biophysical Journal, Volume *120*

Supplemental information

Transient and stabilized complexes of Nsp7, Nsp8, and Nsp12 in SARS-CoV-2 replication

Mateusz Wilamowski, Michal Hammel, Wellington Leite, Qiu Zhang, Youngchang Kim, Kevin L. Weiss, Robert Jedrzejczak, Daniel J. Rosenberg, Yichong Fan, Jacek Wower, Jan C. Bierma, Altaf H. Sarker, Susan E. Tsutakawa, Sai Venkatesh Pingali, Hugh M. O'Neill, Andrzej Joachimiak, and Greg L. Hura

Supporting Material for "Transient and stabilized complexes of Nsp7, Nsp8 and Nsp12 in SARS-CoV-2 replication

Authors: Mateusz Wilamowski*, Michal Hammel*, Wellington Leite*, Qiu Zhang, Youngchang Kim, Kevin Weiss, Robert Jedrzejczak, Daniel J. Rosenberg, Yichong Fan, Jacek Wower, Jan Bierma, Altaf H. Sarker, Susan E. Tsutakawa, Sai Venkatesh Pingali, Hugh M. O'Neill#, Andrzej Joachimiak#, Greg L. Hura#

***contributed equally #corresponding authors**

Correspondence should be addressed to:

glhura@lbl.gov, oneillhm@ornl.gov, andrzejj@anl.gov

Supporting Material Materials and Methods

Macromolecular sequences, expression and purification prior to structural studies

Protein Sequences: NCBI reference sequences for Nsp7, Nsp8 and Nsp12 are YP_009725303.1, YP_009725304.1, and YP_009725307.1 respectively and are further available in the supplementary information.

Protein Sequences:

Nsp7:SKMSDVKCTSVVLLSVLQQLRVESSSKLWAQCVQLHNDILLAKDTTEAFEKMVSLLSVLLSMQGAVDINKLCEE
MLDNRATLQ

Nsp8:AIASEFSSLPSYAAFATAQEAYEQAVANGDSEVVLKKLKKSLNVAKSEFDRDAAMQRKLEKMADQAMTQMYK
QARSEDKRAKVTSAMQTMLFTMLRKLDNDALNNIINNARDGCVPLNIIPLTTAAKLMVVIPDYNTYKNTCDGTTFTYAS
ALWEIQQVVDADSKIVQLSEISMDNSPNLAWPLIVTALRANSAVKLQ

Nsp12:SADAQSFLNRVCGVSAARLTPCGTGTSTDVVYRAFDIYNDKVAGFAKFLKTNCCRFQEKDEDDNLIDSYFVVKR
HTFSNYQHEETIYNLLKDCPAVAKHDFFKFRIDGDMVPHISRQRLTKYTMADLVYALRHFDEGNCDTLKEILVTYNCCD
DDYFNKKDWYDFVENPDILRVYANLGERVRQALLKTVQFCDAMRNAGIVGVLTLDNQDLNGNWYDFGDFIQTTPGS
GVPVVDSYYSLLMPILTLTRALTAESHVDTDLTKPYIKWDLLKYDFTEERLKLFDRYFKYWDQTYHPNCVNCLDDRCILHC
ANFNVLFSTVFPPTSFGPLVRKIFVDGVPFVVSTGYHFRELGVVHNQDVNLHSSRLSFKELLVYAADPAMHAASGNLLL
DKRTTCFSVAALTNNVAFQTVKPGNFNKDFYDFAVSKGFFKEGSSVELKHFFFAQDGNAAISDYDYYRYNLPTMCDIRQ
LLFVVEVVDKYFDCYDGGCINANQVIVNNLDKSAGFPFNKWGKARLYYDSMSYEDQDALFAYTKRNVIPTITQMNLKY
AISAKNRARTVAGVSICSTMTNRQFHQKLLKSIAATRGATVVIGTSKFYGGWHNMLKTVYSDVENPHLMGWDYPKCD
RAMPNMLRIMASLVLARKHTTCCSLSHRFYRLANECAQVLSEMVMCGGSLYVKPGGTSSGDATTAYANSVFNICQAV
TANVNALLSTDGNKIADKYVRNLQHRLYECLYRNRDVDTDFVNEFYAYLRKHFSMMILSDDAVVCFNSTYASQGLVASI
KNFKSVLYYQNNVFMSEAKCWTETDLTKGPHEFCSQHTMLVKQGDDYVYLPYPDPSRILGAGCFVDDIVKTDGTLMIE
RFVSLAIDAYPLTKHPNQEYADVFHLYLQYIRKLHDELTGHMLDMYSVMLTNDNTSRYWEPEFYEAMYTPHTVLQ

Gene cloning and protein expression:

Genes coding of the Nsp7, Nsp8, and Nsp12 from SARS-CoV-2 (taxid: 2697049) were codon optimized for efficient expression in *E. coli*, and synthetized by Twist Bioscience, San Francisco CA. Cloning of the coding sequences were conducted as reported previously using ligation independent cloning (LIC) method (26). The sequences of Nsp12, and Nsp7 were cloned into the pMCSG53 vector, that possesses an N-terminal His-Tag followed by a Tobacco Etch Mosaic Virus (TEV) protease cleavage site. The coding sequence of

Nsp8 was inserted into pRSF vector to obtain untagged protein, and to pMCSG53 for purification of Nsp8 with His-Tag. The protein expression plasmids were transformed into the E. coli BL21(DE3)-Gold strain (Stratagene, San Diego CA). For purification of Nsp7/8/12, and Nsp8/12 complexes we co-expressed Nsp12 (pMCSG53), and Nsp8 (pRSF) in single cell culture. For purification of Nsp7/8 complex we co-expressed Nsp8 (pRSF), and Nsp7 (pMCSG53). We also expressed Nsp7, Nsp8, and Nsp12 using pMCSG53 vectors containing N-terminal His-Tag. The large-scale expression of the the SARS-CoV-2 proteins were done in LB Lennox medium supplemented with ampicillin 150 µg/ml. The cells were grown at 37°C (190 rpm) until OD_{600nm} was approximately equal to 1. After that, the incubator temperature was changed to 4°C to cool down the bacterial cell suspension. When the culture reached 18°C the medium were supplemented with the following compounds at concentrations 0.2 mM IPTG, 0.1% glucose, 40mM K_2HPO_4. Next, the induced bacteria culture was grown for 20 hours at 16°C with 190 rpm shaking. Subsequently cells were harvested by centrifugation at 7k RCF, and cell pellets were resuspended in lysis buffer comprising 50 mM HEPES pH 8.0, 500 mM NaCl, 5% v/v glycerol, 20 mM imidazole, and 10 mM β-mercaptoethanol.

Deuterium labeled Nsp7, for contrast variation SANS experiments, was expressed using a protocol established by Marley et al. (27). Briefly, LB cultures were grown and monitored until OD_{600nm} reached ~0.7. At that point, the cells were pelleted by centrifugation at 4000×g for 10 minutes in pre-sterilized bottles. Each pellet was then washed in deuterated minimal medium and re-pelleted by centrifugation at 4000×g for 10 minutes. The washed pellets were then resuspended in fresh deuterated minimal medium and transferred to dry, sterile 2.8L Fernbach flasks (250 mL per flask). The cells were then cultivated at 18 °C for 1 hour, induced with 0.2 mM IPTG for ~20 hours, and harvested by centrifugation at 4000×g for 30 minutes. After decanting the supernatant, the protiated and deuterated pellets were stored at -80°C until needed for subsequent protein purification.

Protein purification:

Harvested *E. coli* cell pellets were sonicated on ice at 120W for 5 minutes (4 sec pulses of sonication followed by 20 sec breaks). Cell debris were removed through centrifugation at 30k RCF, 4°C for 1 hour. Supernatants were mixed with 5 ml of Ni^{2+} Sepharose (GE Healthcare Life Sciences, Marlborough MA) previously equilibrated with lysis buffer. Solutions were transferred on chromatography Flex-Column (420400-2510) attached to Vac-Man vacuum system (Promega, Madison WI). After loading of the cell lysate the nickel beads were washed three times with 60 ml of lysis buffer. As next, proteins were eluted using 20 ml of lysis buffer enriched with 500 mM imidazole pH 8.0. Protein concertation's were measured using NanoDrop (Wilmington, DE) spectrophotometer. Subsequently after determination of sample concentration the TEV protease were added in molar ratio 1:30 (TEV : protein). These solutions were incubated for 16 hours at 4°C.

To obtain the Nsp7/8/12 complex we purified Nsp8/12 complex from co-expression system, and as next we added separately purified Nsp7, His-Tags were removed from both Nsp7, and Nsp12 using TEV protease. For purification of Nsp8/12 complex we used co-expression system with a His-Tag on Nsp12. For purification of Nsp7/8 we used a co-expression system with His-Tag on Nsp7. We also purified the Nsp7, Nsp8, and Nsp12 separately using the TEV to remove N-terminal His-Tag attached to the proteins. Subsequently, all purified proteins or obtained complexes were concentrated using 30 kDa cut-off

centrifugal protein concentrators (Merck-Millipore, Burlington, MA). The complex of Nsp7/8/12 was concentrated to 20 mg/ml, the Nsp7/8 was concentrated to 50 mg/ml, and Nsp8, Nsp7, Nsp8/12, Nsp12 were concentrated to approximately 30 mg/ml. Depending on the sample volume, SEC was carried out using a Superdex 200 column in lysis buffer containing 1 mM TCEP instead of β-mercaptoethanol. After SEC gel electrophoresis was done to select the pure fractions that were frozen for further analysis. For purification of Nsp7, Nsp8, Nsp12 the selected fractions after SEC were pooled together and subsequently loaded on Ni^{2+} Sepharose to remove TEV, and contaminating material that binds to the nickel column. Flow-through containing pure protein were concentrated using centrifugal concentrators, and buffer was exchanged with 20 mM HEPES pH 7.5, 150 mM NaCl, 1 mM TCEP, pH 7.5. Protein purity was analyzed and compared with Precision Plus Protein™ Dual Color Standard (BioRad, Hercules, CA) using SDS-PAGE in Tris-glycine buffer and molecular markers. For screening crystallization conditions, we used freshly purified proteins. Proteins were flash cooled at liquid nitrogen for subsequent SAXS and SANS data collection.

Co-purification of deuterated Nsp7 and protiated Nsp8 complex:

The overexpressed deuterated Nsp7 (dNSP7) and protiated Nsp8 *E. coli* cell pastes were resuspended in 50 mL lysis buffer containing 50 mM HEPES, 500 mM NaCl, 20 mM imidazole, 5% glycerol, 1 mM TCEP and EDTA-free protease inhibitor tablet Complete (05056489001, Roche, USA), respectively. The resuspended cells were mixed together and lysed by sonication on ice for 10 min with 50% amplitude using a 4 sec on/10 sec off cycle. The clarified lysate was loaded onto 5 mL HisTrap^{TM} HP column (17524802, Cytiva, Marlborough MA) after centrifugation at 18,000 rpm for 30 min. The column was washed by 10 column volumes (CV) of buffer A (50 mM HEPES, 500 mM NaCl, 10 mM imidazole, 5% glycerol, and 1mM TCEP), 10 CV of 6% buffer B (50 mM HEPES, 500 mM NaCl, 500 mM imidazole, 5% glycerol, and 1 mM TCEP) before elution. The protein was eluted with a 10 CV, 6 to 100% linear gradient. The fractions containing dNsp7 and Nsp8 were confirmed by SDS-PAGE and pooled. The pooled fractions were dialyzed against buffer containing 50 mM HEPES, 300 mM NaCl, 5% glycerol, 1 mM TCEP with TEV protease (1:50 ratio) at 4°C for 16 hours. The cleaved protein was loaded to a 5-mL HisTrap^{TM} HP column to remove TEV protease and other contaminants. Flow-through was collected and concentrated using a centrifugal concentrator with a 30kDa MW cutoff and then loaded to a HiLoad 16/60 Superdex 200 column equilibrated in 50 mM HEPES, 150 mM NaCl, 1 mM TCEP for final. After pooling and concentrating the peak fractions, the purity and concentration of the purified protein were determined by SDS-PAGE and UV-Vis spectrophotometry. Using dialysis, the relative ratio of D_2O/H_2O in the protein solution was adjusted to 90% D_2O. In 90% D2O solvent, the scattering contribution of dNSP7 was negligible because the scattering length density of dNSP7 was matched to that of the solvent.

RNA extension essay

The RNA used in the extension assay folds into a hairpin with a GAAA tetraloop and a single-stranded tail, 5’-GGCUU-3’, at its 5’ terminus (Fig. S1). Its sequence is composed of 31 nucleotides: 5’-pppGGCUUAGGAGAUGAUGAAAGUCAUUCUCCU-OH-3’. We synthesized the 31-mer by *in vitro* transcription as described previously (28). Oligonucleotides used for the construction of the DNA template are:5’-AATTCCTGCAG**TAATACGACTCACTATA**GGCTTAGGAGAATGATGAAAGTC-3’ and 5’-mAmGGAGAATGACTTTCATCATTCT-3’. mA and mG represent the 2’-O-methyl nucleotides used to reduce the proportion of runover transcripts. The underlined portions represent the overlapped segment and bold type indicates the T7 RNA polymerase promoter. To synthesize the internally [^{32}P]-labeled 31-mer

transcripts, 10 ug/ml duplex DNA in a solution containing 200 mM HEPES/KOH (pH7.5), 30 mM $MgCl_2$, 2 mM spermidine, 40 mM DTT, 3 mM each ATP, CTP, GTP and UTP, 0.33 µM [alpha-^{32}P]ATP was incubated with 100 µg/mL T7 RNA polymerase, 5 U/ml inorganic phosphatase (New England BioLabs, Boston MA), SUPERase·In RNase Inhibitor (Invitrogen, Walton MA) in a 50 µL reaction for 5 hours at 37 °C. DNA template was removed by addition of 50 U/ml RQ1 RNase-free DNase (Promega, Madison WI) and incubation for 15 min at at 37 °C, followed by phenol-chloroform extraction. The 31-mer RNA was recovered from the aqueous phase by ethanol precipitation and then purified by electrophoresis on a 15% denaturing polyacrylamide gel. To remove homodimers and produce a homogeneous solution of hairpins, the gel-purified RNA transcripts dissolved in water were heated to 75 °C and gradually cooled to room temperature. The RNA extension reactions contained 10 µM 31-mer RNA hairpin, 5 µM Nsp12, 10 µM Nsp8, 5 µM Nsp7 in 20 mM HEPES/KOH (pH 7.5), 75 mM NaCl, 2 mM $MgCl_2$ and 0.5 mM TCEP. Reactions were incubated for 30 min at 30 °C and the RNA extension was initiated by addition of 1 mM each ATP, CTP, GTP and UTP. Reactions were stopped by the addition of 2.5 mM EDTA (pH 8.0) followed by phenol-chloroform extraction. RNA products of extension reactions were recovered from the aqueous phase by ethanol precipitation, analyzed by electrophoresis on a 15% denaturing polyacrylamide gel and visualized by autoradiography.

RNA and DNA constucts:

long ssRNA: 36 bases (5'-UUU UCA UGC UAC GCG UAG CAU GCU ACG CGU AGC AUG-3')

short ssRNA: 28 bases (5'-CAU GCU ACG CGU AGC AUG CUA CGC GUA G-3')

The RNA and DNA constructs were highly purified synthetic polynucleotides ordered from IDT. The dsRNA consisted of duplexed long ssRNA (36 bases) paired to short ssRNA (28 bases). RNA oligos were dissolved and annealed in water. Both RNAs were validated by the SEC-MALS-SAXS experiment on a Shodex KW403 (Shodex, Tokyo Japan) column using 50 mM HEPES (pH 7.5), 150 mM NaCl, 1 mM TCEP running buffer. Both MALS and SAXS measurements agree with models of monodispersed dsRNA and ssRNA (Fig. S3). The atomistic models of both RNA's give an excellent match to the SAXS data (Fig. S3C).

Crystallization, data collection, and structural analysis of Nsp7/8 complex

For crystallizations trials we used 400 nl protein solution mixed with 400 nl of buffer reservoir solution. The sitting drops were equilibrated against 130 µl of reservoir solution. Screening of crystallization condition of Nsp7/Nsp8 complex were performed at 289K with MCSG1, MCSG2, MCSG3, MCSG4, INDEX, Natrix HT crystallizations screens (Anatrace, Hampton Research, Maumee, OH; Hampton Research, Aliso Viejo, CA). NSP7/8A (PDB: 6WIQ) crystals were obtain from 20 mg/ml protein concentration in crystallization condition 0.1 M Tris pH 8.5, 1.5 M ammonium phosphate dibasic. Crystals suitable for high-resolution diffraction data collection appeared after three weeks. For Nsp7/8B (PDB entry 6WQD) the crystal of the Nsp7/8 was obtained using 40 mg/ml of the complex in the buffer: 0.2 M magnesium chloride, 0.1 M Tris, pH 8.5, 20% w/v PEG8000. The Nsp7/8C (PDB entry 6XIP) crystal grow from 40 mg/ml in the buffer: 0.2 M magnesium chloride hexahydrate, 0.1 M BIS-TRIS pH 5.5, 25% w/v polyethylene glycol 3350. The Nsp7/8B and C (PDB entries 6WQD and 6XIP) crystals were both obtained in a presence of V8 protease. Before setting the crystallization plates to purified Nsp7/8 we added endo-proteinase Glu-C (V8 protease) from Staphylococcus aureus (Sigma Aldrich, St. Louis MO). We used 300 times excess of Nsp7/8

over V8 protease. After 16 hours of incubation of the Nsp7/8 complex with V8 protease we set up the crystallization plates.

Prior to data collection at 100 K, all cryoprotected crystals of Nsp7-Nsp8 complex were flash-cooled in liquid nitrogen. The x-ray diffraction experiments were carried out at the Structural Biology Center 19-ID beamline at the Advanced Photon Source, Argonne National Laboratory. The diffraction images were recorded from the crystals of three different forms Nsp7/8A, Nsp7/8B, and Nsp7/8C on the PILATUS3X 6M detector using 0.3-0.5° rotation on ω and 0.5 sec exposure for 210°, 375°, and 260°, and to resolution of 2.9 Å, 1.95 Å, and 1.50 Å, respectively. For Nsp7/8B crystals, the diffractions were collected from three different spots from a crystal. The data sets were processed and scaled with the HKL3000 suite (29). Intensities were converted to structure factor amplitudes in the Ctruncate program (30, 31) from the CCP4 package (32). The structures were determined using molrep (33) implemented in the HKL3000 software package using the SARS-CoV Nsp7/8 complex structure (PDB id 5F22) as a search model. Initial models were refined as rigid bodies and then refined all atoms by 12 cycles of REFMAC (32, 34) before they were iteratively refined using COOT and PHENIX (35). Throughout the refinement, the same 5% of reflections were kept out from the refinement in both REFMAC and PHENIX refinement. The final structures converged to R_{work} = 0.218 and R_{free} = 0.252 for Nsp7/8A, R_{work} = 0.187 and R_{free} = 0.229 for Nsp7/8B, R_{work} = 0.161 and R_{free} = 0.199 for Nsp7/8C with regards to each data quality. The stereochemistry of the structures were checked with PROCHECK (36) and the Ramachandran plot and validated with the PDB validation server. The data collection and processing statistics are given in Table S1. The atomic coordinates and structure factors have been deposited in the Protein Data Bank under accession code 6WIQ, 6WQD and 6XIP for Nsp7/8A, Nsp7/8B, and Nsp7/8C, respectively.

Size-exclusion chromatography coupled with multi-angle light scattering and small-angle X-ray scattering (SEC-MALS-SAXS)

For SEC-MALS-SAXS experiments, 60 μL containing either 10 mg/ml Nsp7; 8 mg/ml Nsp8; 8 mg/ml Nsp7/8; 6 mg/ml Nsp8/12; 6 mg/ml and Nsp7/8/12; in 50 mM HEPES pH 7.5, 150 mM NaCl, 1mM TCEP were utilized. Additionally, we also performed SEC-MALS-SAXS for the mixture of dsRNA or ssRNA with the Nsp7, Nsp8, Nsp7/8, Nsp12/8, and Nsp7/8/12 in 1:1 molar ratio.

SEC-MALS-SAXS data were collected at the Advanced Light Source (ALS) beamline SIBYLS (beamline 12.3.1) in Berkeley, California (37, 38). The X-ray wavelength was set at λ=1.127 Å, and the sample-to-detector distance was 2070 mm, resulting in scattering vectors, q, ranging from 0.01 $Å^{-1}$ to 0.35 $Å^{-1}$. The scattering vector is defined as $q = 4\pi \sin\theta/\lambda$, where 2θ is the scattering angle. All experiments were performed at 20°C and data was processed as described (39). Briefly, a SAXS flow cell was coupled in-line with an Agilent 1290 Infinity HPLC system using a Shodex KW803 column equilibrated at a 0.5 mL/min flow rate with the running buffer as indicated above. 55 µL of each sample was run through the SEC column and 2s X-ray exposures were collected continuously during a 20-minute elution. The SAXS frames recorded prior to the protein elution peak were used to subtract all other frames. The subtracted frames were investigated by the radius of gyration (Rg) derived by the Guinier approximation $I(q) = I(0) \exp(-q^2R_g^2/3)$ with the limits q R_g<1.5 (40). The elution peak was mapped by comparing the integral ratios to background and R_g relative to the recorded frame using the program SCÅTTER. Non-uniform Rg values across an elution peak represent a heterogeneous assembly. Final merged SAXS profiles, derived by

integrating multiple frames at the peak of the elution peak or further indicated, were used for analysis, including the Guinier plot, which determined aggregation-free state. The program SCÅTTER was used to compute the pair distribution function (P(r)). The distance r where P(r) approaches zero intensity identifies the maximal dimension of the macromolecule (Dmax) (Table S2). P(r) functions were normalized based on the molecular weight of the assemblies as determined by SCÅTTER using the volume of correlation Vc (41) (Table S2). The SAXS flow-cell was additionally connected inline to a 1290 series UV-vis diode array detector (DAD) measuring at 280 and 260 nm(Aglient), 18-angle DAWN HELEOS II multi-angle light scattering (MALS) and quasi-elastic light scattering (QELS), and Optilab rEX refractometer (Wyatt Technology, Santa Barbra CA). System normalization and calibration were performed with a BSA monomer using a 45 µL sample at 10 mg/mL in the same running buffer and a dn/dc value of 0.175. The light scattering experiments were used to perform analytical scale chromatographic separations for mass and hydrodynamic radius (Rh) determination. UV, MALS, and differential refractive index data were analyzed using Wyatt Astra 7 software to monitor sample homogeneity across the elution peak complementary to the above-mentioned SEC-SAXS signal validation. The SEC-MALS signal was used as an indicator of homogeneous vs. heterogeneous samples.

High-throughput SAXS and SANS

SAXS data were collected in "batch" high throughput mode (HT-SAXS) at the ALS beamline 12.3.1 (SIBYLS) at LBNL Berkeley (42) on Nsp8 at 10, 5 and 2.5 mg/mL because of the concentration dependence we observed in SEC-SAXS and differences from SANS data. Experiments were performed 20^0C as described elsewhere (37). Briefly, the sample was exposed for 10 s with the detector framing at 0.3 s to maximize signal while ensuring only non-radiation damaged signal is included in the data for analysis. Once radiation damage data was removed and a SAXS profile was integrated, processing occurred using the SCÅTTER package as described for the SEC-SAXS profiles.

The protein concentrations for SANS measurements were 1.5 mg/mL Nsp7/8; 4 mg/mL Nsp8; 4 mg/mL deuterated Nsp7/Nsp8 complex, and mixture of Nsp7/8/dsRNA in 1:1 molar ratio, at 3.5 mg/mL Nsp7/8 complex in the same buffer as was performed SAXS experiments. For the contrast matching SANS experiments of deuterated dNsp7 with protiated Nsp8 (dNsp7/Nsp8 complex), 90% D_2O buffer was used to selectively highlight the scattering from Nsp8. Studies of the Nsp7/8/dsRNA mixture were performed in 65% D_2O buffer to selectively highlight the scattering from Nsp7/8 complex.

SANS measurements were collected at the Bio-SANS and EQ-SANS instruments located at the High Flux Isotope Reactor and Spallation Neutron Source (SNS), respectively, at Oak Ridge National Laboratory (43, 44). At the Bio-SANS, a single configuration of the dual detector system was used with the main detector at 7m (SSD) and the wing detector at 3.2°. Using this configuration, the *q* range spanning $0.007 < q$ ($Å^{-1}$) < 1 was obtained using 6 Å wavelength neutrons with an ($\Delta\lambda/\lambda$) of 15%. At EQ-SANS, two instrument configurations were used - 2.5Å and 2.5m (SSD) for high-*q* and 10Å and 4m (SSD) for low-*q* to provide an *q*-range of 0.006 to 1.2 $Å^{-1}$. The data were corrected for instrument background, detector sensitivity, and instrument geometry using facility data reduction software, drt-SANS. All SANS measurements were performed in 1 mm path length cylindrical quartz cuvettes (Hellma, Müllheim, Germany) at 10°C. Initial SANS data analysis, including Guinier fits and pair-distribution calculations, were performed using the BioXTAS RAW program and ATSAS suite(45),(46). The pair distance distribution function (P(r)) was

calculated using the indirect Fourier transform method implemented in the program GNOM (47). Scattering data over the range 0.007 < q (Å^{-1}) < 8/R_g were used for P(r) analysis and subsequent modeling. The SAXS-derived molecular weight was determined using the volume of Porod method as implemented in RAW (48).

Solution Structure Modeling.

Nsp7: To fit experimental SEC-SAXS curves of Nsp7 dimer, we initially modeled an Nsp7 dimer by adding missing C and N-terminal regions into our Nsp7 structure taken from the Nsp7/8 crystal structure (PDBID: 6WIQ) by MODELLER (49). To test whether the discrepancy between SAXS and Nsp7 dimer structure was due to the flexibility of exposed C-terminal helix (68-86), we perform rigid body modeling using BILBOMD (19). In this step, the disulfide bond (Cys8-Cys8) was preserved. The experimental SAXS was then compared to theoretical scattering curves generated from atomistic models using the FOXS (17, 18) and followed by multistate model selection by MultiFOXS (21). The bad SAXS fit leads us to search for the alternative Nsp7 dimer by bypassing disulfide bond restraints. We performed SAXS based docking of two Nsp7 monomers using FOXSDock approach(50). The best score model was superimposed on the average SAXS envelope calculated by GASBOR with a P2 symmetry operator (51) (Fig. 3C).

Nsp8 monomer: To find an Nsp8 conformation that fits the SEC-SAXS Data, we built an Nsp8 monomer by adding missing C and N-terminal regions into the Nsp8 monomer, taken from Nsp7/8/12/RNA structure (PDBID: 6YYT)(16). We employed a conformational sampling of the N-terminal helix-bundle region using BILBOMD (19). The flexible tethers in between the head and two distinct helix regions (1-82 and 86-100) were identified by structural comparison of the two Nsp8 conformers from the Nsp7/8/12/RNA complex (16). The experimental SAXS profiles were then compared to theoretical scattering curves generated from atomistic models using the FOXS (17, 18) followed by multistate model selection by MultiFoXS (21). Despite providing a nearly exhaustive search of extended and compact conformations, an excellent fit to the SAXS data was obtained for a single closed state (Fig. 3D).

Nsp8 dimer and tetramer: To find a multistate model that fits a higher oligomerization state collected in HT-SAXS mode or at the peak of SEC elution, we built the Nsp8 dimer and tetramer. Missing regions were added to the Nsp8 dimer taken from the crystal structure of the Nsp7/8 complex (PDBID: 3UB0) (22). By keeping the dimerization interface, we used BILBOMD (19) to build an Nsp8 dimer with open and close C-terminal head regions. Additionally, we built the Nsp8 tetramer by adding missing regions into the Nsp8 tetramer taken from the crystal structure of Nsp7/8 (PDBID: 2AHM). The experimental SAXS profiles were then compared to theoretical SAXS curves generated from the pool of the monomers, dimers, and tetramer using the FOXS (17, 18) followed by multistate model selection by MultiFOXS (21) (Fig. S4).

Nsp12: To fit experimental SEC-SAXS curves of Nsp12 monomer, we add missing regions into Nsp12 structure, taken from Nsp7/8/12/RNA cryo-EM structure (PDBID: 6YYT), by using MODELLER (49). The experimental SAXS profiles were then compared to theoretical scattering curves generated from atomistic models using the FOXS (17, 18) (Fig. 3E). The experimental SANS profile of Nsp8 was compared to theoretical SANS of an Nsp8 tetramer built using the SAXS model of Nsp8 dimer (PDBID: G1TQHV) using SAXS/REFMX to perform the quaternary structure modeling (52, 53).

Nsp7/8: To find a multistate model that fit SEC-SAXS data for Nsp7/8, we initially build a complete model of Nsp7/8 heterotetramer by combining our crystal structure (PDBID: 6WIQ) and Nsp8 structure taken from Nsp7/8/12/RNA structure (PDBID: 6YYT)(16). The experimental SAXS profile was then compared to theoretical SAXS curves generated from the pool of the Nsp8 monomers, Nsp8 dimers, Nsp7 dimers, Nsp7/8 heterotetramer, and Nsp7/8 heterodimer using the FOXS (17, 18) followed by multistate model selection by MultiFOXS (21) (Fig. 4E). The experimental SANS profile of Nsp7/8 complex was compared to theoretical SANS profile using a mixture of SAXS models of Nsp7/8 heterotetramer (PDBID: DJTRUW) and Nsp8 monomer (PDBID: IDDWOG). OLIGOMER was used to determine the volume fraction of component (54). The experimental SANS profile of dNsp7/Nsp8 complex was compared to theoretical SANS profile using a mixture of SAXS models of Nsp8 dimers (PDBID: DJTRUW) and Nsp8 monomers (PDBID: IDDWOG). SAXS/REFMX was used to perform the quaternary structure modeling to account for polydispersity of the system.

Nsp8/12 and Nsp7/8/12: To find a model that fits SEC-SAXS data of Nsp8/12 and Nsp7/8/12, we initially built a complete model of Nsp7/8/12 using the cryo-EM Nsp7/8/12/RNA structure (PDBID: 6YYT) (16). The experimental SAXS curves were then compared to theoretical SAXS curves generated from the pool of the models: (Nsp7 dimer, Nsp8 monomers, Nsp8 dimers, Nsp12 monomer, Nsp8/12 with 1:1 and 2:1 ratios; and Nsp7/8/12 with 1:1:1, 1:2:1, 0:1:1, 0:2:1 ratio) using the FOXS (17, 18) followed by multistate model selection by MultiFOXS (21). Despite providing a large pool of the models to select a multistate model (21) an excellent fit for the SAXS data required only Nsp8/12 1:1 conformer to fit both data sets (Fig. 5D).

Nsp8/dsRNA: To find a model that fits SEC-SAXS data of Nsp8/dsRNA complex, we built a complete model of Nsp8dsRNA using the cryo-EM Nsp7/8/12/RNA structure (PDBID: 6YYT) (16). The flexible tethers in between the head (101-199) and helix bundle regions (1-82 and 86-100) were used to perform conformational sampling of the head region using BILBOMD (19). The experimental SAXS profiles were then compared to theoretical scattering curves generated from atomistic models using the FOXS (17, 18) followed by multistate model selection by MultiFoXS (21). Despite providing a nearly exhaustive search of extended and compact conformations, an excellent fit to the SAXS data was obtained for a single closed state (Fig. 4C). The Nsp8/dsRNA conformers pool, together with the above characterized Nsp7 dimer, were used to select a multistate model by MultiFoXS (21) for Nsp7/8+ dsRNA SEC-SAXS data (Fig. 4F).

Nsp7/8/12/dsRNA and Nsp7/8/12/ssRNA: To fit SEC-SAXS data of Nsp7/8/12/dsRNA complex, we built a complete model of Nsp7/8/12/dsRNA using cryo-EM structure (PDBID: 6YYT) (16). Despite providing a large pool of the models to select a multistate model (21) an excellent fit for the SAXS data required only a complete complex of Nsp7/8/12/dsRNA with 1:1:2:1:1 ratio (Fig. 5D). Nsp7/8/12/ssRNA fit SEC-SAXS data of Nsp7/8/12/ssRNA complex with 1:1:2:1:1 ratio model using longer ssRNA (Fig. 5D). The experimental SANS profile of Nsp7/8/dsRNA mixture was compared to theoretical SANS profile using the SAXS model of Nsp8 monomer (PDBID: 7MRNJA) CRYSON (55).

Table S1. Data Collection and Refinement Statistics

	Nsp7-8A	Nsp7-8B	Nsp7-8C
**Crystallization Conditions**			
**Data Collection**			
Space group	$C222_1$	$P2_1$	$P2_1$
Unit cell parameters (Å;º)	*a* = 52.15, *b* = 70.78, *c* = 115.46; α = β = γ = 90.0	*a* = 47.26, *b* = 55.10, *c* = 84.40; α = γ = 90.0, β = 90.4	*a=35.62, b=119.90, c=43.38;* α = γ = 90.0, β = 92.2
Resolution range (Å) [a]	50.00 – 2.85 (2.90 - 2.85)	50.00 - 1.95 (1.98 - 1.95)	50.00 – 1.50 (1.53-1.50)
No. of reflections	5,144 (235) [a]	30,923 (1,220)	56,701 (2,392)
R_{merge} [b]	0.13 (1.11)	0.18 (1.15)	0.083 (0.84)
Completeness (%)	97.9 (88.7)	97.3 (77.9)	97.7 (83.9)
$CC_{1/2}$ [c]	0.853 (0.468)	0.962 (0.456)	0.999 (0.826)
$I/\sigma(I)$	18.2 (1.01)	22.5 (1.1)	30.2 (1.85)
Multiplicity	6.7 (4.4)	8.7 (2.2)	4.7 (3.0)
Wilson *B* factor	105.9	38.6	22.8
**Structure Determination**			
MR initial model (PDB ID)	5F22	6WIQ	6WIQ
**Refinement**			
Resolution range (Å)	35.39 - 2.85 (3.59 - 2.85)	41.12 - 2.00 (2.01 - 1.95)	40.76 - 1.50 (1.53 -1.50)
Completeness (%)	97.2 (96.0)	96.2 (73.0)	97.6 (82.0)
No. of reflections	5,108 (2,462)	30,700 (2,095)	56,635 (2,468)
R_{work}/R_{free} [d] (%)	20.8/25.2 (31.2/32.3)	18.7/22.9 (32.6/41.4)	16.1/19.9 (21.6/25.5)
Protein chains/atoms	2/1,507	4/3,077	4/2,988
Ligand/Solvent atoms	-	12/84	20/235
Mean temperature factor (Å^2)	125.6	53.1	35.5
**Coordinate Deviations**			
R.m.s.d. bonds (Å)	0.001	0.008	0.011
R.m.s.d. angles (º)	0.357	0.829	1.158
**Ramachandran plot** [e]			
Favored (%)	96.86	98.04	99.19
Allowed (%)	2.62	1.68	0.81
Outside allowed (%)	0.52	0.28	0.0
**PDB Accession Code**	6WIQ	6WQD	6XIP

[a] Values in parentheses correspond to the highest resolution shell.
[b] Rmerge = ΣhΣj|Ihj–<Ih>|/ΣhΣjIhj, where Ihj is the intensity of observation j of reflection h.
[c] As defined by Karplus and Diederichs (56). [d]R = Σh|Fo|–|Fc|/Σh|Fo| for all reflections, where Fo and Fc are observed and calculated structure factors, respectively. R_{free} is calculated analogously for the test reflections, randomly selected and excluded from the refinement. [e]As defined by Molprobity (57)

Table S2. Structural parameters from SAXS, SANS and MALS data

SAXS sample SimpleScattering ID#	D_{max}[a] (Å)	R_g (Å) from Guinier plot	Rg (Å) from P(r)	MW Seq. Monomer (kDA)[b]	MW SAXS[b] or SANS (kDa)	MW MALS (kDa)	Model fit[d] χ^2	data source
Nsp7 XSJUFZRI	~ 65	18.6± 0.6	18.8	9	19	17	1.5	SEC-SAXS peak
Nsp8 XSIDDWOG	~ 90	24.6± 0.7	25.8	22	29	28	1.4	SEC-SAXS tail
Nsp8 XSG1TQHV	~ 110	26.8± 0.1	27.8	22	33	35	1.9	SEC-SAXS peak
Nsp8	~ 110	29.9± 0.9	30.6	22	38	ND	ND	SANS
Nsp12 XSQIWPPC	~ 110	31.1± 0.5	41.9	107	88	93	1.3	SEC-SAXS
Nsp7/8 XSDJTRUW	~ 100	28.8± 0.1	30.3	31	42	45	2.2	SEC-SAXS peak
Nsp7/8	~ 110	30.0± 0.1	ND	31	56	ND	ND	SANS
Nsp8+dsRNA XS7MRNJA	~ 120	32.2± 0.6	29.8	45	46	45	1.7	SEC-SAXS peak
Nsp8+ssRNA XSXMU3AK	~105	30.3± 0.2	31.4	33	35	35	ND	SEC-SAXS peak
Nsp7/8 +dsRNA XSTKZWCR	~ 105	28.9± 0.2	31	54	32	30	2.1	SEC-SAXS peak
Nsp8/12 XSPPENAB	~ 115	34.8± 0.5	35.2	129	118	131	1.4	SEC-SAXS peak
Nsp7/8/12 XSX1TKUI	~ 115	34.7± 0.7	35.2	138	114	132	2.2	SEC-SAXS peak
Nsp7/8/12 +dsRNA XSZWMCYC	~ 140	41.8± 1.7	43.0	159	170	195	1.8	SEC-SAXS peak
Nsp7/8/12 +ssRNA XSE38FZM	~ 145	41.1± 2.2	43.0	126	170	190	1.7	SEC-SAXS peak

a) Maximal dimension (Dmax) defined from pair distribution function (P(r)) calculated by SCATTER
b) Theoretical MW calculated for monomer or assembly within the equal molar ratio
c) MW determined by volume of correlation Vc (41)
d) Goodness of fit (χ^2) for the atomistic models is determined FOXS(17, 18) for single model or MultiFOXS(50) for multistate model.

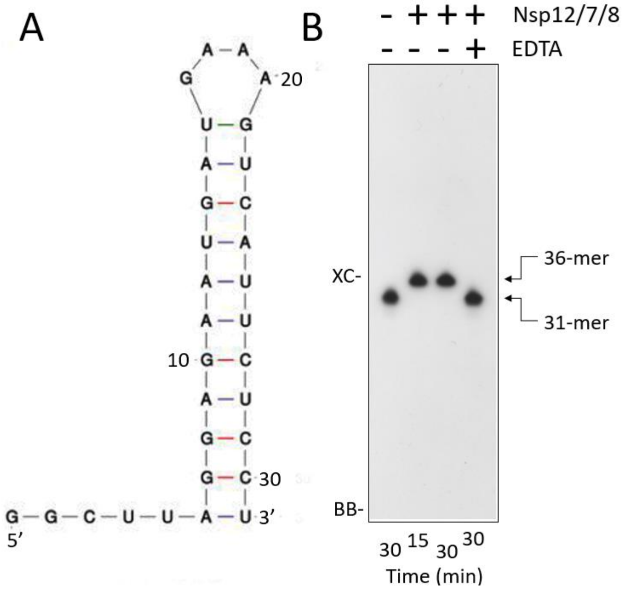

FIGURE S1. SARS-CoV-19 RTC activity**.** (A) Minimal substrate for RTC: a hairpin composed of 31 nucleotides. (B) Analysis of the minimal substrate extension by the Nsp12/7/8 complex on a 15% denaturing polyacrylamide gel. The hairpin was [^{32}P]-labeled and its extension was monitored for 30 min. 2.5 mM EDTA inhibits the extension reaction. BB and XC denote positions bromophenol blue

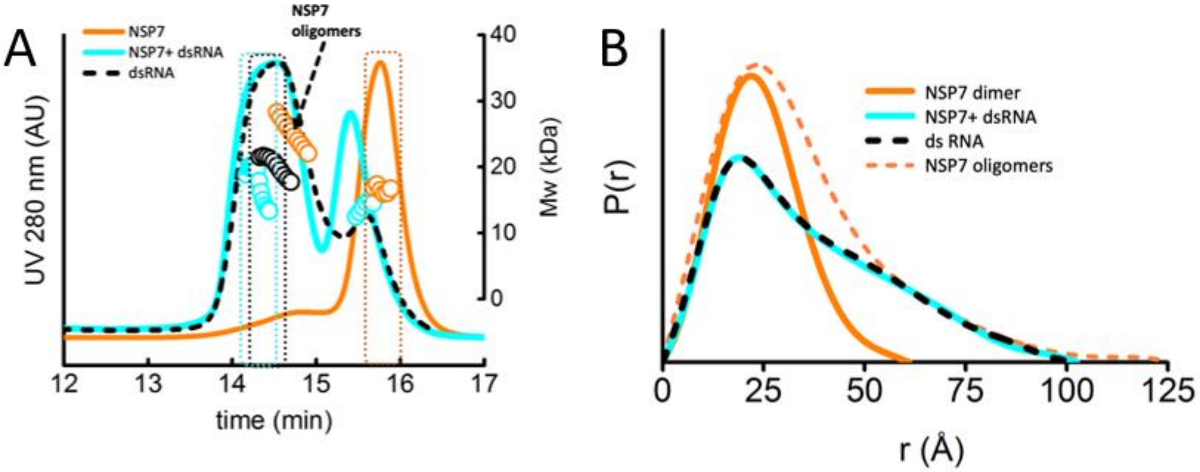

FIGURE S2 Nsp7 does not interact with dsRNA. (*A*) SEC-MALS chromatograms for Nsp7, Nsp7+dsRNA, and dsRNA are colored as indicated. Solid lines represent the UV at 280nm in arbitrary units, while symbols represent molecular mass versus elution time. (*B*) Normalized P(r) function calculated for SAXS profiles of Nsp7 dimer, Nsp7+dsRNA, and dsRNA collected at the SEC peak and Nsp7 oligomers collected at the SEC-leading peak. The similarity of P(r) functions between Nsp7+dsRNA and dsRNA further confirms the absence of Nsp7+dsRNA complexation.

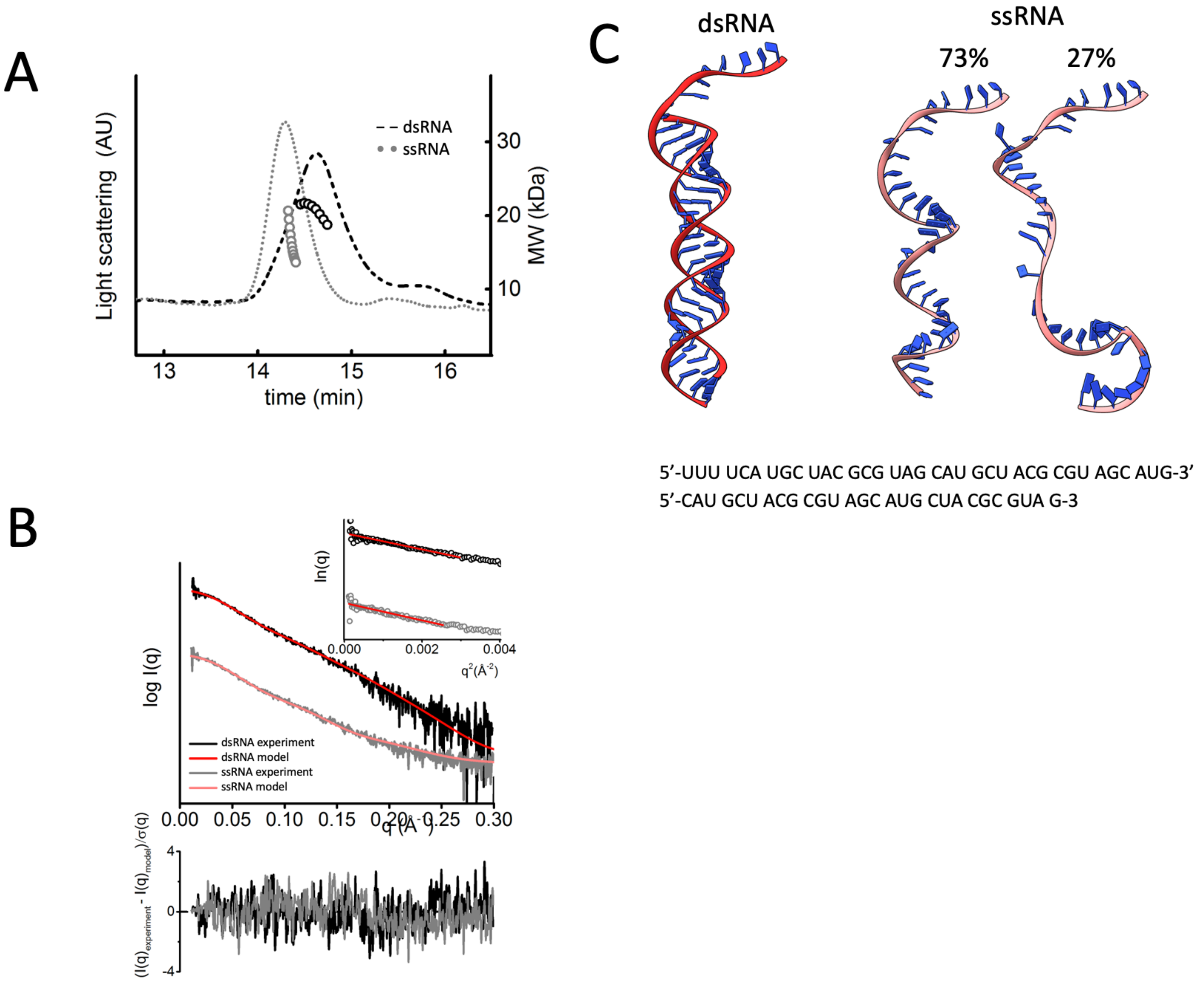

FIGURE S3. Monodisperse dsRNA and ssRNA. (*A*) SEC-MALS chromatograms for dsRNA and dsRNA are colored as indicated. Solid lines represent the UV at 280nm in arbitrary units, while symbols represent molecular mass versus elution time. (*B*) Experimental SAXS profiles for dsRNA (black) and ssRNA (gray) collected at the SEC peak are shown together with the theoretical SAXS profiles for solution-state models of ds RNA (red) and ssRNA (light red) shown in panel C. SAXS fits are shown together with the fit residuals and goodness of fit values (χ^2). Guinier plots for experimental SAXS curves are shown in the inset. (*C*) Solution state models for dsRNA and ssRNA were used to fit experimental data shown in panel B.

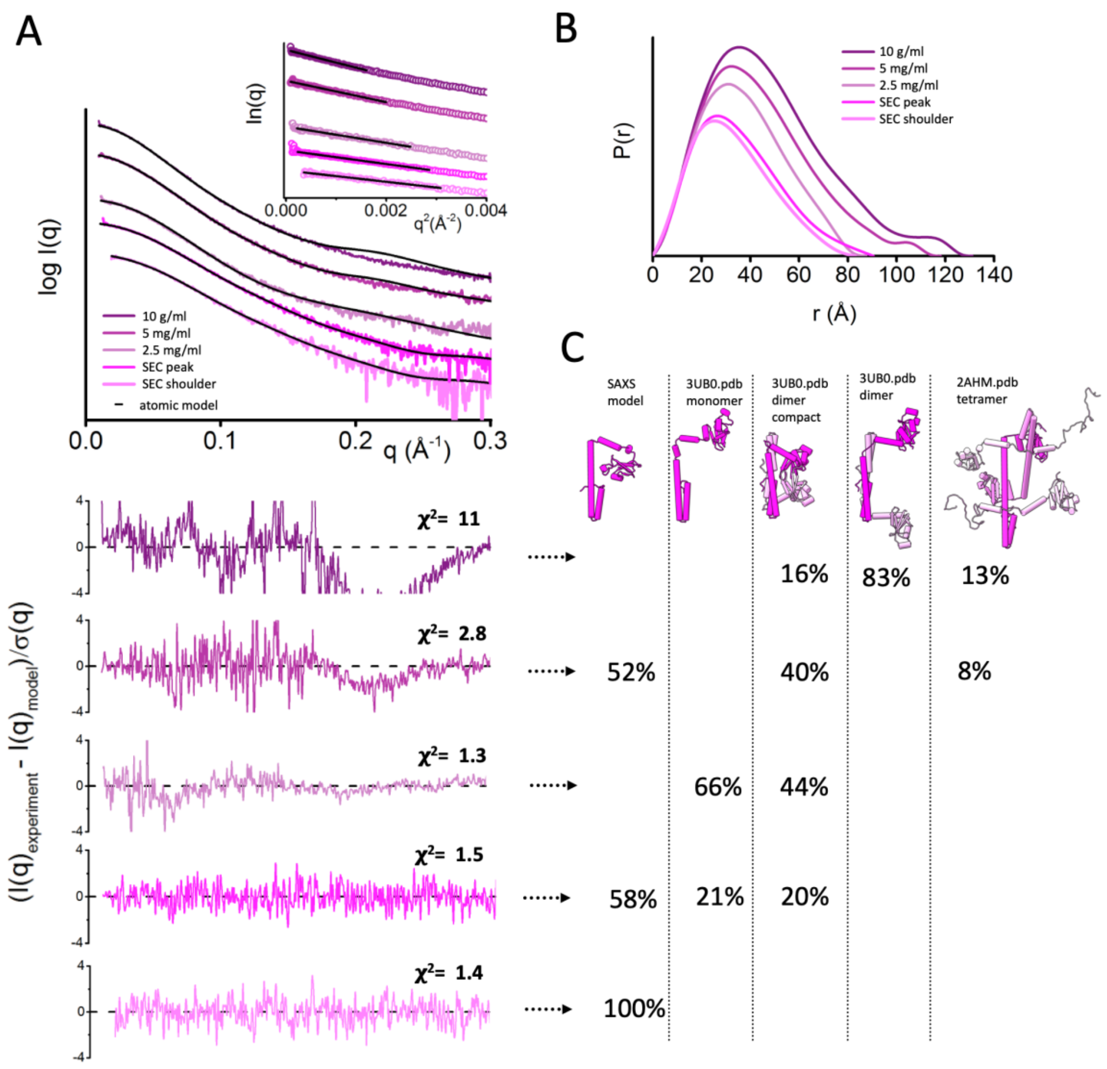

Figure S4 Nsp8 forms a dimer and larger oligomer at high concentration. (*A*) Experimental SAXS profiles for Nsp8 monomer, dimer, and the sizeable oligomeric state collected at SEC-SAXS elution shoulder and peak compared to the SAXS data collected in HT-SAXS mode at 2.5, 5, and 10 mg/ml (colored as indicated). SAXS data are shown together with a theoretical SAXS profile for multistate models shown in panel C and weights as indicated. SAXS fits are shown together with the fit residuals and goodness of fit values (χ^2). Guinier plots for experimental SAXS curves are shown in the inset. (*B*) P(r) functions calculated for the experimental SAXS profiles from panel A are normalized on the molecular mass determined by the volume of correlation Vc (41) (*C*) Solution state models for Nsp8 at various concentrations that fit experimental data shown in panel A.

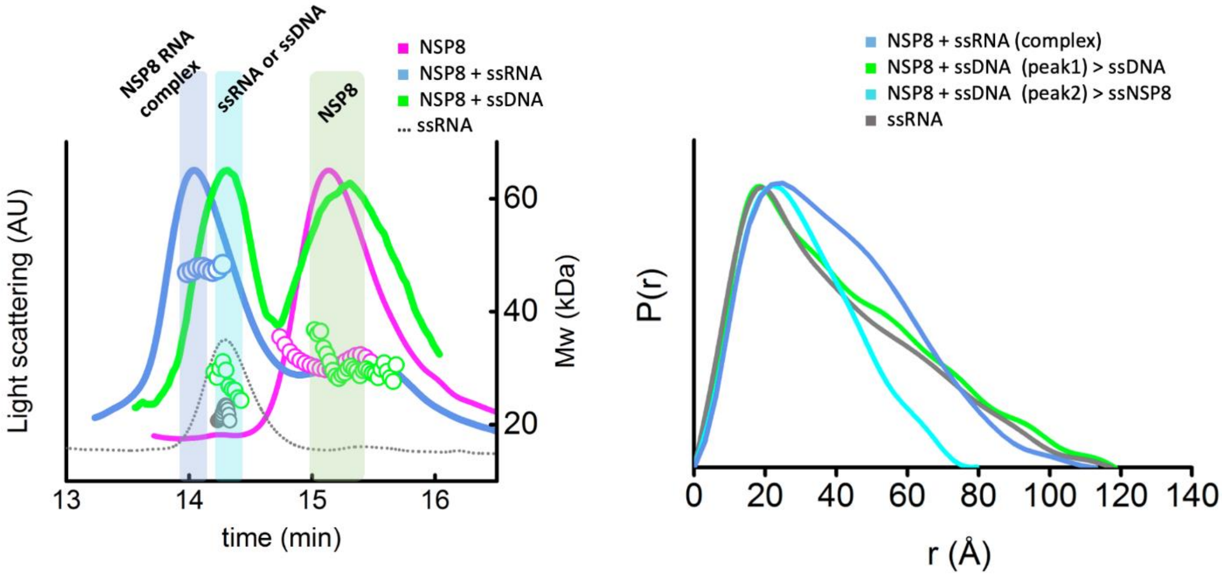

FIGURE S5 Nsp8 binds ssRNA but not ssDNA. (left) SEC-MALS-SAXS elution profiles of Nsp8, ssRNA, Nsp8 with ssRNA, and Nsp8 with ssDNA. The ssRNA is an analogous sequence and has the same length as the ssDNA. An analysis of the MALS data in each peak provides a mass as indicated by the circular points with values indicated on the right axis. The SAXS curves that were extracted from each peak containing either ssDNA or ssRNA are shown (left). The Nsp8 + ssRNA is distinct from all other curves.

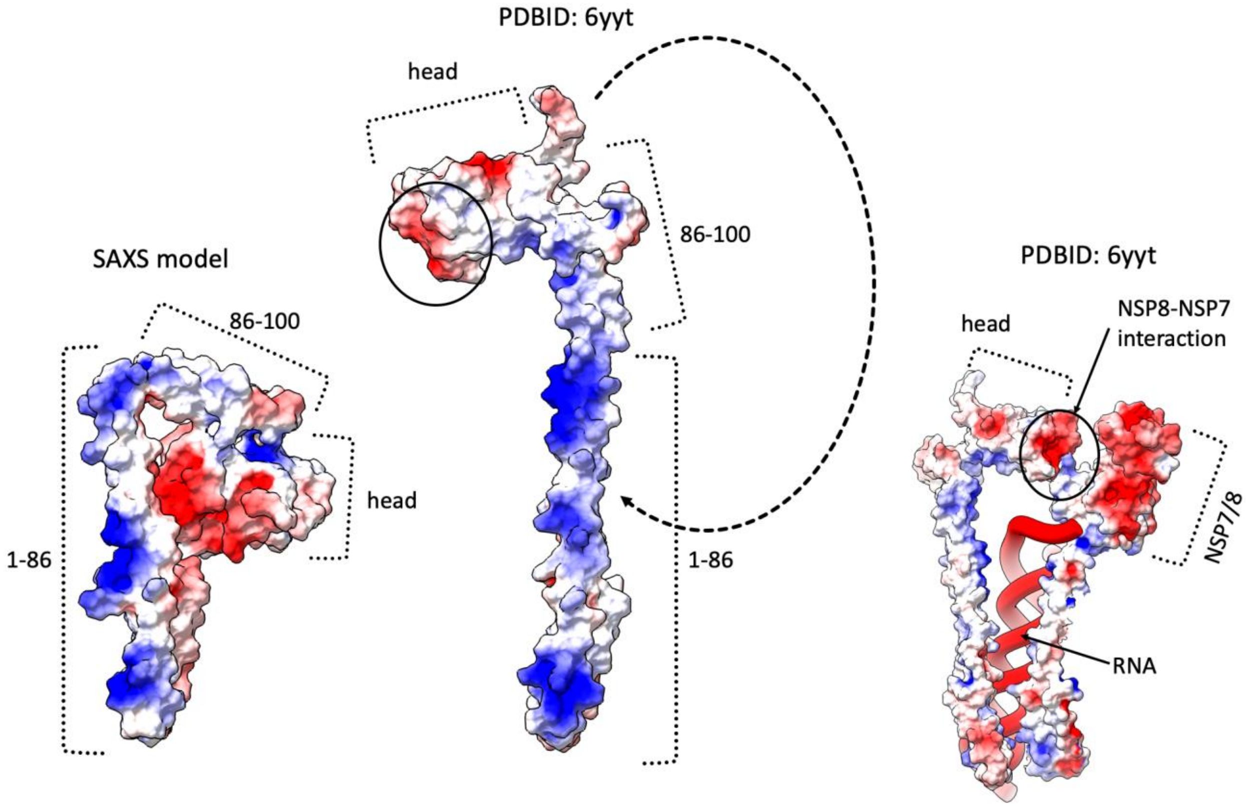

FIGURE S6 Distribution of electrostatic surface potential and flexibility of the Nsp8 helix bundle drive the compaction of Nsp8. Electrostatic surface potential for Nsp8 for SAXS model and Nsp8 conformer taken from the cryo-EM structure of Nsp7/8/12/dsRNA (PDBID: 6yyt) calculated at the pH= 7.0. The surface potential indicates that the flexible N-terminal helix bundle region (1-100) is mostly positively charged and is suited for nucleic acid-binding. In the absence of Nsp7 or Nsp12 (right panel), the negatively charged head region folds back (SAXS model) and interacts with the positively charged helix-bundle region.

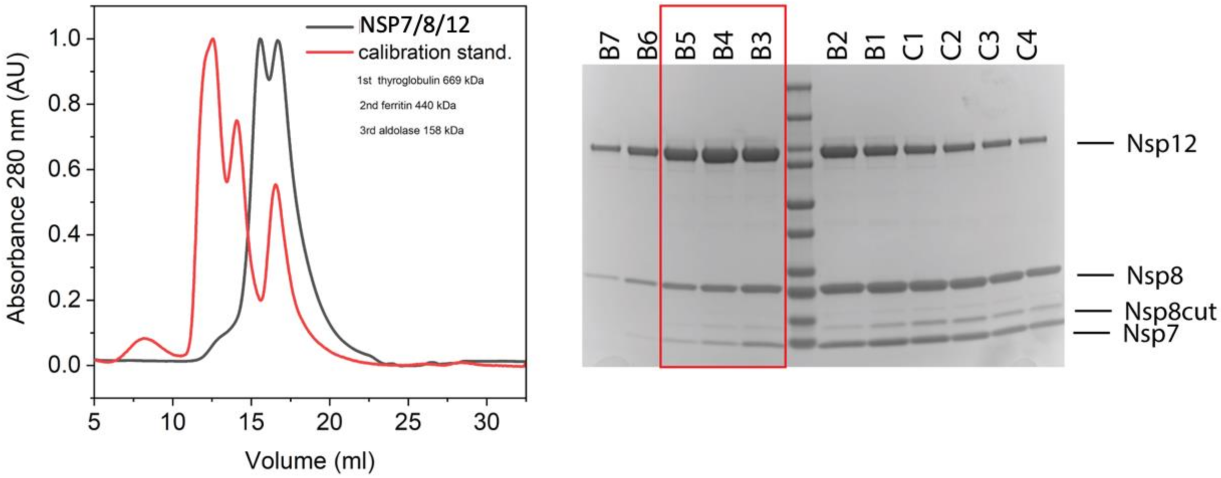

FIGURE S7 Initial SEC purification of Nsp7/8/12 sample. Splitting of SEC peak (left panel) shows heterogeneity of Nsp7/8/12 complex. The shift of Nsp8, Nsp7 bands relative to the Nsp12 on the SDS-PAGE gel (right panel) further indicates weak Nsp7/8/12 complex.
